# Italian standardization of the BPSD-SINDEM scale for the assessment of neuropsychiatric symptoms in persons with dementia

**DOI:** 10.3389/fneur.2024.1455787

**Published:** 2024-11-21

**Authors:** Federico Emanuele Pozzi, Fabrizia D'Antonio, Marta Zuffi, Oriana Pelati, Davide Vernè, Massimiliano Panigutti, Margherita Alberoni, Maria Grazia Di Maggio, Alfredo Costa, Sindem BPSD Study Group, Allegri Nicola, Appollonio Ildebrando, Badiali Vanessa, Borsani Carolina, Francesca Caso Giuseppe Bruno, Castiglioni Stefania, Canevelli Marco, Ramusino Matteo Cotta, Ferrarese Carlo, Gaillet Angelo Giovanni, Imbimbo Camillo, Solano Jorge Navarro, Magnani Giuseppe, Manfredi Luigi Giovanni, Marra Camillo, Milia Antonio, Negro Giulia, Perini Giulia, Poloni Tino Emanuele, Pomati Simone, Quaranta Davide, Scanu Lucia, Monti Micaela Sepe, Tentorio Tiziana, Lucio Tremolizzo, Elisabetta Farina

**Affiliations:** ^1^Fondazione IRCCS “San Gerardo dei Tintori”, Monza, Italy; ^2^Milan Center for Neuroscience (NeuroMI), Milan, Italy; ^3^School of Medicine and Surgery, University of Milano-Bicocca, Monza, Italy; ^4^Department of Human Neurosciences, Sapienza University of Rome, Rome, Italy; ^5^Cognitive and Motor Rehabilitation and Neuroimaging Unit, Santa Lucia Foundation (IRCCS Fondazione Santa Lucia), Rome, Italy; ^6^Department of Neurology, MultiMedica Castellanza, Castellanza, Italy; ^7^Department of Neurology, IRCCS Fondazione Don Carlo Gnocchi, Milan, Italy; ^8^Istituti Riuniti Airoldi e Muzzi Onlus Lecco, Lecco, Italy; ^9^Unit of Behavioral Neurology, IRCCS Mondino Foundation and University of Pavia, Pavia, Italy

**Keywords:** BPSD, dementia, Alzheimer’s disease, psychometric, neuropsychiatric inventory

## Abstract

**Introduction:**

Behavioral and Psychological Symptoms of Dementia (BPSD) are a heterogeneous set of psychological reactions and abnormal behaviors in people with dementia (PwD). Current assessment tools, like the Neuropsychiatric Inventory (NPI), only rely on caregiver assessment of BPSD and are therefore prone to bias.

**Materials and methods:**

A multidisciplinary team developed the BPSD-SINDEM scale as a three-part instrument, with two questionnaires administered to the caregiver (evaluating BPSD extent and caregiver distress) and a clinician-rated observational scale. This first instrument was tested on a sample of 33 dyads of PwD and their caregivers, and the results were qualitatively appraised in order to revise the tool through a modified Delphi method. During this phase, the wording of the questions was slightly changed, and the distress scale was changed into a coping scale based on the high correlation between extent and distress (*r* = 0.94). The final version consisted of three 17-item subscales, evaluating BPSD extent and caregiver coping, and the unchanged clinician-rated observational scale.

**Results:**

This tool was quantitatively validated in a sample of 208 dyads. It demonstrated good concurrent validity, with the extent subscale correlating positively with NPI scores (*r* = 0.64, *p* < 0.001) and the coping subscale inversely correlating with NPI distress (*r* = −0.20, *p* = 0.004). Diagnosis (Lewy body dementia and frontotemporal dementia), medication (antidepressants and antipsychotics), caregiver, and PwD age predicted BPSD burden on the BPSD-SINDEM scale. Caregiver coping was influenced by diagnosis (Alzheimer’s and Lewy body dementia) and benzodiazepine.

**Discussion:**

The BPSD-SINDEM scale offers a more comprehensive approach compared to NPI, by combining caregiver ratings with clinician observations. The design of the scale allows for rapid administration in diverse clinical contexts, with the potential to enhance the understanding and management of BPSD.

## Introduction

Behavioral and Psychological Symptoms of Dementia (BPSD) are a heterogeneous set of psychological reactions, psychiatric symptoms, and abnormal behaviors in persons with dementia (PwD) (1). These symptoms variably affect people with Alzheimer’s disease (AD), vascular dementia, Lewy body dementia (LDB), and frontotemporal dementia (FTD) (2) and can even be part of the diagnostic criteria for certain forms of dementia (3). These symptoms have been also reported in mild cognitive impairment (MCI) (4), contributing to both cognitive and functional decline (5). BPSD can contribute to a faster disease progression, but their occurrence has also been associated with greater cognitive impairment during moderate dementia stages, while they can disappear in late stages (6–8). Although their etiopathogenesis is complex, BPSD likely result from several factors, including genetic, personality, social and biological features, and environmental triggers (9).

BPSD have been associated with a worse quality of life (10), a negative influence on aspects of daily life (11), and an increased caregiver burden (12), as well as a considerable impact on healthcare costs due to more frequent outpatient service and emergency room visits and earlier institutionalization (9).

The variety and fluctuating nature of symptoms complicate their prevention and management: one single treatment approach does not exist. Early and accurate detection of BPSD is crucial, as well as tailored interventions (13). Currently, there are several available instruments for BPSD evaluation. The Neuropsychiatric Inventory (NPI) (14), the Revised Memory and Behavior Problems Checklist (RMBPC) (15), and the BEHAVE-AD (16) have been recommended to assess both dementia behavioral problems and BPSD changes along the disease course (9, 17).

The NPI is possibly the most used tool in clinical practice and clinical trials (17). It originally included 10 neuropsychiatric items: delusions, hallucinations, dysphoria, anxiety, agitation/aggression, euphoria, disinhibition, irritability, apathy, and aberrant motor activity. Nighttime behavioral disturbances and appetite/eating changes have subsequently been added (14, 18). The NPI, however, may not capture the full complexity of certain symptoms, as it relies on fixed response categories and may not be sensitive to subtle variations in behaviors (17, 19). Furthermore, its psychometric properties, particularly the internal consistency, factor structure, and responsiveness, have been discussed as well as its clinical utility, as it might be unable to sensitively discriminate between different behavioral disorders (19).

The RMBPC is a 24-item checklist referring to the previous week. It provides a total score and 3 scores for frequency of symptoms due to memory problems, depression, and disruptive behaviors, as well as related scores for caregiver reaction for each of these subscales. It has good reliability and validity, it can be used for longitudinal evaluations, and it has been validated for use with ethnically diverse caregivers (20). However, it focuses primarily on memory and behavioral problems, while other neuropsychiatric symptoms, such as delusions and hallucinations, are not thoroughly covered.

The BEHAVE-AD is a structured tool to assess behavioral disturbances in AD. BEHAVE-AD is administered to caregivers and addresses symptoms of the past 2 weeks. It focuses on potentially treatable symptoms to assess the effectiveness of pharmacological and non-pharmacological interventions. It evaluates seven delusional categories as well as other BPSD. A version including BPSD direct observation, the Empirical BEHAVE-AD, was developed, with an excellent inter-rater reliability (21). However, the BEHAVE-AD is primarily designed for AD and may not be easily applicable to other types of dementia.

Most of the aforementioned tools have limitations, such as the long time and difficulties in the administration, a construct that does not allow to group symptoms in clusters as well as the lack of a clinician’s observation.

Thus, most of these instruments are based on information obtained by caregivers. This may significantly affect BPSD evaluation, which may be influenced by caregivers’ own perception, memory, or emotional state. Factors such as the caregivers’ living situation and their relationship with PwD may lead to over- or underestimation of certain symptoms (22). Moreover, none of these instruments is suitable for use in the home care settings where BPSD assessment is mostly based only on qualitative observations, and this makes it difficult to manage and verify the efficacy of the treatments.

Despite their wide use, none of these instruments can be considered satisfactory in capturing BPSD phenomena entirely, and none of them considers caregiver’s coping skills.

The absence of adequate tools to measure BPSD affects the correct identification of these symptoms and their management. A good instrument should be not too long, easy to understand and administer, and treatment-oriented, providing the clinicians with data such as frequency, severity, and triggers of behaviors, and helping find appropriate strategies to effectively handle BPSD (23).

This study aimed to develop and provide the Italian standardization of a new tool for BPSD assessment that evaluates BPSD extent through both the clinician’s observation and caregiver’s perception and also investigates caregiver’s coping skills. This scale assesses in more detail many BPSD not identified by the classic NPI, e.g., sundowning. Moreover, it is designed to capture the caregiver’s coping skills in order to help caregivers in the management of BPSD. Finally, this scale aims to capture the discrepancy between the caregiver’s perception and the real observations of the BPSD in clinical settings.

## Materials and methods

### Working group composition and first phase

The working group included neurologists and neuropsychologists with a wide range of clinical experience, working in Italian secondary and tertiary memory clinics. A nursing home geriatrician was included to provide advice on potential pitfalls in applying the scale to institutionalized PwD.

The BPSD-SINDEM scale was initially drafted by EF as a three-part instrument, with two 17-item caregiver questionnaires (extent and stress) and one observational scale completed by the clinician. Each item contained an extensive description of potential instances of a neuropsychiatric variable, without any header to avoid influencing caregivers, who were instructed to rate the extent of each behavior and their relative distress on a graduated visual scale (ranging from 0 to 10). The observational scale included the same items, with slightly different descriptors, more suitable to identify observable behaviors. The clinician rated each item on a graduated visual scale (0–10), considering both the severity and frequency of each behavior during the visit. Half points were allowed.

In summary, the caregiver fills out two questionnaires during the visit, one to evaluate the extent of BPSD and the other to evaluate the associated distress. The clinician observed the behavior of the PwD during the visit and completed the observational scale. This instrument was tested in a memory clinic setting; therefore, the observational period coincided with the outpatient visit.

Each scale included the following items: apathy, depression, anxiety, obsessive behaviors, agitation, purposeless behaviors, verbal aggression, physical aggression, irritability, delusions, hallucinations, euphoria, disinhibition, sleep/wake disturbances, repetitive questions, eating disturbances, and environmental dependency (including sundowning phenomena and circadian and seasonal variations).

This original scale was tested on a heterogeneous sample of 33 PwD. Based on this experience, also taking into account caregivers’ feedback on the scale, the working group revised the caregiver subscale through a modified Delphi method. Briefly, each member was asked to anonymously vote on specific descriptive sentences to be retained within each item. Then, they received a summary of the first-round results and a proposed revised scale including only descriptors with at least 50% of votes. During a subsequent meeting, the group discussed the results and drafted the definitive version. The distress scale was transformed into a coping scale, rated on a graduated visual scale (ranging from 0 to 5: higher scores indicate higher coping). This was supported by caregivers’ difficulties in distinguishing between extent and stress, as shown by the high correlation coefficient between these two subscales in the first version (*r* = 0.94, 95% CI: 0.88–0.97, *p* < 0.0001), and the almost identical distributions of the scores (mean extent 53.2 ± 33.7 vs. mean stress 48.5 ± 35.2, *p* = 0.579).

The final instrument is a broad BPSD assessment tool comprising three 17-item subscales: extent and coping subscales administered to the caregiver and an unchanged observational scale. The scales are presented in Supplementary material.

### Sample size calculation

A sample size was calculated based on a conservative estimation of Pearson’s correlation coefficient of 0.27 between the BPSD-SINDEM severity scale and the NPI, analogously to the Italian standardization of the RMBPC (24), which was judged to be not too dissimilar from our tool. We used the following formula:


$$N=\left(\frac{z_{\propto }+z_{\beta }}{0.5*\ln\frac{1+r}{1-r}}\right)2+3$$

where *z* was the standard normal deviation of the error *α* and *β* (25). Considering *α* = 0.01, *β* = 0.05 (*z* = 2.325 and 1.645, respectively), and *r* = 0.27, a sample size of 208 subjects was obtained.

### Data collection and statistical analysis

For each participant, we collected demographical data, education, diagnosis, and living situation (home, daycare, and institutionalization). For the caregiver, we collected demographical data, education, relationship and cohabitation with PwD, principal caregiver status, presence of other caregivers, and their roles. We recorded any use of antidepressants, antipsychotics, and benzodiazepines at the time of the visit and calculated fluoxetine (26), olanzapine (27), and diazepam dose equivalents (28). The following scales were also used: CDR, NPI, MMSE (corrected for age and education), ADL and IADL, and CIRS.

In the statistical analysis, diagnoses were coded into five groups: MCI, vascular, AD, LBD (including dementia with Lewy bodies and Parkinson’s disease dementia), FTD, and others (such as atypical parkinsonism). Caregiver–PwD relationship was coded as son/daughter, partner, or other. Other caregivers’ role was coded as relatives or professionals (if both were present, it was coded as “professionals”). Birthplace was coded as Northern, Central, or Southern Italy.

Descriptive statistics are reported as percentages or mean ± standard deviation. Chi-square, *t*-test, and ANOVA with *post-hoc* tests were performed as appropriate. Concurrent validity was tested through Pearson’s r correlation between BPSD-SINDEM subscales and NPI. Correlation coefficients were calculated between BPSD-SINDEM subscales. Finally, for each BPSD-SINDEM subscale, we ran a number of linear models to identify possible predictors, after checking for collinearity. For all tests, we considered significant *p* < 0.05. Statistical analysis was conducted with SPSS version 28.

Contrary to what is commonly done, we specifically avoided calculating Cronbach’s *α* or other measures of internal consistency for the BPSD-SINDEM scale, as it is not a unidimensional construct and no internal consistency should be expected (23). As we only had baseline visits with a single rater, we could not provide estimates for inter-rater and test–retest reliability.

### Instruments and scales

The MMSE is a screening battery widely used in the field of dementia, representing a rapid and sensitive tool to assess the general level of deterioration and changes over time (29). The CDR is a global assessment tool to quantify the severity of dementia. It assesses six domains of cognitive and functional performance: memory, orientation, judgment and problem-solving, community affairs, hobbies, and personal care (30). It requires both the presence of an informant and an assessment of the patient’s cognition.

The version of NPI used in this study is the NPI-12 (18). The questions refer to behaviors over the previous month, including only changes arising after the onset of the disease. The disturbances are investigated through a screening question; if an affirmative answer is obtained, further questions are asked. Some sections may not be applicable due to the presence of medical conditions interfering with the answers. For each question, the informant must define the behavior frequency and severity and the relative personal distress.

The ADL scale evaluates the subject’s autonomy in the usual daily tasks, concerning their own hygiene, nutrition, continence, and mobility (31). The IADL scale assesses the ability to carry out activities that are normally carried out by elderly subjects and which are considered necessary for maintaining one’s independence (32). They correlate with cognitive decline, and therefore, they can be useful in both identifying subjects with dementia and clinical follow-up to evaluate the efficacy of pharmacological treatment.

The CIRS is used to measure the health of the general elderly as objectively as possible (33). This tool requires the clinician to define the clinical and functional severity of 14 disease categories, based on clinical history and physical examination. It includes a severity index (mean score across the first 13 items, ranging from 1 to 5) and a comorbidity index (number of categories with a score of 3 or more).

## Results

### Sample characteristics

The final sample comprised 208 dyads. Summary statistics are reported in Tables 1, 2. The average PwD suffered from moderate dementia and lived at home. Significantly more PwD were born in Northern compared to Central Italy. Female PwD were significantly older and less educated. Caregivers were significantly more females, primary caregivers, and either sons/daughters or partners of the PwD and supported by the presence of other non-professional caregivers. PwDs with AD were significantly more represented than PwDs with other diagnoses. The majority of PwD were on antidepressants, while only a minority were on antipsychotics or benzodiazepines.

**Table 1:** Summary statistics of the recruiter sample, categorical variables.

Variable	Options	Count (percentage)	*p*-values
Center	Milan (Don Gnocchi)	52 (25.0%)	1
	Monza	65 (31.3%)	0.049
	Castellanza	41 (19.7%)	0.087
	Rome	50 (24.0%)	0.820
Region of birth	North	112 (54.9%)	< 0.001
	Center	33 (16.2%)	< 0.001
	South	59 (28.9%)	0.243
Gender of patient	Male	91 (43.8%)	0.083
	Female	117 (56.2%)	
Diagnosis	MCI	22 (10.6%)	0.013
	Vascular dementia	20 (9.6%)	0.004
	AD	104 (50%)	< 0.001
	LBD	37 (17.8%)	0.818
	FTD	15 (7.2%)	< 0.001
	Other	10 (4.8%)	< 0.001
Gender of caregiver	Males	59 (28.5%)	< 0.001
	Females	148 (71.5%)	
Caregiver/PwD relation	Son/daughter	108 (51.9%)	< 0.001
	Partner	88 (42.3%)	0.006
	Other	12 (5.8%)	< 0.001
Cohabitation	Yes	113 (54.3%)	0.238
	No	95 (45.6%)	
Primary caregiver	Yes	152 (73.1%)	< 0.001
	No	56 (26.9%)	
Other caregivers	Yes	133 (63.9%)	< 0.001
	No	75 (36.1%)	
Other caregivers’ role	Professional	23 (17.3%)	< 0.001
	Relatives	110 (82.7%)	
Living situation	Daycare	11 (5.3%)	< 0.001
	Home	197 (94.7%)	
BPSD drugs	Yes	118 (56.7%)	0.061
	No	90 (43.3%)	
Antidepressants	Yes	138 (66.4%)	< 0.001
	No	70 (33.65%)	
Antipsychotics	Yes	54 (25.9%)	< 0.001
	No	154 (74.1%)	
Benzodiazepines	Yes	32 (15.4%)	< 0.001
	No	176 (84.6%)	

Test of proportions. When only two levels of a variable are present, only one *p*-value is shown.

**Table 2:** Summary statistics of the recruiter sample, continuous variables.

Variable	Mean ± SD	Female (mean ± SD)	Male (mean ± SD)	*p*
PwD age	77 ± 7	78.4 ± 6.1	74.9 ± 7.9	< 0.001
PwD education	9 ± 4	8.1 ± 3.5	9.4 ± 3.9	0.009
Caregivers age	60 ± 13	59.0 ± 11.8	62.7 ± 15.1	0.101
Caregivers education	13 ± 4	12.8 ± 4.2	12.7 ± 4.2	0.828
Duration of visit (min)	54 ± 27			
MMSE raw	18 ± 7			
MMSE corrected	18 ± 8			
ADL	4 ± 2			
IADL	3 ± 3			
CDR	2 ± 1			
CIRS severity index	1.4 ± 0.4			
CIRS comorbidity index	1 ± 2			
Fluoxetine equivalents	5.3 ± 9.7			
Olanzapine equivalents	0.5 ± 1.4			
Diazepam equivalents	0.7 ± 2.9			
BPSD-SINDEM severity	60.4 ± 33.2			
BPSD-SINDEM coping	55.3 ± 22.7			
BPSD-SINDEM observational	17.2 ± 16.4			
NPI frequency x severity	33 ± 21			
NPI caregiver stress	16 ± 11			

*t-tests.*

The BPSD-SINDEM extent subscales (caregiver and observational) were significantly and positively correlated (*r* = 0.34, *p* < 0.001), meaning that the BPSD burden at home may be effectively estimated during the visit by observing the PwD’s behavior. The BPSD-SINDEM extent subscale did not significantly correlate with the coping subscale (*r* = 0.04, *p* = 0.54).

The characteristics of the sample in which the first version of the scale was tested were similar to the sample for the second version (data not shown).

### Concurrent validity

Both BPSD-SINDEM extent scales positively correlated with NPI frequency * severity scores (BPSD-SINDEM caregiver *r* = 0.64, *p* < 0.001, BPSD-SINDEM observational *r* = 0.45, *p* < 0.001). The BPSD-SINDEM coping subscale inversely correlated with NPI caregiver stress (*r* = −0.20, *p* = 0.004). This was expected according to the design of the scale, hypothesizing that higher stress would correlate with reduced coping. Albeit significant, the relatively small coefficient suggests that stress is not the only coping determinant.

### Determinants of BPSD burden

We ran several linear models to estimate possible predictors of BPSD-SINDEM subscale scores. There was no collinearity within any model.

In the first model, we included the region of birth, diagnosis, demographic variables, CDR, MMSE, usage of drugs for BPSD, and primary caregiver status. A diagnosis of LBD or FTD and the usage of BPSD drugs, in particular antidepressants and antipsychotics, were significant predictors of higher BPSD-SINDEM severity scores, whereas a higher caregiver age predicted lower BPSD burden across all models (models *R*^2^ = 0.27–0.30, Supplementary Tables S1, S2). In one model, higher CDR and PwD age predicted higher BPSD-SINDEM severity scores, but this was not significant in other models. Fluoxetine equivalents significantly predicted BPSD burden (Supplementary Table S2).

To test why higher caregiver age resulted in lower BPSD-SINDEM severity scores, we performed *t*-tests by grouping for PwD/caregiver cohabitation and PwD/caregiver relationship (son/daughter vs. partner). We hypothesized that being a partner and living with the PwD, conditions that were significantly associated with older caregiver age (data not shown), would result in a lower BPSD burden. However, we found no significant differences in BPSD-SINDEM extent according to either of these variables, meaning that caregiver age results in lower BPSD burden independently of cohabitation or relation type.

We also run models with only psychotropic drugs classes, again finding an increased burden of BPSD for antidepressants and antipsychotics usage, and for higher fluoxetine and olanzapine equivalent doses. These models expectedly explained less BPSD-SINDEM extent variance compared to the first ones (models *R*^2^ = 0.08–0.16, Supplementary Tables S4, S5).

### Determinants of caregiver coping

We ran a first series of linear models with diagnosis, caregiver demographic variables, CDR, MMSE, usage of psychotropic drugs, presence of other caregivers, CIRS severity, ADL, caregiver status (primary), and BPSD-SINDEM extent as predictors. We found that a diagnosis of AD or LBD significantly predicted reduced coping across all models (models *R*^2^ = 0.08–0.11, Supplementary Tables S6, S7). Usage of benzodiazepines was consistently predictive of higher caregiver coping, but diazepam equivalents were not (Supplementary Table S7–S9).

### Determinants of BPSD-SINDEM observational scores

We ran linear models with region of birth, diagnosis, PwD demographic variables, visit duration, CDR, MMSE, and usage of BPSD drugs as predictors of BPSD-SINDEM observational scores. We found that being born in Central Italy, being diagnosed with LBD or FTD, and being more globally impaired on CDR predicted a higher burden of BPSD during the visit. However, only being born in Central Italy and CDR were consistent predictors (models *R*^2^ = 0.19–0.20, Supplementary Tables S10, S11).

Only the use of antidepressants, but not their dose, was predictive of higher BPSD-SINDEM observational scores (Supplementary Tables S12, S13).

### Differences among centers

Caregivers recruited at Don Gnocchi and in Rome were more likely to live with the PwD; the opposite was true for Monza and Castellanza. PwD at Don Gnocchi were more likely to attend daycare than PwDs in other centers. PwD at Don Gnocchi were significantly older and more functionally and cognitively impaired, while PwD in Rome had significantly higher education. PwD at Don Gnocchi also had a significantly higher burden of BPSD on NPI compared to PwD in other centers. There were significant differences among caregivers’ age and education, with caregivers at Don Gnocchi being significantly older than those in Monza, and more educated in Monza than in Castellanza. No significant differences according to birthplace were noted (Supplementary Tables S14, S15).

## Discussion

BPSD are a major source of burden for caregivers and PwD (12, 34). Their consequences often overcome those of lack of independence in everyday life: families more easily adjust to providing everyday care to PwD than to receiving verbal or even physical aggression from their loved ones, or seeing them depressive or apathetic, or to not being allowed to sleep at night (2). Anxiety, depression, hallucinations, and even more aggression and delusions represent a major cause of sufferance for PwD (35). Wandering can be a cause of falls (36); anorexia or hyperphagia can worsen general health (37, 38); and lack of sleep can favor other BPSD in a vicious circle (39).

The availability of efficient tools to measure BPSD is a priority in dementia care. A clear and complete picture of BPSD variety and severity can allow professionals to plan efficient interventions to reduce their burden for caregivers and PwD. These should include non-pharmacological interventions as first-line treatments, and drugs as second-line approaches, due to incomplete evidence of efficacy, multiple severe side effects (40), and the fact that neuroleptics may be considered a pharmacological restraint. We must always consider the dignity and rights of PwD, and an efficient way to measure BPSD can allow a careful and ethically correct use of drugs.

While a precise assessment of BPSD is mandatory, available tools for measuring BPSD are far from perfect. An ideal instrument should be easy and rapid to administer and cover at the same time all the different aspects of BPSD. It should consider different points of view and give professionals useful information to rapidly plan interventions (23).

However, the most available ones only consider caregivers’ perspectives. If their point of view is obviously essential, different caregiver biases may alter BPSD perception; e.g., caregiver personality, previous kind of relationship with the PwD, and lack of education about symptoms of cognitive and behavioral decline and how to treat them (41, 42). In our opinion, an ideal instrument should flank a tool based on the caregiver’s point of view with another tool measuring BPSD in another way, to reduce wrong interventions based on caregiver bias. Therefore, following the example of BEHAVE-AD (43), we decided to create a tool with two BPSD measures: a caregiver questionnaire and a scale based on direct observation of PwD. Our data show an acceptable correlation between them, demonstrating that they measure the same construct, without being redundant. Indeed, the availability of two possibly divergent scales might allow the clinician to formulate hypotheses regarding the reliability of the caregiver, or uncover potential issues in the relationship between the caregiver and the PwD. The observational scale is very easy and rapid to administer; therefore, it does not interfere with the clinical examination or the activity.

For the caregiver tool, we chose the questionnaire format instead of a scale administered by the personnel, due to the need of saving time in the Italian clinical context, where ambulatory and residential services often struggle with a lack of resources. If we want to correctly administer NPI^14^ in the case of a person with a lot of BPSD, we need to score for frequency and severity all single subitems with positive screening questions. This is time-consuming and may lead to avoiding NPI or administering it in an inappropriate way (with the risk of mischaracterizing the problem). Moreover, a questionnaire is more flexible, allowing completion at home or while waiting for the visit. Personnel intervention may be necessary to provide clarification to caregivers, above all those with a low education, but it is very limited in time.

In terms of concurrent validity, in our caregiver tool extent shows satisfactory correlation with NPI frequency * severity. A very high correlation was not actually expected as we added some aspects not assessed in NPI, such as BPSD temporal variation (to pick the occurrence of sundowning) or repetitive questioning. Sundowning is highly prevalent in PwD, and its identification is a major clinical need, as it deserves specific non-pharmacological and pharmacological interventions. Indeed, temporal variations in BPSD are usually ignored by most available tools. We are aware of the fact that repetitive questioning is not, strictly speaking, a “true” BPSD, being instead the direct consequence of anterograde memory impairment. However, it is perceived as a BPSD by caregivers: therefore, we chose to add a specific question in our questionnaire [following the example of RMBPC (15)]. Identifying this problem may also allow specific non-pharmacological interventions.

We decided to ask caregivers to score disturbing behaviors according to a “global” gestalt measure (“extent”), rather than rating both frequency and severity (as in NPI and BEHAVE-AD) because we think that a global measure is simpler and faster to score. Indeed, caregivers are often reluctant to precisely score the timing of BPSD during a week or show difficulties in understanding the right meaning of terms such as “mild,” “moderate,” or “severe.”

We distinguished agitation from aggression (at variance with NPI^14^), as we think that they are different concepts, and verbal aggression from physical one, as we believe that the latter frequently needs a pharmacological approach (at least in an emergency), while in the former non-pharmacological interventions can be often effective.

In the first version of our caregiver tool, we introduced a measure of distress, following NPI and RMBPC examples. However, the distress measure was then changed to a coping one for two reasons: the observed high correlation between the extent and distress in the preliminary phase, and the idea that a measure oriented toward caregiver support could be more useful for planning effective interventions in clinical practice. Our coping questionnaire negatively correlated with caregiver distress on NPI, as expected. It did not correlate with the BPSD extent measure, but this is not surprising: coping abilities mainly depend on other variables, such as education on the disease, caregiver personality, and supporting network (44). A measure of the caregivers’ coping abilities might be helpful to clinicians in highlighting and providing caregivers the best strategies for BPSD management, which in some cases might entail placing the PwD in daycare centers or nursing homes. This might improve BPSD management, and consequently patient’s wellbeing. Moreover, it may be useful to establish the need for interventions aimed at supporting the caregiver.

AD was the most represented diagnosis in our sample. However, we included also other forms of dementia, and we tried to assess a large range of BPSD, as our tool was planned to be used in all forms of neurocognitive disorder. We also refined the first version of our tool with the help of an experienced geriatrician working in an Alzheimer’s unit. Even if this validation study was carried out in memory clinics, we wanted to create a tool suitable for different clinical contexts and dementia severity. A preliminary study applying our scale in residential Alzheimer’s units is in progress.

Differences were noted in demographical and experimental features across centers. Caregivers living in Rome showed higher distress at NPI for an almost equal score of frequency * severity than Lombardy centers. These could be due to cultural differences: probing our scale in different geographical settings, like Southern Italy, will be our next step.

A diagnosis of LBD or FTD significantly predicted higher BPSD-SINDEM extent: this is not surprising (45, 46). More surprising is the fact that higher caregiver age predicted a lower BPSD extent across all linear models. We first hypothesized that spouses could be more indulgent than children in scoring BPSD, due to the different kinds of relationships, but further data analysis excluded this explanation. Maybe aging makes people generally more indulgent. An alternative explanation may be that children are part of the so-called sandwich generation. As younger caregivers on average experience more financial and emotional difficulties (47), they tend to feel overloaded and to consider the symptoms as more severe. However, while the specific characteristics of the caregiver may influence the perception of BPSD, these hypotheses could not be tested in our cohort.

A diagnosis of AD or LBD significantly predicted reduced coping among caregivers. This finding seems reasonable in the case of LBD, which has unique BPSD features. It is less expected for AD; this suggests that a lot of work still needs to be done in improving caregiver coping abilities, even after all the efforts by different stakeholders in recent years.

BPSD extent on the caregiver scale was predicted by the use of antidepressants and antipsychotics, while on the observational scale, it was predicted by use of antidepressants. This finding supports a strong association between BPSD impact and pharmacological treatments and, again, underlines the importance of correctly investigating BPSD to avoid unnecessary pharmacological interventions.

Interestingly, caregivers’ coping was associated with benzodiazepine use, possibly mediated by the idea of having a tool to personally manage BPSD (above all sleep disturbances). However, this finding must be cautiously interpreted due to the possible severe side effects associated with the use of benzodiazepines in elderly people (48).

In general, lower *R*^2^ for coping and observational scores suggest the presence of other factors that have not been investigated. While some variables consistently predicted BPSD burden irrespective of the rater (such as LBD or FTD diagnosis, with LBD predicting also reduced coping), there seem to be different peculiar determinants for caregiver and physicians’ judgment of BPSD extent.

A limitation of our study is the lack of inter-rater and test–retest reliability, which deserves to be investigated. Moreover, despite the large sample, it is likely that many secondary analyses are indeed underpowered and need to be taken as hypothesis-generating results, rather than actual conclusions. In this study, psychometric properties, e.g., content validity and item validity, of this scale were not assessed. However, the first aim of this study was to propose a new instrument tailored on clinical experience assessing in detail BPSD. Future studies assessing the psychometric characteristics of the scale are warranted.

However, the BPSD-SINDEM scale seems a valid tool for assessing BPSD impact and caregiver coping. Our tools have additional advantages compared to the other scales used to assess BPSD. First, it actually provides a quantitative measure of the clinician’s observations and the caregivers’ perception of BPSD. Although a comparison between clinician’s and caregivers’ BPSD perception is performed in clinical contexts, it has never been quantified in a single instrument. Second, the BPSD-SINDEM scale is suitable to be used in different clinical contexts that vary from own home to residential home care. The availability of a reliable quantitative measure might be helpful in BPSD management and in the choice of treatment strategies as well as in the follow-up. Moreover, the observational scale can be compiled by psychologists, nurses, and physicians. Finally, in comparison with the NPI that requires a long time for its administration, the BPSD-SINDEM scale is an easy and intuitive tool with a rapid administration that provides at the same time a comprehensive BPSD evaluation.

The scale can be used to improve the management of BPSD and support caregivers in clinical settings, but it could be also interesting in research settings. Further studies will elucidate the sensitivity of scale to change, responsiveness to treatment, and how measures are influenced by the clinical and geographical settings.

## Data Availability

The raw data supporting the conclusions of this article will be made available by the authors, without undue reservation.

## Group member of Sindem BPSD Study Group

Nicola Allegri, Ildebrando Appollonio, Vanessa Badiali, Carolina Borsani, Giuseppe Bruno, Francesca Caso, Stefania Castiglioni, Marco Canevelli, Matteo Cotta Ramusino, Carlo Ferrarese, Angelo Giovanni Gaillet, Camillo Imbimbo, Jorge Navarro Solano, Giuseppe Magnani, Luigi Giovanni Manfredi, Camillo Marra, Antonio Milia, Giulia Negro, Giulia Perini, Tino Emanuele Poloni, Simone Pomati, Davide Quaranta, Lucia Scanu, Micaela Sepe Monti, Tiziana Tentorio.
