# Supplementary material for: Italian standardization of the BPSD-SINDEM scale for the assessment of neuropsychiatric symptoms in persons with dementia
Source: Front Neurol. 2024 Nov 21;15:1455787. doi: 10.3389/fneur.2024.1455787 (PMC11617322; doi:10.3389/fneur.2024.1455787)
Supplement: Supplementary file 1 [file Table_1.docx]

**Supplementary material**

**Table S1.** Model 1 for BPSD-SINDEM severity.

|  | | | | | | | | | | |
| --- | --- | --- | --- | --- | --- | --- | --- | --- | --- | --- |
| Model | | **Unstandardized coefficient** | | **Standardized coefficient** | **t** | **p.** | **95,0% CI** | | **Collinearity** | |
|  |  | **B** | **Standard error** | **Beta** |  |  | **Lower limit** | **Upper limit** | **Tolerance** | **VIF** |
| 1 | Intercept | 18,785 | 31,038 |  | ,605 | ,546 | -42,460 | 80,029 |  |  |
|  | RoB=Center | -5,262 | 6,386 | -,058 | -,824 | ,411 | -17,863 | 7,338 | ,825 | 1,213 |
|  | RoB=South | ,363 | 5,134 | ,005 | ,071 | ,944 | -9,767 | 10,494 | ,831 | 1,204 |
|  | Diagnosis=Vasc | 9,355 | 9,694 | ,085 | ,965 | ,336 | -9,773 | 28,483 | ,520 | 1,922 |
|  | Diagnosis=AD | 9,167 | 8,109 | ,139 | 1,130 | ,260 | -6,835 | 25,169 | ,270 | 3,702 |
|  | **Diagnosis=LBD** | **19,867** | 9,228 | ,224 | 2,153 | **,033** | 1,658 | 38,077 | ,375 | 2,664 |
|  | **Diagnosis=FTD** | **37,229** | 10,635 | ,288 | 3,501 | **<,001** | 16,244 | 58,213 | ,598 | 1,673 |
|  | Diagnosis=other | 11,784 | 12,249 | ,074 | ,962 | ,337 | -12,387 | 35,955 | ,682 | 1,466 |
|  | Patient age | ,490 | ,352 | ,101 | 1,392 | ,166 | -,204 | 1,184 | ,772 | 1,295 |
|  | Patient education | ,619 | ,653 | ,071 | ,947 | ,345 | -,671 | 1,908 | ,731 | 1,368 |
|  | Patient gender | -5,583 | 5,396 | -,084 | -1,035 | ,302 | -16,231 | 5,065 | ,619 | 1,615 |
|  | **Caregiver age** | **-,447** | ,225 | -,173 | -1,987 | **,048** | -,890 | -,003 | ,536 | 1,865 |
|  | Caregiver education | -,416 | ,639 | -,053 | -,651 | ,516 | -1,676 | ,844 | ,610 | 1,639 |
|  | Caregiver gender | -1,135 | 5,468 | -,015 | -,208 | ,836 | -11,925 | 9,654 | ,732 | 1,366 |
|  | CDR | 5,937 | 3,182 | ,182 | 1,866 | ,064 | -,342 | 12,216 | ,427 | 2,340 |
|  | MMSE raw | -,148 | ,447 | -,031 | -,331 | ,741 | -1,030 | ,734 | ,453 | 2,210 |
|  | **BPSD drugs** | **18,732** | 4,642 | ,279 | 4,035 | **<,001** | 9,573 | 27,892 | ,846 | 1,181 |
|  | Primary caregiver | 6,787 | 5,557 | ,092 | 1,221 | ,224 | -4,177 | 17,752 | ,717 | 1,395 |
| *Notes.* R^2^ = 0.27 | | | | | | | | | | |

| **Table S2.** Model 1.1 for BPSD-SINDEM severity. | | | | | | | | | | |
| --- | --- | --- | --- | --- | --- | --- | --- | --- | --- | --- |
| Model | | **Unstandardized coefficient** | | **Standardized coefficient** | **t** | **p.** | **95,0% CI** | | **Collinearity** | |
|  |  | **B** | **Standard error** | **Beta** |  |  | **Lower limit** | **Upper limit** | **Tolerance** | **VIF** |
| 1 | Intercept | 19,452 | 30,458 |  | ,639 | ,524 | -40,651 | 79,555 |  |  |
|  | RoB=Center | -3,398 | 6,308 | -,037 | -,539 | ,591 | -15,845 | 9,049 | ,814 | 1,228 |
|  | RoB=South | 1,690 | 5,048 | ,023 | ,335 | ,738 | -8,271 | 11,652 | ,828 | 1,208 |
|  | Diagnosis=Vasc | 8,395 | 9,509 | ,076 | ,883 | ,378 | -10,369 | 27,159 | ,521 | 1,920 |
|  | Diagnosis=AD | 6,322 | 8,053 | ,096 | ,785 | ,434 | -9,570 | 22,213 | ,264 | 3,790 |
|  | Diagnosis=LBD | 15,069 | 9,220 | ,170 | 1,634 | ,104 | -3,126 | 33,264 | ,362 | 2,761 |
|  | **Diagnosis=FTD** | **34,879** | 10,470 | ,270 | 3,331 | **,001** | 14,219 | 55,539 | ,594 | 1,684 |
|  | Diagnosis=other | 10,095 | 12,006 | ,064 | ,841 | ,402 | -13,596 | 33,785 | ,684 | 1,462 |
|  | Patient age | ,513 | ,345 | ,106 | 1,486 | ,139 | -,168 | 1,195 | ,771 | 1,297 |
|  | Patient education | ,558 | ,645 | ,064 | ,865 | ,388 | -,715 | 1,830 | ,723 | 1,384 |
|  | Patient gender | -4,085 | 5,299 | -,061 | -,771 | ,442 | -14,541 | 6,371 | ,618 | 1,617 |
|  | **Caregiver age** | **-,473** | ,221 | -,183 | -2,142 | **,034** | -,909 | -,037 | ,535 | 1,869 |
|  | Caregiver education | -,630 | ,632 | -,080 | -,997 | ,320 | -1,877 | ,617 | ,600 | 1,667 |
|  | Caregiver gender | 1,003 | 5,356 | ,014 | ,187 | ,852 | -9,567 | 11,573 | ,735 | 1,361 |
|  | CDR | 6,088 | 3,135 | ,186 | 1,942 | ,054 | -,098 | 12,273 | ,424 | 2,357 |
|  | MMSE raw | -,055 | ,440 | -,012 | -,124 | ,901 | -,924 | ,815 | ,449 | 2,229 |
|  | Primary caregiver | 6,740 | 5,457 | ,091 | 1,235 | ,218 | -4,029 | 17,509 | ,716 | 1,397 |
|  | **Antidepressants** | **17,351** | 4,635 | ,249 | 3,743 | **<,001** | 8,204 | 26,498 | ,883 | 1,133 |
|  | **Antipsychotics** | **18,245** | 5,260 | ,244 | 3,469 | **<,001** | 7,867 | 28,624 | ,789 | 1,268 |
| *Notes.* R^2^ = 0.30 | | | | | | | | | | |

**Table S3.** Model 1.2 for BPSD-SINDEM severity.

|  | | | | | | | | | | |
| --- | --- | --- | --- | --- | --- | --- | --- | --- | --- | --- |
| **Model** | | **Unstandardized coefficient** | | **Standardized coefficient** | **t** | **p.** | **95,0% CI** | | **Collinearity** | |
|  |  | **B** | **Standard error** | **Beta** |  |  | **Lower limit** | **Upper limit** | **Tolerance** | **VIF** |
| 1 | Intercept | 4,301 | 32,591 |  | ,132 | ,895 | -60,072 | 68,674 |  |  |
|  | RoB=Center | -8,527 | 7,077 | -,090 | -1,205 | ,230 | -22,506 | 5,451 | ,822 | 1,216 |
|  | RoB=South | 5,088 | 5,549 | ,069 | ,917 | ,361 | -5,873 | 16,049 | ,811 | 1,233 |
|  | Diagnosis=Vasc | 13,546 | 10,190 | ,126 | 1,329 | ,186 | -6,581 | 33,674 | ,502 | 1,993 |
|  | Diagnosis=AD | 9,019 | 8,775 | ,136 | 1,028 | ,306 | -8,314 | 26,352 | ,261 | 3,836 |
|  | **Diagnosis=LBD** | **20,789** | 10,168 | ,232 | 2,045 | **,043** | ,706 | 40,871 | ,353 | 2,835 |
|  | **Diagnosis=FTD** | **37,459** | 11,394 | ,295 | 3,288 | **,001** | 14,954 | 59,963 | ,565 | 1,769 |
|  | Diagnosis=other | 12,188 | 13,113 | ,076 | ,930 | ,354 | -13,711 | 38,088 | ,673 | 1,487 |
|  | **Patient age** | **,820** | ,367 | ,170 | 2,231 | **,027** | ,094 | 1,545 | ,781 | 1,280 |
|  | Patient education | ,677 | ,680 | ,077 | ,996 | ,321 | -,666 | 2,020 | ,755 | 1,325 |
|  | Patient gender | -2,135 | 5,727 | -,032 | -,373 | ,710 | -13,447 | 9,176 | ,622 | 1,608 |
|  | **Caregiver age** | **-,576** | ,240 | -,225 | -2,403 | **,017** | -1,050 | -,103 | ,518 | 1,931 |
|  | Caregiver education | -1,081 | ,711 | -,138 | -1,519 | ,131 | -2,486 | ,324 | ,550 | 1,818 |
|  | Caregiver gender | 2,125 | 5,822 | ,029 | ,365 | ,716 | -9,374 | 13,625 | ,737 | 1,357 |
|  | **CDR** | **7,335** | 3,378 | ,229 | 2,171 | **,031** | ,663 | 14,007 | ,408 | 2,452 |
|  | MMSE raw | -,034 | ,483 | -,007 | -,071 | ,944 | -,989 | ,921 | ,439 | 2,276 |
|  | Primary caregiver | 5,373 | 5,954 | ,072 | ,902 | ,368 | -6,387 | 17,133 | ,705 | 1,419 |
|  | **Fluoxetine equivalents** | **,679** | ,240 | ,205 | 2,828 | **,005** | ,205 | 1,153 | ,869 | 1,151 |
|  | Olanzapine equivalents | 3,194 | 1,623 | ,144 | 1,968 | ,051 | -,012 | 6,401 | ,844 | 1,185 |
| *Notes.* R^2^ = 0.29 | | | | | | | | | | |

**Table S4.** Model 2 for BPSD-SINDEM severity.

|  | | | | | | | | | | |
| --- | --- | --- | --- | --- | --- | --- | --- | --- | --- | --- |
| **Model** | | **Unstandardized coefficient** | | **Standardized coefficient** | **t** | **p.** | **95,0% CI** | | **Collinearity** | |
|  |  | **B** | **Standard error** | **Beta** |  |  | **Lower limit** | **Upper limit** | **Tolerance** | **VIF** |
| 1 | Intercept | 48,847 | 2,993 |  | 16,322 | <,001 | 42,946 | 54,747 |  |  |
|  | **Antipsychotics** | **25,555** | 4,900 | ,338 | 5,216 | **<,001** | 15,894 | 35,216 | ,986 | 1,014 |
|  | **Antidepressants** | **15,288** | 4,536 | ,218 | 3,370 | **<,001** | 6,344 | 24,232 | ,990 | 1,010 |
|  | Benzodiazepines | -1,634 | 5,977 | -,018 | -,273 | ,785 | -13,418 | 10,150 | ,979 | 1,022 |
| *Notes.* R^2^ = 0.16 | | | | | | | | | | |

**Table S5.** Model 3 for BPSD-SINDEM severity.

|  | | | | | | | | | | |
| --- | --- | --- | --- | --- | --- | --- | --- | --- | --- | --- |
| Model | | **Unstandardized coefficient** | | **Standardized coefficient** | **t** | **p.** | **95,0% CI** | | **Collinearity** | |
|  |  | **B** | **Standard error** | **Beta** |  |  | **Lower limit** | **Upper limit** | **Tolerance** | **VIF** |
| 1 | Intercept | 54,697 | 3,019 |  | 18,120 | <,001 | 48,737 | 60,657 |  |  |
|  | **Fluoxetine ED** | **,557** | ,255 | ,166 | 2,187 | **,030** | ,054 | 1,060 | ,968 | 1,033 |
|  | **Olanzapine ED** | **4,201** | 1,662 | ,192 | 2,527 | **,012** | ,918 | 7,483 | ,973 | 1,028 |
|  | Diazepam ED | ,811 | ,959 | ,065 | ,845 | ,399 | -1,083 | 2,704 | ,942 | 1,062 |
| *Notes.* R^2^ = 0.08 | | | | | | | | | | |

**Table S6.** Model 1 for BPSD-SINDEM coping.

|  | | | | | | | | | | |
| --- | --- | --- | --- | --- | --- | --- | --- | --- | --- | --- |
| Model | | **Unstandardized coefficient** | | **Standardized coefficient** | **t** | **p.** | **95,0% CI** | | **Collinearity** | |
|  |  | **B** | **Standard error** | **Beta** |  |  | **Lower limit** | **Upper limit** | **Tolerance** | **VIF** |
| 1 | Intercept | 63,010 | 19,137 |  | 3,293 | ,001 | 25,253 | 100,766 |  |  |
|  | Diagnosis=Vasc | -1,960 | 7,113 | -,027 | -,276 | ,783 | -15,995 | 12,075 | ,543 | 1,841 |
|  | **Diagnosis=AD** | **-12,343** | 5,924 | -,278 | -2,083 | **,039** | -24,032 | -,654 | ,282 | 3,548 |
|  | **Diagnosis=LBD** | **-16,187** | 6,856 | -,277 | -2,361 | **,019** | -29,713 | -2,661 | ,364 | 2,744 |
|  | Diagnosis=FTD | -13,497 | 8,280 | -,155 | -1,630 | ,105 | -29,835 | 2,840 | ,554 | 1,805 |
|  | Diagnosis=other | -2,389 | 9,208 | -,022 | -,259 | ,796 | -20,557 | 15,779 | ,679 | 1,473 |
|  | Caregiver age | ,006 | ,152 | ,004 | ,040 | ,968 | -,293 | ,305 | ,657 | 1,522 |
|  | Caregiver education | ,060 | ,452 | ,011 | ,132 | ,895 | -,832 | ,952 | ,698 | 1,432 |
|  | Caregiver gender | 5,729 | 3,623 | ,116 | 1,581 | ,116 | -1,419 | 12,876 | ,935 | 1,070 |
|  | CDR | ,691 | 2,808 | ,031 | ,246 | ,806 | -4,850 | 6,231 | ,308 | 3,247 |
|  | MMSE raw | -,181 | ,332 | -,057 | -,543 | ,587 | -,836 | ,475 | ,461 | 2,168 |
|  | BPSD drugs | 2,368 | 3,554 | ,053 | ,666 | ,506 | -4,644 | 9,380 | ,804 | 1,244 |
|  | Other caregivers | -5,329 | 3,871 | -,115 | -1,377 | ,170 | -12,966 | 2,309 | ,721 | 1,387 |
|  | CIRS severity | 4,146 | 5,087 | ,068 | ,815 | ,416 | -5,890 | 14,182 | ,725 | 1,379 |
|  | ADL | -,523 | 1,126 | -,047 | -,465 | ,642 | -2,744 | 1,697 | ,496 | 2,015 |
|  | Primary caregiver | -2,863 | 4,271 | -,058 | -,670 | ,503 | -11,289 | 5,563 | ,680 | 1,470 |
|  | BPSD severity | ,014 | ,055 | ,020 | ,245 | ,807 | -,095 | ,122 | ,724 | 1,381 |
| *Notes.* R^2^ = 0.08 | | | | | | | | | | |

**Table S7.** Model 1.1 for BPSD-SINDEM coping.

|  | | | | | | | | | | |
| --- | --- | --- | --- | --- | --- | --- | --- | --- | --- | --- |
| Model | | **Unstandardized coefficient** | | **Standardized coefficient** | **t** | **p.** | **95,0% CI** | | **Collinearity** | |
|  |  | **B** | **Standard error** | **Beta** |  |  | **Lower limit** | **Upper limit** | **Tolerance** | **VIF** |
| 1 | Intercept | 65,921 | 18,893 |  | 3,489 | <,001 | 28,644 | 103,197 |  |  |
|  | Diagnosis=Vasc | -2,944 | 6,975 | -,040 | -,422 | ,673 | -16,706 | 10,818 | ,548 | 1,824 |
|  | **Diagnosis=AD** | **-11,543** | 5,776 | -,260 | -1,999 | **,047** | -22,938 | -,148 | ,288 | 3,473 |
|  | **Diagnosis=LBD** | **-16,446** | 6,713 | -,282 | -2,450 | **,015** | -29,691 | -3,202 | ,369 | 2,710 |
|  | Diagnosis=FTD | -14,117 | 8,163 | -,162 | -1,729 | ,085 | -30,222 | 1,988 | ,554 | 1,807 |
|  | Diagnosis=other | -1,519 | 9,049 | -,014 | -,168 | ,867 | -19,372 | 16,334 | ,682 | 1,466 |
|  | Caregiver age | -,021 | ,150 | -,012 | -,138 | ,891 | -,316 | ,275 | ,654 | 1,530 |
|  | Caregiver education | ,025 | ,446 | ,005 | ,055 | ,956 | -,855 | ,904 | ,697 | 1,434 |
|  | Caregiver gender | 6,513 | 3,585 | ,132 | 1,817 | ,071 | -,559 | 13,586 | ,927 | 1,079 |
|  | CDR | ,397 | 2,769 | ,018 | ,143 | ,886 | -5,066 | 5,860 | ,307 | 3,252 |
|  | MMSE raw | -,213 | ,327 | -,067 | -,650 | ,517 | -,859 | ,433 | ,461 | 2,169 |
|  | Other caregivers | -5,805 | 3,809 | -,125 | -1,524 | ,129 | -13,319 | 1,710 | ,723 | 1,383 |
|  | CIRS severity | 3,772 | 4,997 | ,062 | ,755 | ,451 | -6,087 | 13,631 | ,730 | 1,371 |
|  | ADL | -,628 | 1,110 | -,056 | -,566 | ,572 | -2,817 | 1,562 | ,496 | 2,018 |
|  | Primary caregiver | -3,126 | 4,199 | -,063 | -,745 | ,457 | -11,410 | 5,158 | ,683 | 1,464 |
|  | **Benzodiazepines** | **10,688** | 4,375 | ,177 | 2,443 | **,016** | 2,057 | 19,319 | ,934 | 1,071 |
|  | BPSD severity | ,022 | ,053 | ,033 | ,415 | ,679 | -,082 | ,125 | ,776 | 1,289 |
| *Notes.* R^2^ = 0.11 | | | | | | | | | | |

**Table S8.** Model 2 for BPSD-SINDEM coping.

|  | | | | | | | | | | |
| --- | --- | --- | --- | --- | --- | --- | --- | --- | --- | --- |
| **Model** | | **Unstandardized coefficient** | | **Standardized coefficient** | **t** | **p.** | **95,0% CI** | | **Collinearity** | |
|  |  | **B** | **Standard error** | **Beta** |  |  | **Lower limit** | **Upper limit** | **Tolerance** | **VIF** |
| 1 | Intercept | 54,081 | 2,191 |  | 24,686 | <,001 | 49,762 | 58,400 |  |  |
|  | Antipsychotics | -,763 | 3,321 | -,016 | -,230 | ,819 | -7,311 | 5,785 | ,990 | 1,010 |
|  | Antidepressants | -,494 | 3,587 | -,010 | -,138 | ,891 | -7,566 | 6,578 | ,986 | 1,014 |
|  | **Benzodiazepines** | **10,438** | 4,375 | ,167 | 2,386 | **,018** | 1,812 | 19,064 | ,979 | 1,022 |
| *Notes.* R^2^ = 0.03 | | | | | | | | | | |

**Table S9.** Model 3 for BPSD-SINDEM coping.

|  | | | | | | | | | | |
| --- | --- | --- | --- | --- | --- | --- | --- | --- | --- | --- |
| Model | | **Unstandardized coefficient** | | **Standardized coefficient** | **t** | **p.** | **95,0% CI** | | **Collinearity** | |
|  |  | **B** | **Standard error** | **Beta** |  |  | **Lower limit** | **Upper limit** | **Tolerance** | **VIF** |
| 1 | Intercept | 54,014 | 2,074 |  | 26,042 | <,001 | 49,919 | 58,110 |  |  |
|  | Fluoxetine ED | -,145 | ,175 | -,064 | -,827 | ,410 | -,490 | ,201 | ,968 | 1,033 |
|  | Olanzapine ED | 1,773 | 1,142 | ,120 | 1,552 | ,123 | -,482 | 4,028 | ,973 | 1,028 |
|  | Diazepam ED | ,977 | ,659 | ,117 | 1,482 | ,140 | -,324 | 2,278 | ,942 | 1,062 |
| *Notes.* R^2^ = 0.04 | | | | | | | | | | |

**Table S10.** Model 1 for BPSD-SINDEM observational.

|  | | | | | | | | | | |
| --- | --- | --- | --- | --- | --- | --- | --- | --- | --- | --- |
| Model | | **Unstandardized coefficient** | | **Standardized coefficient** | **t** | **p.** | **95,0% CI** | | **Collinearity** | |
|  |  | **B** | **Standard error** | **Beta** |  |  | **Lower limit** | **Upper limit** | **Tolerance** | **VIF** |
| 1 | Intercept | -11,542 | 13,498 |  | -,855 | ,394 | -38,169 | 15,085 |  |  |
|  | **RoB=Center** | **6,926** | 3,456 | ,154 | 2,004 | **,047** | ,108 | 13,744 | ,736 | 1,359 |
|  | RoB=South | 1,591 | 2,567 | ,044 | ,620 | ,536 | -3,472 | 6,654 | ,869 | 1,151 |
|  | Diagnosis=Vasc | 6,805 | 5,177 | ,124 | 1,314 | ,190 | -3,408 | 17,017 | ,490 | 2,041 |
|  | Diagnosis=AD | 5,278 | 4,316 | ,160 | 1,223 | ,223 | -3,237 | 13,793 | ,252 | 3,975 |
|  | **Diagnosis=LBD** | 8,442 | 4,697 | ,192 | 1,797 | ,074 | -,823 | 17,708 | ,379 | 2,636 |
|  | **Diagnosis=FTD** | 5,407 | 5,461 | ,086 | ,990 | ,323 | -5,365 | 16,179 | ,572 | 1,750 |
|  | Diagnosis=other | 6,322 | 6,301 | ,079 | 1,003 | ,317 | -6,108 | 18,753 | ,693 | 1,443 |
|  | Patient age | ,229 | ,163 | ,099 | 1,409 | ,161 | -,092 | ,550 | ,875 | 1,143 |
|  | Patient gender | ,297 | 2,346 | ,009 | ,126 | ,900 | -4,332 | 4,925 | ,866 | 1,155 |
|  | BPSD drugs | ,190 | 2,349 | ,006 | ,081 | ,936 | -4,444 | 4,825 | ,866 | 1,155 |
|  | Visit duration | -,075 | ,046 | -,125 | -1,629 | ,105 | -,167 | ,016 | ,734 | 1,363 |
|  | **CDR** | **4,738** | 1,647 | ,290 | 2,877 | **,004** | 1,490 | 7,986 | ,426 | 2,346 |
|  | MMSE raw | ,006 | ,232 | ,002 | ,025 | ,980 | -,451 | ,463 | ,442 | 2,264 |
| *Notes.* R^2^ = 0.19 | | | | | | | | | | |

| **Table S11.** Model 1.1 for BPSD-SINDEM observational. | | | | | | | | | | |
| --- | --- | --- | --- | --- | --- | --- | --- | --- | --- | --- |
| **Model** | | **Unstandardized coefficient** | | **Standardized coefficient** | **t** | **p.** | **95,0% CI** | | **Collinearity** | |
|  |  | **B** | **Standard error** | **Beta** |  |  | **Lower limit** | **Upper limit** | **Tolerance** | **VIF** |
| 1 | Intercept | -13,054 | 13,372 |  | -,976 | ,330 | -39,431 | 13,324 |  |  |
|  | **RoB=Center** | **7,332** | 3,405 | ,163 | 2,154 | **,033** | ,616 | 14,048 | ,744 | 1,344 |
|  | RoB=South | 1,819 | 2,544 | ,050 | ,715 | ,475 | -3,199 | 6,837 | ,868 | 1,152 |
|  | Diagnosis=Vasc | 6,071 | 5,077 | ,110 | 1,196 | ,233 | -3,944 | 16,086 | ,500 | 2,000 |
|  | Diagnosis=AD | 4,584 | 4,228 | ,139 | 1,084 | ,280 | -3,756 | 12,924 | ,257 | 3,887 |
|  | Diagnosis=LBD | 6,684 | 4,663 | ,152 | 1,433 | ,153 | -2,514 | 15,883 | ,378 | 2,648 |
|  | Diagnosis=FTD | 4,690 | 5,406 | ,075 | ,868 | ,387 | -5,975 | 15,355 | ,572 | 1,748 |
|  | Diagnosis=other | 6,665 | 6,230 | ,084 | 1,070 | ,286 | -5,625 | 18,956 | ,696 | 1,438 |
|  | Patient age | ,237 | ,161 | ,103 | 1,473 | ,142 | -,080 | ,555 | ,876 | 1,142 |
|  | Patient gender | ,561 | 2,328 | ,017 | ,241 | ,810 | -4,031 | 5,154 | ,863 | 1,159 |
|  | Antipsychotics | 5,207 | 2,736 | ,138 | 1,903 | ,059 | -,191 | 10,604 | ,803 | 1,245 |
|  | Visit duration | -,069 | ,046 | -,114 | -1,496 | ,136 | -,159 | ,022 | ,731 | 1,368 |
|  | **CDR** | **4,228** | 1,639 | ,259 | 2,579 | **,011** | ,994 | 7,461 | ,422 | 2,370 |
|  | MMSE raw | ,039 | ,230 | ,016 | ,168 | ,867 | -,414 | ,492 | ,441 | 2,267 |
| *Notes.* R^2^ = 0.20 | | | | | | | | | | |

**Table S12.** Model 2 for BPSD-SINDEM observational.

|  | | | | | | | | | | |
| --- | --- | --- | --- | --- | --- | --- | --- | --- | --- | --- |
| Model | | **Unstandardized coefficient** | | **Standardized coefficient** | **t** | **p.** | **95,0% CI** | | **Collinearity** | |
|  |  | **B** | **Standard error** | **Beta** |  |  | **Lower limit** | **Upper limit** | **Tolerance** | **VIF** |
| 1 | Intercept | 15,080 | 1,543 |  | 9,775 | <,001 | 12,038 | 18,121 |  |  |
|  | Antipsychotics | -,148 | 2,339 | -,004 | -,063 | ,950 | -4,759 | 4,463 | ,990 | 1,010 |
|  | **Antidepressants** | **10,161** | 2,526 | ,273 | 4,023 | **<,001** | 5,180 | 15,141 | ,986 | 1,014 |
|  | Benzodiazepines | -2,997 | 3,081 | -,066 | -,973 | ,332 | -9,072 | 3,078 | ,979 | 1,022 |
| *Notes.* R^2^ = 0.08 | | | | | | | | | | |

**Table S13.** Model 3 for BPSD-SINDEM observational scale.

|  | | | | | | | | | | |
| --- | --- | --- | --- | --- | --- | --- | --- | --- | --- | --- |
| **Model** | | **Unstandardized coefficient** | | **Standardized coefficient** | **t** | **p.** | **95,0% CI** | | **Collinearity** | |
|  |  | **B** | **Standard error** | **Beta** |  |  | **Lower limit** | **Upper limit** | **Tolerance** | **VIF** |
| 1 | Intercept | 17,837 | 1,569 |  | 11,366 | <,001 | 14,739 | 20,936 |  |  |
|  | Fluoxetine ED | -,154 | ,132 | -,091 | -1,162 | ,247 | -,416 | ,108 | ,968 | 1,033 |
|  | Olanzapine ED | 1,683 | ,864 | ,151 | 1,947 | ,053 | -,024 | 3,389 | ,973 | 1,028 |
|  | Diazepam ED | -,068 | ,499 | -,011 | -,136 | ,892 | -1,053 | ,917 | ,942 | 1,062 |
| *Notes.* R^2^ = 0.03 | | | | | | | | | | |

**Table S14.** Summary statistics according to centers.

|  | | **Center** | | | | | | | | **Total** | | **p-value** |
| --- | --- | --- | --- | --- | --- | --- | --- | --- | --- | --- | --- | --- |
|  |  | **Don Gnocchi** | | **Monza** | | **Castellanza** | | **Rome** | |  |  |  |
|  |  | N | % | N | % | N | % | N | % | N | % |  |
| PwD gender | M | 20 | 38,5% | 31 | 47,7% | 16 | 39,0% | 24 | 48,0% | 91 | 43,8% | 0,628 |
|  | F | 32 | 61,5% | 34 | 52,3% | 25 | 61,0% | 26 | 52,0% | 117 | 56,3% |  |
| Caregiver gender | M | 17 | 32,7% | 13 | 20,0% | 13 | 31,7% | 16 | 32,7% | 59 | 28,5% | 0,377 |
|  | F | 35 | 67,3% | 52 | 80,0% | 28 | 68,3% | 33 | 67,3% | 148 | 71,5% |  |
| Relation | Son/daughter | 29 | 55,8% | 35 | 53,8% | 21 | 51,2% | 23 | 46,0% | 108 | 51,9% | 0,515 |
|  | Partner | 21 | 40,4% | 24 | 36,9% | 17 | 41,5% | 26 | 52,0% | 88 | 42,3% |  |
|  | Other | 2 | 3,8% | 6 | 9,2% | 3 | 7,3% | 1 | 2,0% | 12 | 5,8% |  |
| Cohabitation | No | 25 | 48,1% | 36 | 55,4% | 22 | 53,7% | 12 | 24,0% | 95 | 45,7% | 0,004 |
|  | Yes | 27 | 51,9% | 29 | 44,6% | 19 | 46,3% | 38 | 76,0% | 113 | 54,3% |  |
| Primary caregiver | No | 13 | 25,0% | 25 | 38,5% | 10 | 24,4% | 8 | 16,0% | 56 | 26,9% | 0,054 |
|  | Yes | 39 | 75,0% | 40 | 61,5% | 31 | 75,6% | 42 | 84,0% | 152 | 73,1% |  |
| Other caregiver | No | 16 | 30,8% | 25 | 38,5% | 15 | 36,6% | 19 | 38,0% | 75 | 36,1% | 0,830 |
|  | Yes | 36 | 69,2% | 40 | 61,5% | 26 | 63,4% | 31 | 62,0% | 133 | 63,9% |  |
| Living situation | Home | 44 | 84,6% | 64 | 98,5% | 41 | 100,0% | 48 | 96,0% | 197 | 94,7% | 0,002 |
|  | Daycare | 8 | 15,4% | 1 | 1,5% | 0 | 0,0% | 2 | 4,0% | 11 | 5,3% |  |

*Notes.* Chi-square test.

**Table S15.** Differences in continuous variables across centers.

|  | **Center** | | | |  |  |
| --- | --- | --- | --- | --- | --- | --- |
|  | **Don Gnocchi** | **Monza** | **Castellanza** | **Rome** | **p values** | **Significant differences** |
| PwD age | 80.6 ± 6.2 | 75.2 ± 6.5 | 74.7 ± 8.5 | 77.0 ± 6.3 | < 0.001 | Don Gnocchi > others |
| PwD education | 7.7 ± 3.1 | 8.8 ± 3.9 | 7.8 ± 3.1 | 10.2 ± 4.2 | 0.004 | Rome > Don Gnocchi  Rome > Castellanza |
| Caregiver age | 63.2 ± 12.3 | 57.1 ± 13.0 | 57.9 ± 12.5 | 62.5 ± 12.9 | 0.021 | Don Gnocchi > Monza |
| Caregiver education | 12.4 ± 4.3 | 13.5 ± 4.0 | 11.2 ± 3.7 | 13.4 ± 4.6 | 0.023 | Monza > Castellanza |
| CDR | 2.3 ± 1.0 | 1.24 ± 1.0 | 1.37 ± 0.8 | 1.68 ± 0.8 | < 0.001 | Don Gnocchi > others |
| MMSE raw | 15.5 ± 7.6 | 19.2 ± 6.9 | 20.9 ± 4.8 | 18.7 ± 7.0 | < 0.001 | Castellanza > Don Gnocchi |
| MMSE adjusted | 15.5 ± 7.9 | 18.4 ± 6.7 | 20.2 ± 4.8 | 19.3 ± 11.6 | 0.030 | Castellanza > Don Gnocchi |
| ADL | 3.2 ± 1.8 | 4.8 ± 1.8 | 4.5 ± 1.7 | 3.8 ± 2.1 | < 0.001 | Don Gnocchi < Castellanza  Don Gnocchi < Monza |
| IADL | 1.4 ± 1.8 | 4.9 ± 3.1 | 3.7 ± 2.8 | 2.9 ± 2.6 | <0.001 | Don Gnocchi < others  Monza > Rome |
| CIRS severity | 1.8 ± 0.3 | 1.1 ± 0.2 | 1.3 ± 0.3 | 1.3 ± 0.3 | <0.001 | Don Gnocchi > others  Monza < others |
| CIRS comorbidity | 3.8 ± 1.9 | 0.4 ± 0.9 | 1.2 ± 1.3 | 0.7 ± 0.9 | <0.001 | Don Gnocchi > others  Monza < Castellanza |
| NPI frequency * severity | 42.7 ± 16.8 | 30.3 ± 24.0 | 20.8 ± 14.9 | 36.4 ± 21.2 | <0.001 | Don Gnocchi > Monza  Castellanza < others |
| NPI caregiver stress | 18.2 ± 7.8 | 14.7 ± 12.9 | 10.1 ± 7.1 | 18.3 ± 11.0 | < 0.001 | Don Gnocchi > Castellanza  Rome > Castellanza |

*Notes.* Data are reported as mean ± standard deviations. ANCOVA with post-hoc test.
