# Supplementary material for: Italian standardization of the BPSD-SINDEM scale for the assessment of neuropsychiatric symptoms in persons with dementia
Source: Front Neurol. 2024 Nov 21;15:1455787. doi: 10.3389/fneur.2024.1455787 (PMC11617322; doi:10.3389/fneur.2024.1455787)
Supplement: Supplementary file 2 [file Data_Sheet_1.docx]

**SCALA PER I DISTURBI PSICOCOMPORTAMENTALI DELLA SINDEM (SDPC-SINDEM)**

**QUESTIONARIO PER IL CAREGIVER**

Risponda alle domande riferendosi al comportamento nell’ultimo mese della persona di cui si prende cura. Le chiediamo di graduare il comportamento secondo due scale: 1) una scala di entità del comportamento (nel graduare l’entità prenda in considerazione sia la frequenza sia la gravità del comportamento) e 2) una scala relativa alle capacità che sente di avere nel gestire quel comportamento. La prima scala va da 0 (il comportamento non è presente) a 10 (il comportamento è il più intenso che lei possa immaginare). Apponga una crocetta fra 0 e 10 per graduare l’entità. Risponda anche nel caso sia presente uno solo dei comportamenti descritti. La seconda scala va da 0 (Lei non si sente per nulla in grado di gestire il comportamento) a 5 (Lei si sente perfettamente in grado di gestirlo). Apponga una crocetta fra 0 e 5 per graduare le Sue capacità di gestione. Le chiediamo inoltre di evidenziare, se possibile, in ogni domanda, le parole che descrivono i comportamenti del suo assistito *(fornire a chi risponde una matita con gommino, o, se non disponibile una penna o un evidenziatore).*

1. La persona di cui si occupa ha perso interesse per il mondo e le persone che la circondano, non prende più iniziative, deve essere sempre spinta a fare le cose? È indifferente rispetto alle emozioni altrui, ha scarse reazioni emotive?

*Entità del comportamento*


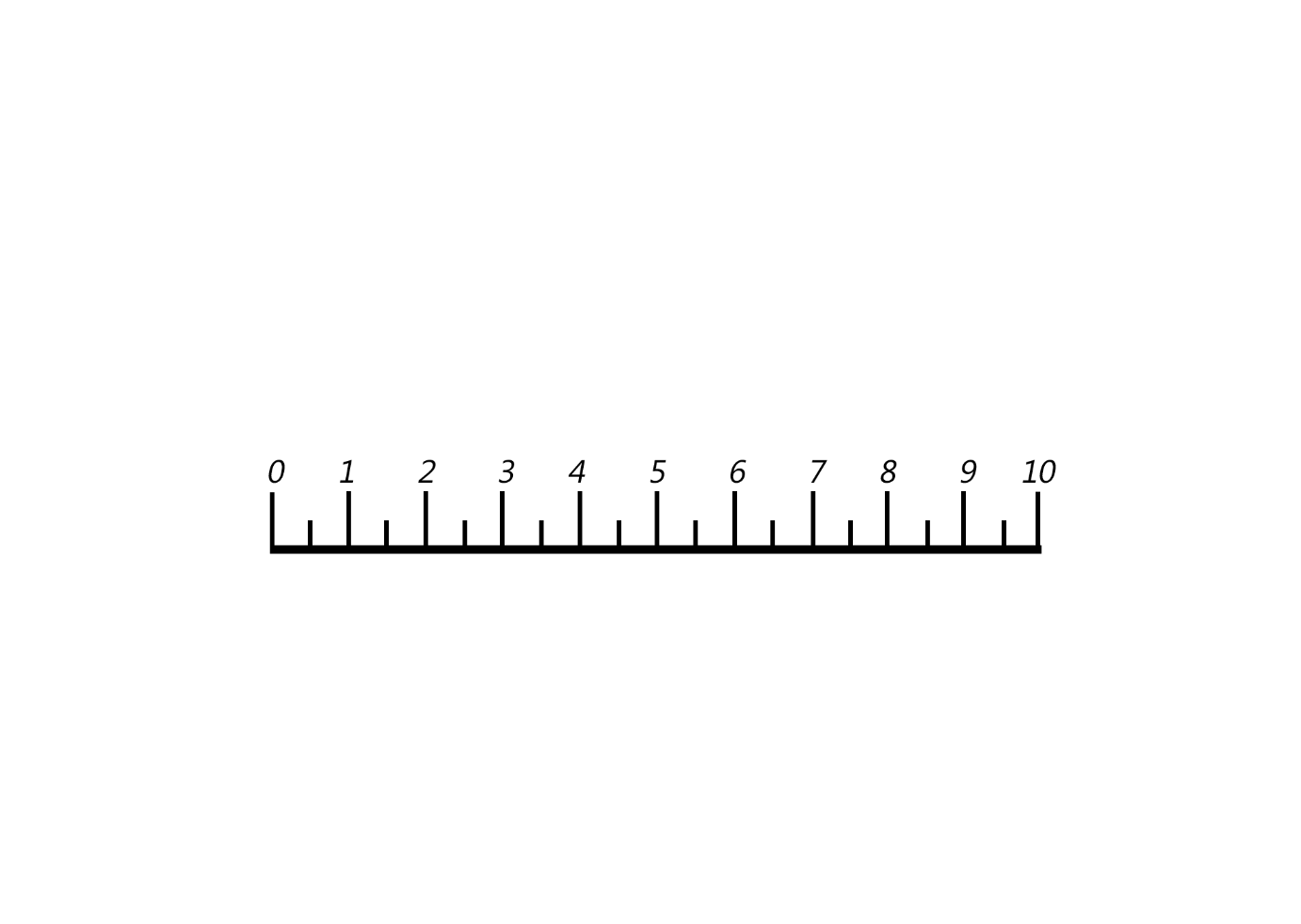


*Quanto mi sento in grado di gestire questo comportamento?*


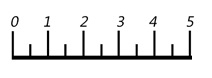


1. La persona di cui si occupa mostra segni di depressione, appare triste, piange facilmente, è molto lamentosa, riferisce disturbi fisici o malesseri senza apparente causa?

*Entità del comportamento*


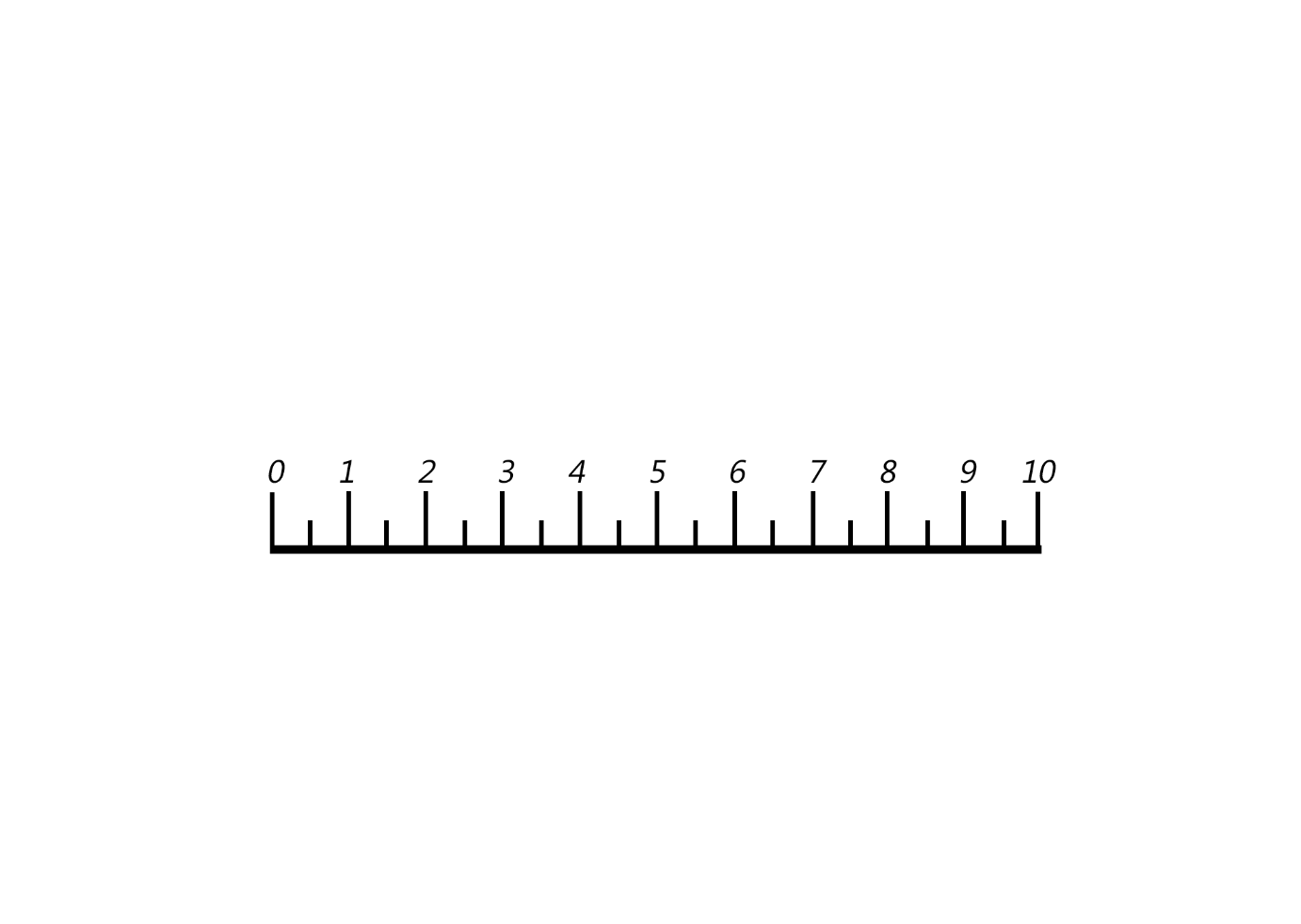


*Quanto mi sento in grado di gestire questo comportamento?*


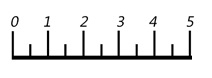


1. La persona di cui si occupa mostra sintomi d’ansia, anche solo legati a situazioni particolari (ad esempio essere lontana da casa, rimanere senza soldi, perdere la memoria o star male). Non tollera le attese; appare preoccupata di fronte alle novità? Vi segue come un’ombra?

*Entità del comportamento*


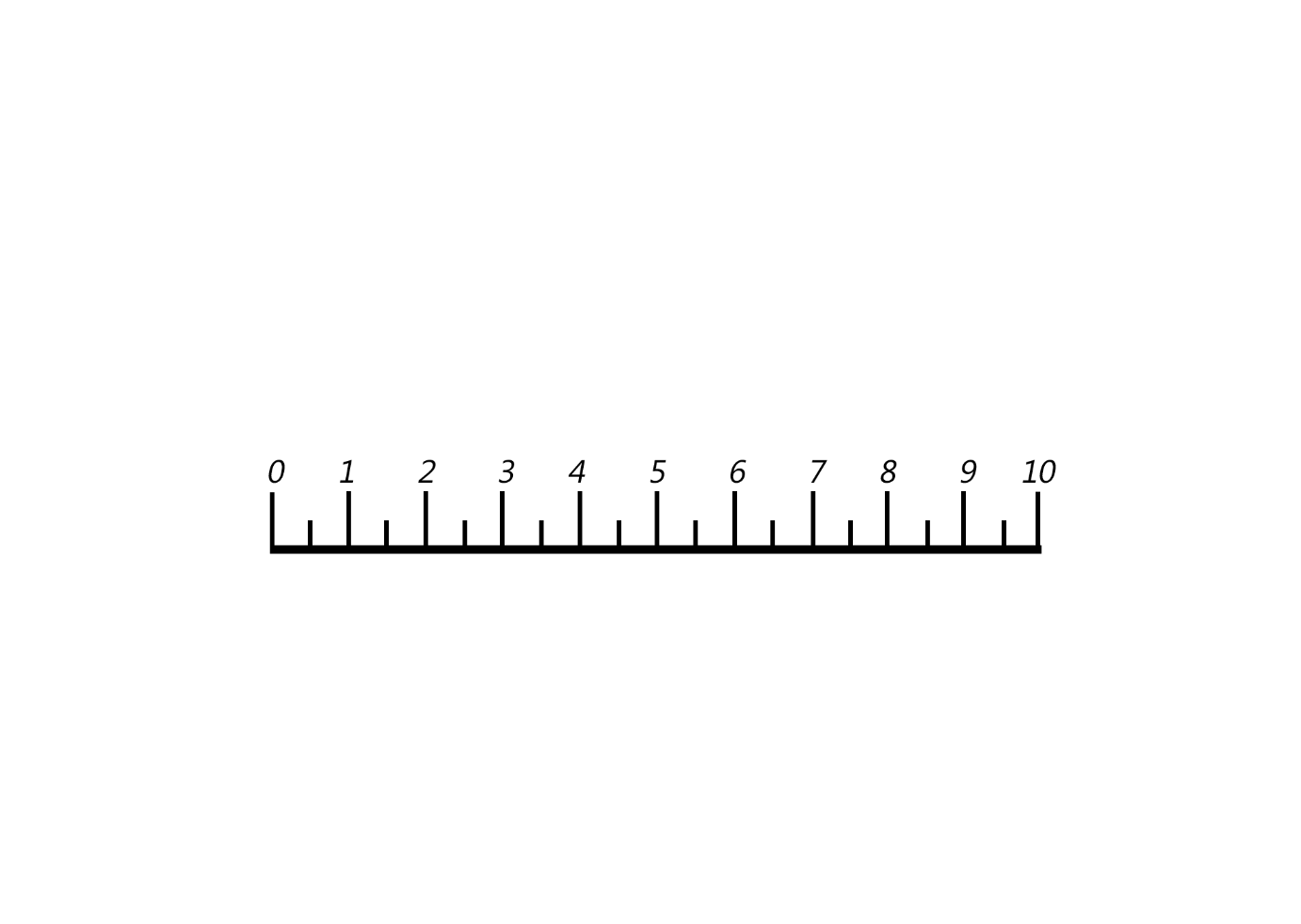


*Quanto mi sento in grado di gestire questo comportamento?*


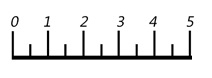


1. La persona di cui si occupa ha comportamenti ossessivi? Colleziona tutto ciò che trova? Mette in bocca ciò che trova in giro? Fa uso smodato di fumo o alcool?

*Entità del comportamento*


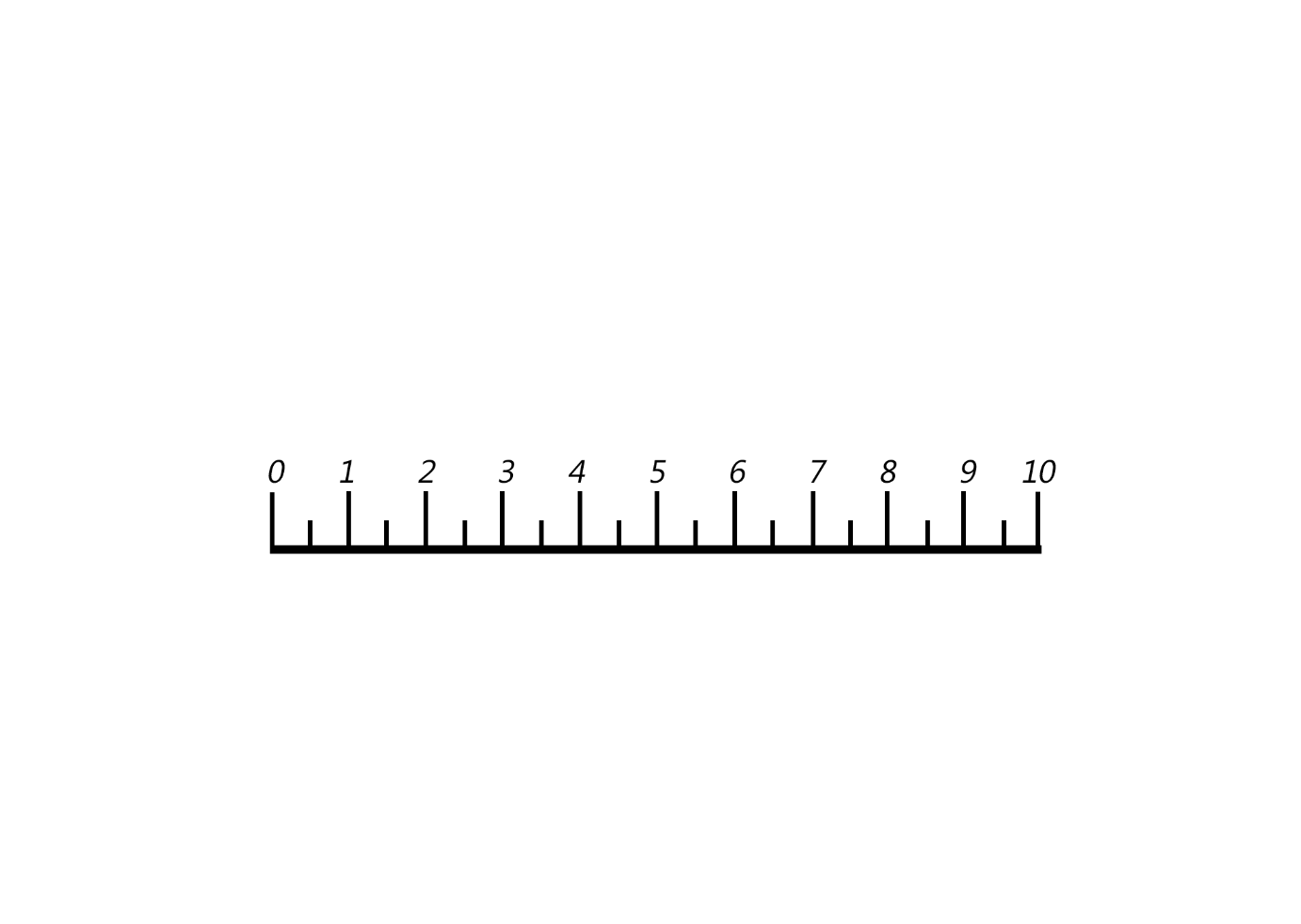


*Quanto mi sento in grado di gestire questo comportamento?*


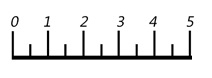


1. La persona di cui si occupa si mostra agitata, inquieta, cerca di richiamare in continuazione l’attenzione, gridando e lamentandosi? Cerca di scappare di casa o dal luogo ove viene assistita? Non riesce a stare seduta?

*Entità del comportamento*


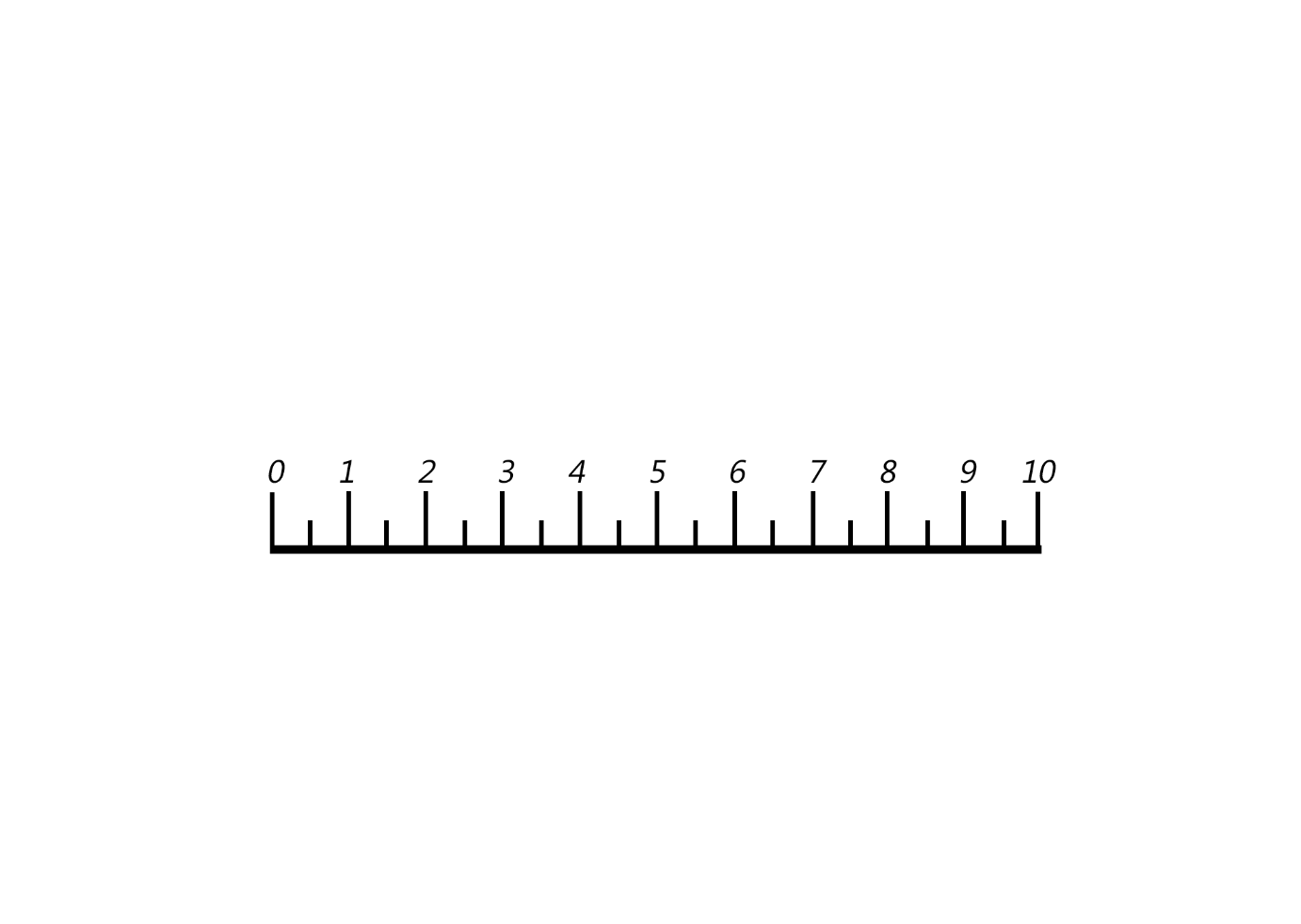


*Quanto mi sento in grado di gestire questo comportamento?*


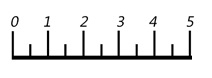


1. La persona di cui si occupa cammina per casa senza uno scopo preciso, rovistando fra gli oggetti o negli armadi, nascondendo effetti personali o soldi? Oppure ripete determinati gesti in continuazione? Continua a mettersi e togliersi i vestiti?

*Entità del comportamento*


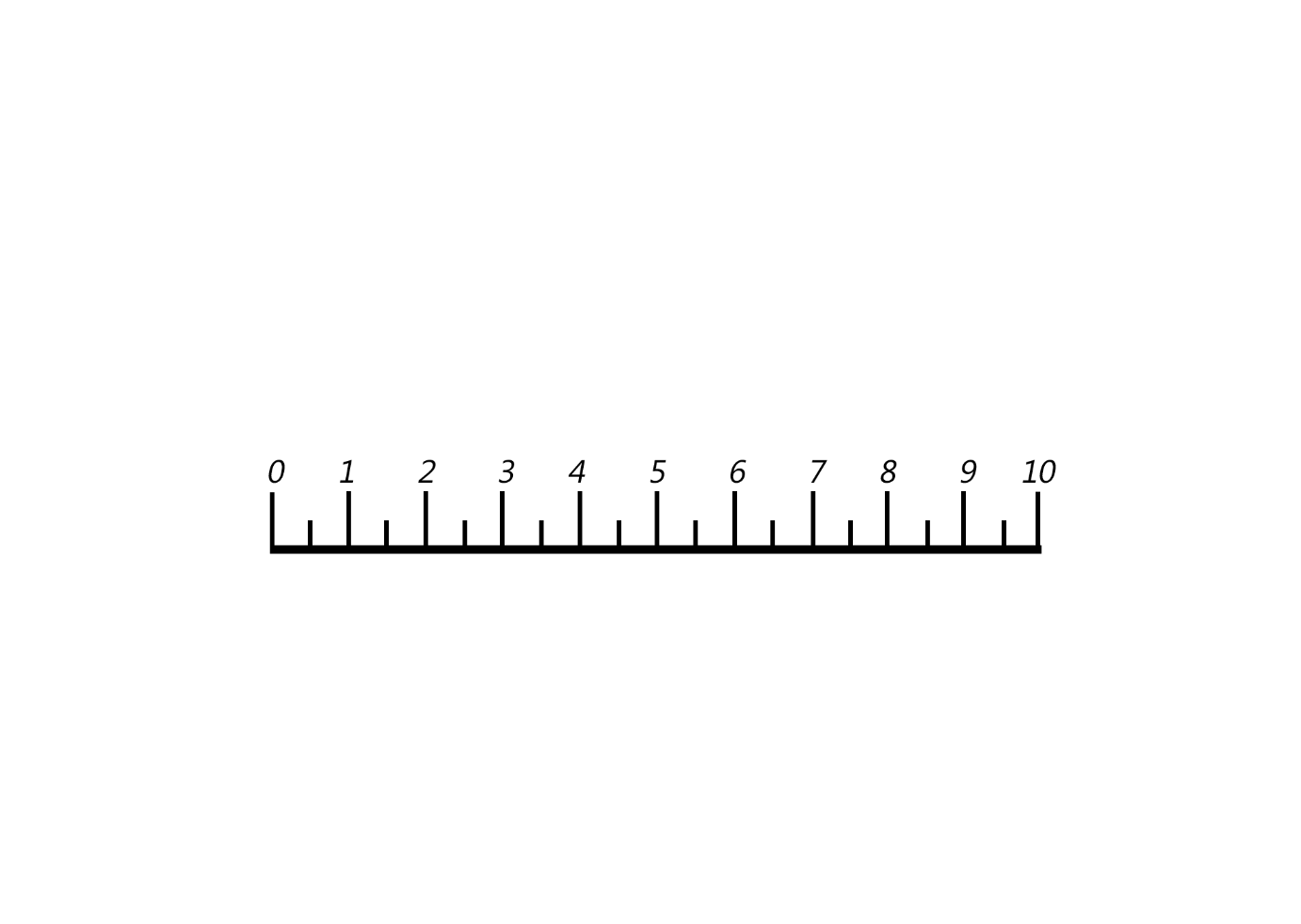


*Quanto mi sento in grado di gestire questo comportamento?*


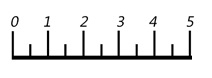


1. La persona di cui si occupa mostra aggressività verbale verso gli altri, insultando, alzando la voce o usando un tono aggressivo?

*Entità del comportamento*


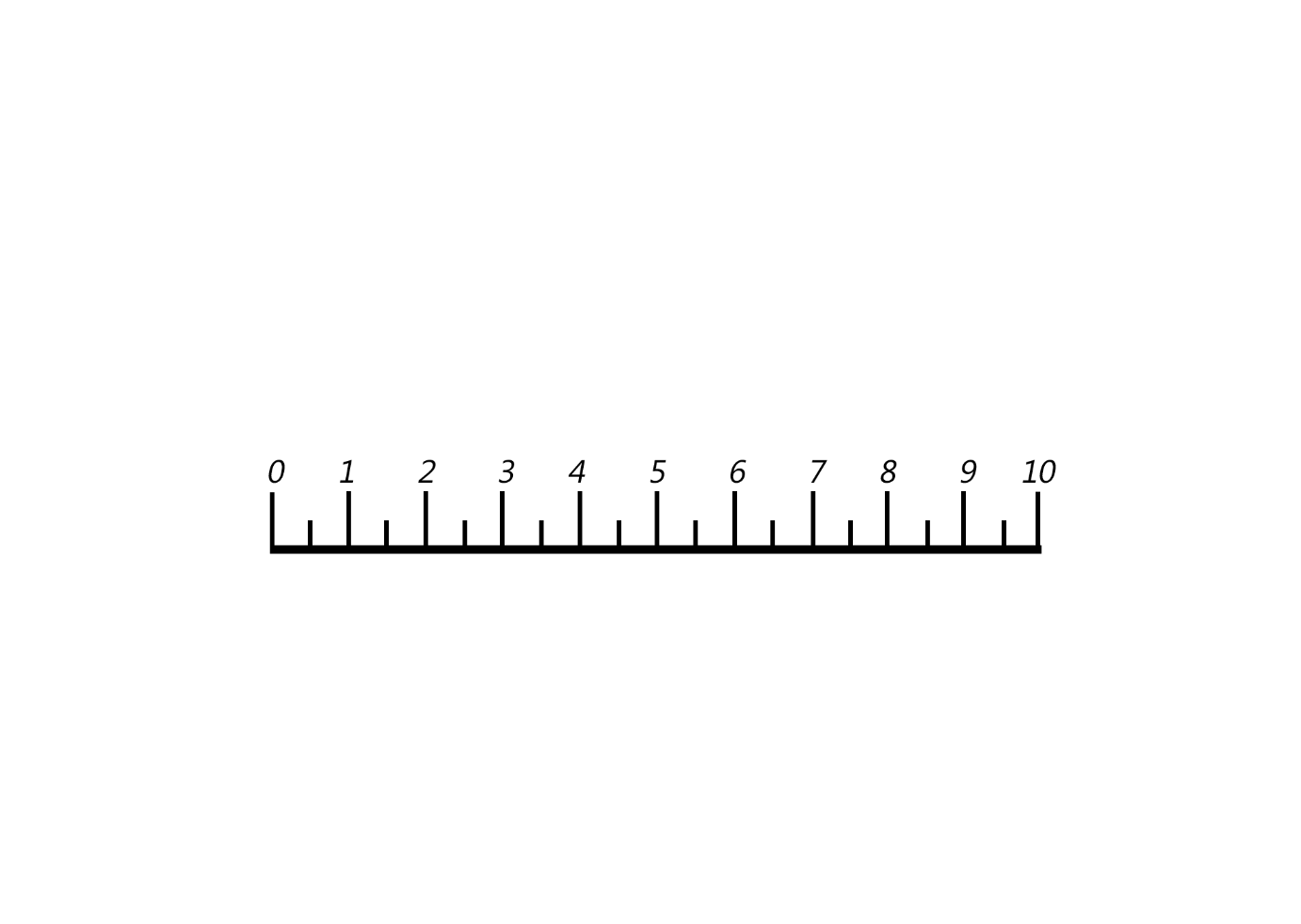


*Quanto mi sento in grado di gestire questo comportamento?*


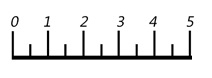


1. La persona di cui si occupa mostra aggressività fisica verso gli altri? Oppure distrugge, danneggia, strappa oggetti? Diventa aggressiva o oppositiva durante certe attività, come fare la doccia o vestirsi?

*Entità del comportamento*


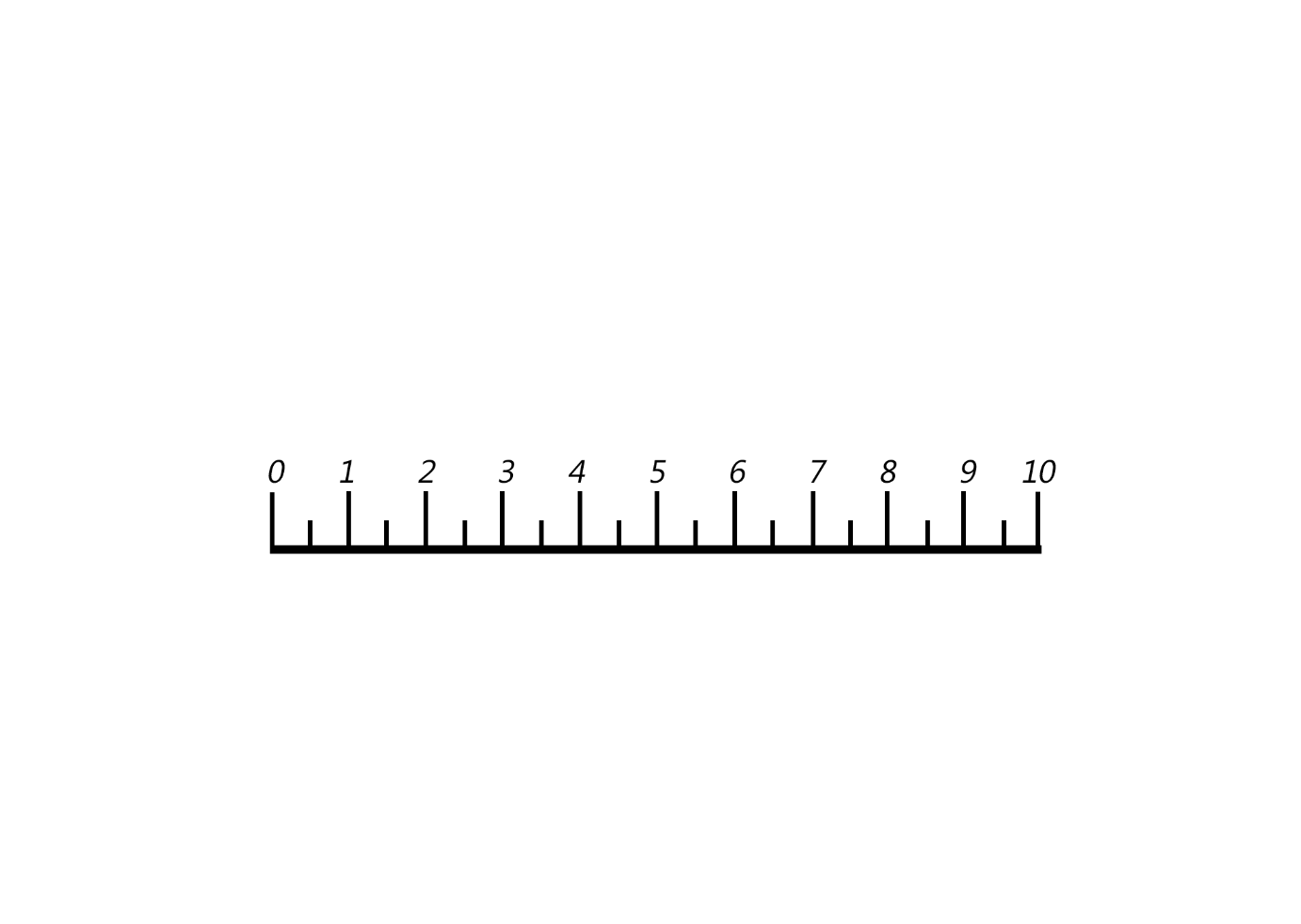


*Quanto mi sento in grado di gestire questo comportamento?*


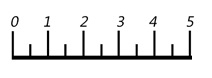


1. La persona di cui si occupa è facilmente irritabile, non accetta alcuna osservazione? Ha un umore molto variabile, manifesta improvvisi o ingiustificati scoppi d’ira?

*Entità del comportamento*


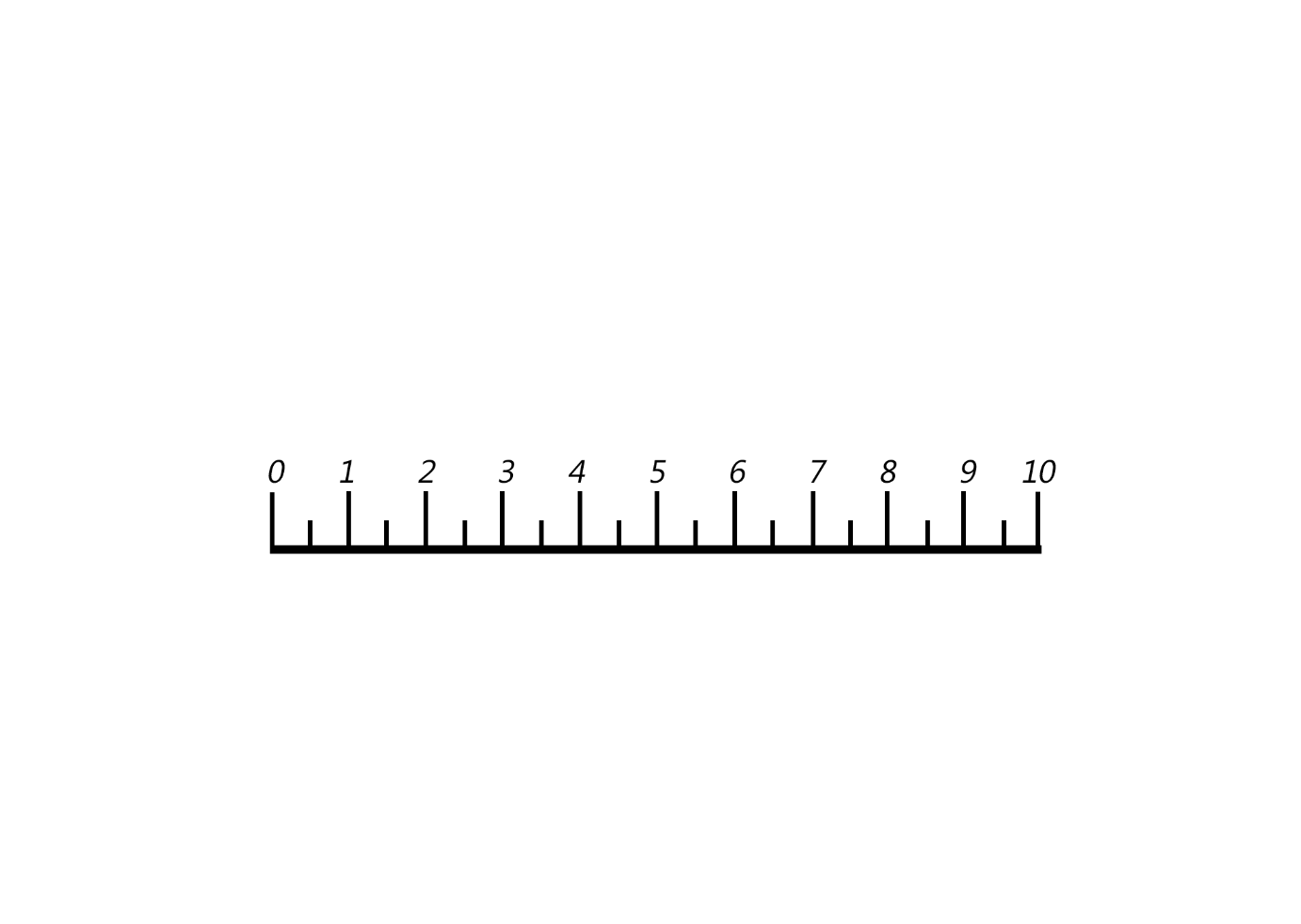


*Quanto mi sento in grado di gestire questo comportamento?*


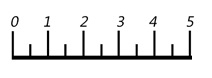


1. La persona di cui si occupa ha convinzioni deliranti, ad esempio pensa che qualcuno la stia derubando, oppure nasconda i suoi effetti personali; che il coniuge la tradisca; è convinta che i familiari vogliano abbandonarla o che qualcuno voglia farle del male o avvelenarla; Pensa che la casa non sia sua? Interagisce con i personaggi della TV convinta che siano persone reali o con la sua immagine riflessa nello specchio?

*Entità del comportamento*


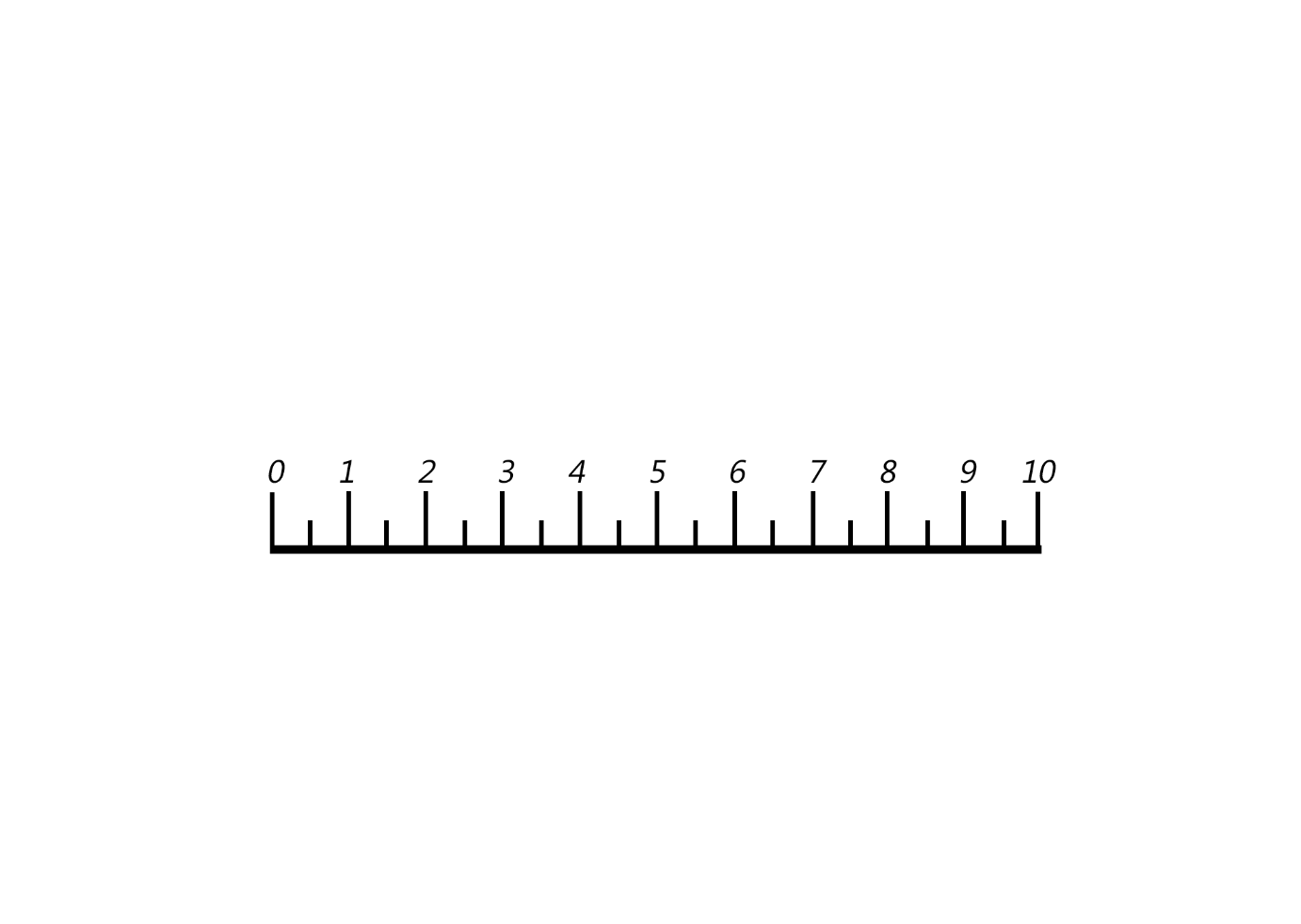


*Quanto mi sento in grado di gestire questo comportamento?*


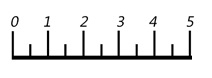


1. La persona di cui si occupa manifesta allucinazione, ossia vede persone, animali o cose che non esistono, o si comporta come se li vedesse? Sente rumori, suoni o musica inesistenti? Percepisce odori non reali? Soffre di prurito senza una logica spiegazione?

*Entità del comportamento*


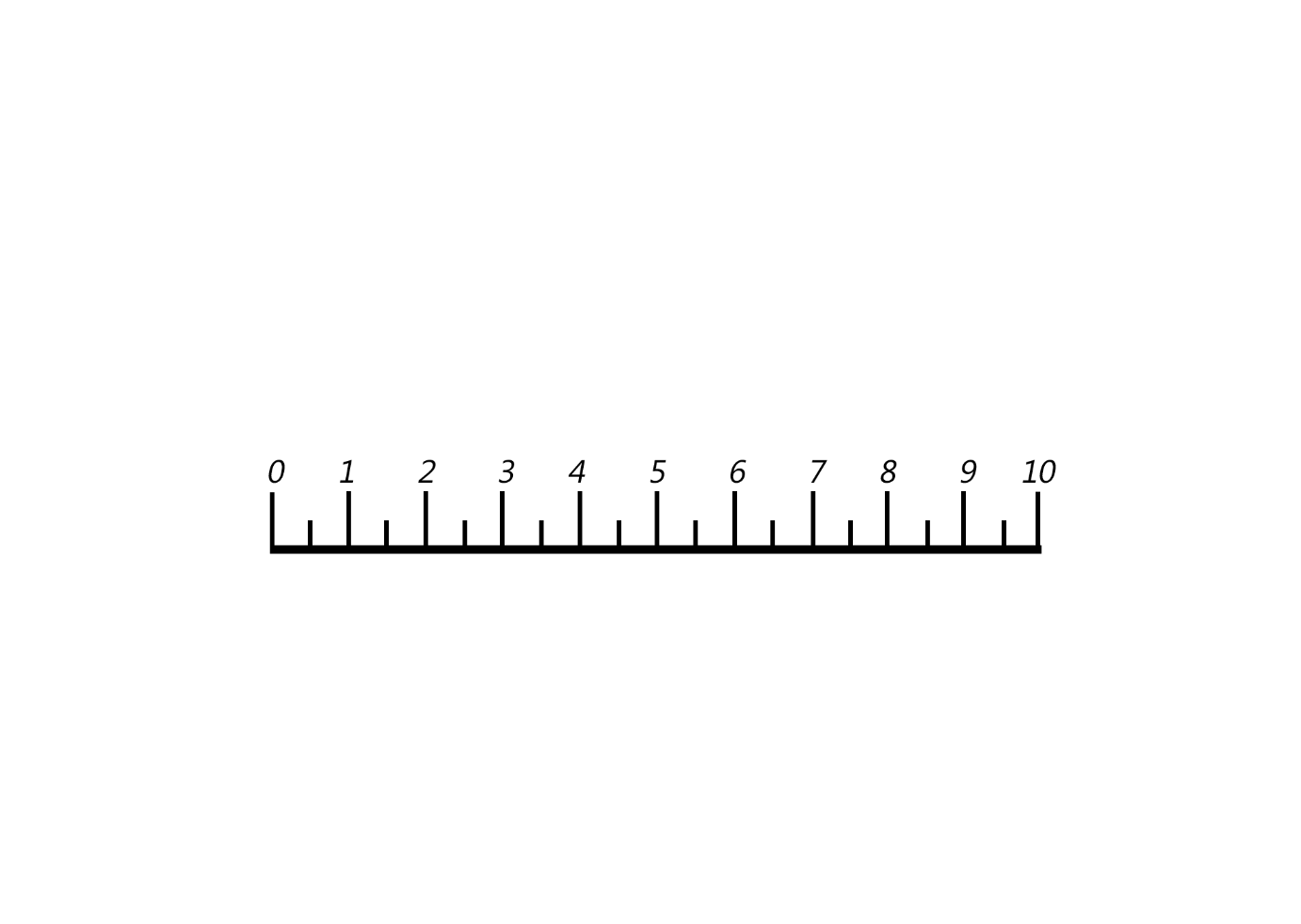


*Quanto mi sento in grado di gestire questo comportamento?*


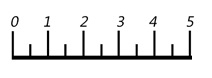


1. La persona di cui si occupa mostra eccessiva o inappropriata allegria di fronte agli avvenimenti della vita, fa scherzi stupidi, trova divertenti anche cose che in realtà non lo sono? Ride senza motivo?

*Entità del comportamento*


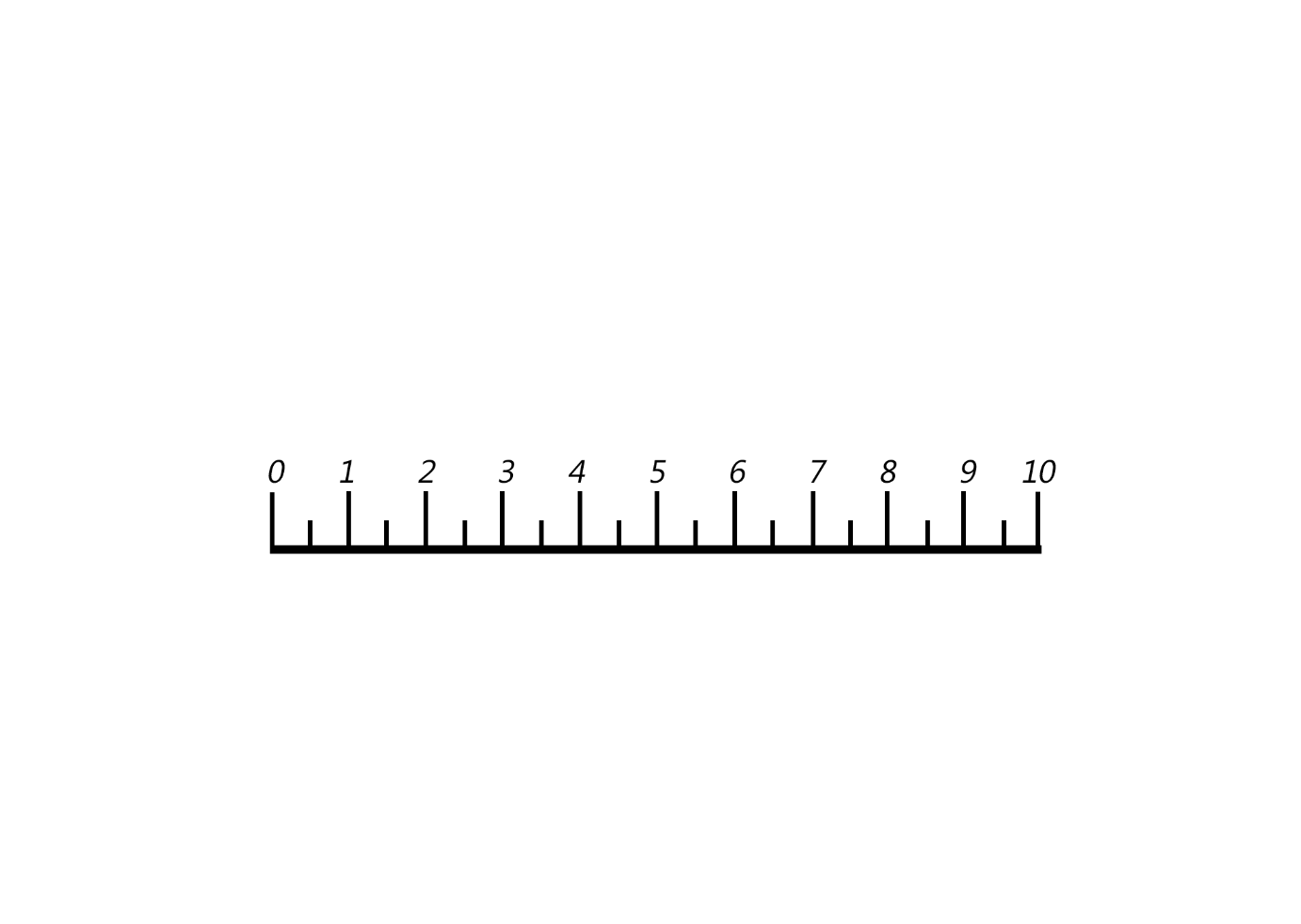


*Quanto mi sento in grado di gestire questo comportamento?*


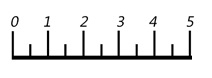


1. La persona di cui si occupa si comporta in maniera impulsiva? Fa cose imbarazzanti? Non rispetta le convenzioni sociali? Parla ad estranei come se li conoscesse? Fa avance sessuali, si spoglia in pubblico? Pretende insistentemente prestazioni sessuali dal partner? Parla in continuazione senza ascoltare le risposte?

*Entità del comportamento*


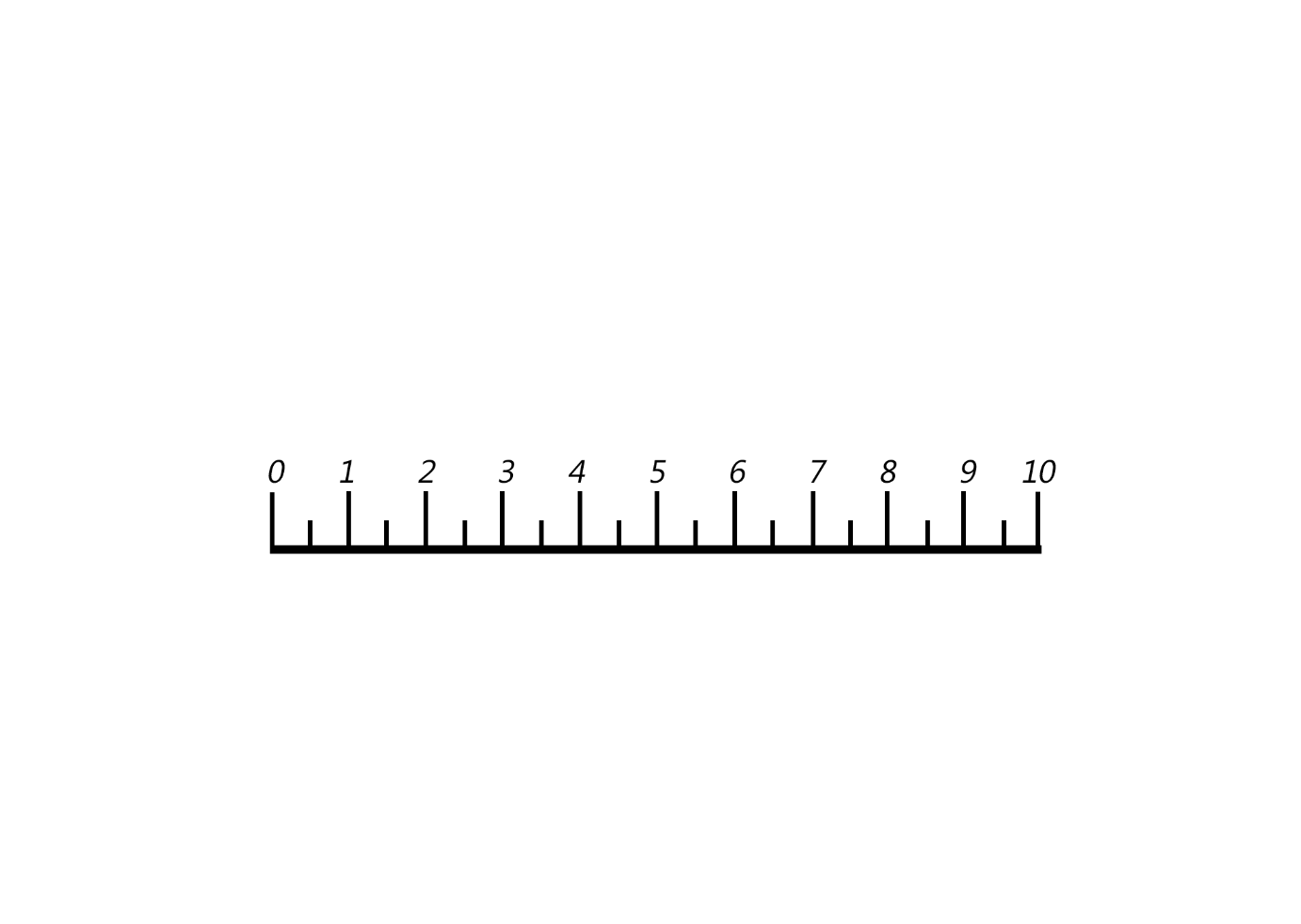


*Quanto mi sento in grado di gestire questo comportamento?*


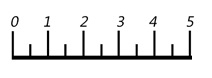


1. La persona di cui si occupa non riesce ad addormentarsi, si sveglia durante la notte e non riesce a riprendere sonno, vaga per la casa, cerca cibo nel frigo? Si sveglia molto presto al mattino? Pretende di uscire di casa a orari incongrui? Oppure è sonnolenta durante il giorno, fa molteplici riposini diurni? O ancora parla, grida, si agita, dà pugni o calci durante il sonno?

*Entità del comportamento*


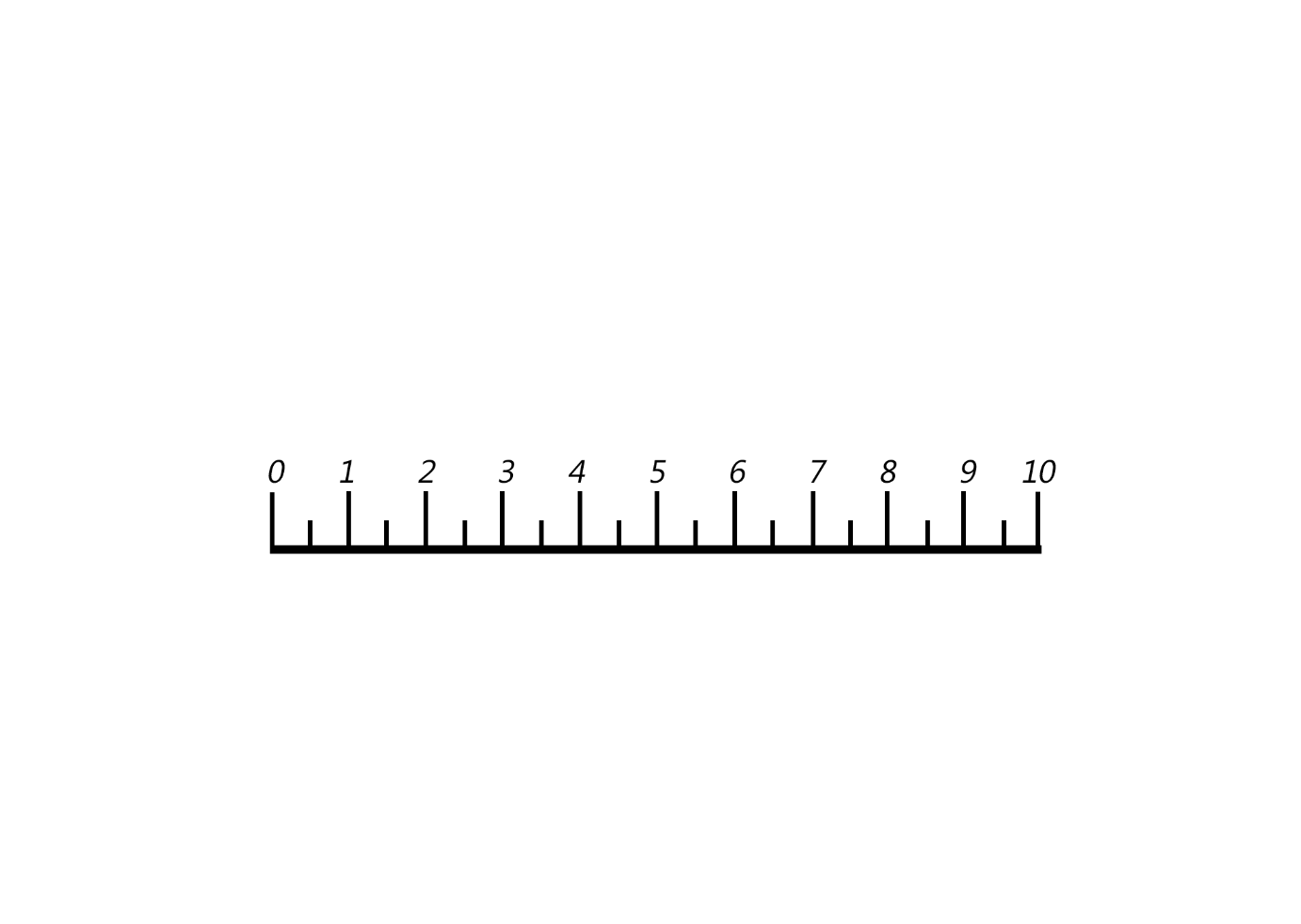


*Quanto mi sento in grado di gestire questo comportamento?*


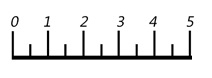


1. La persona di cui si occupa continua a ripetere le stesse domande decine di volte al giorno?

*Entità del comportamento*


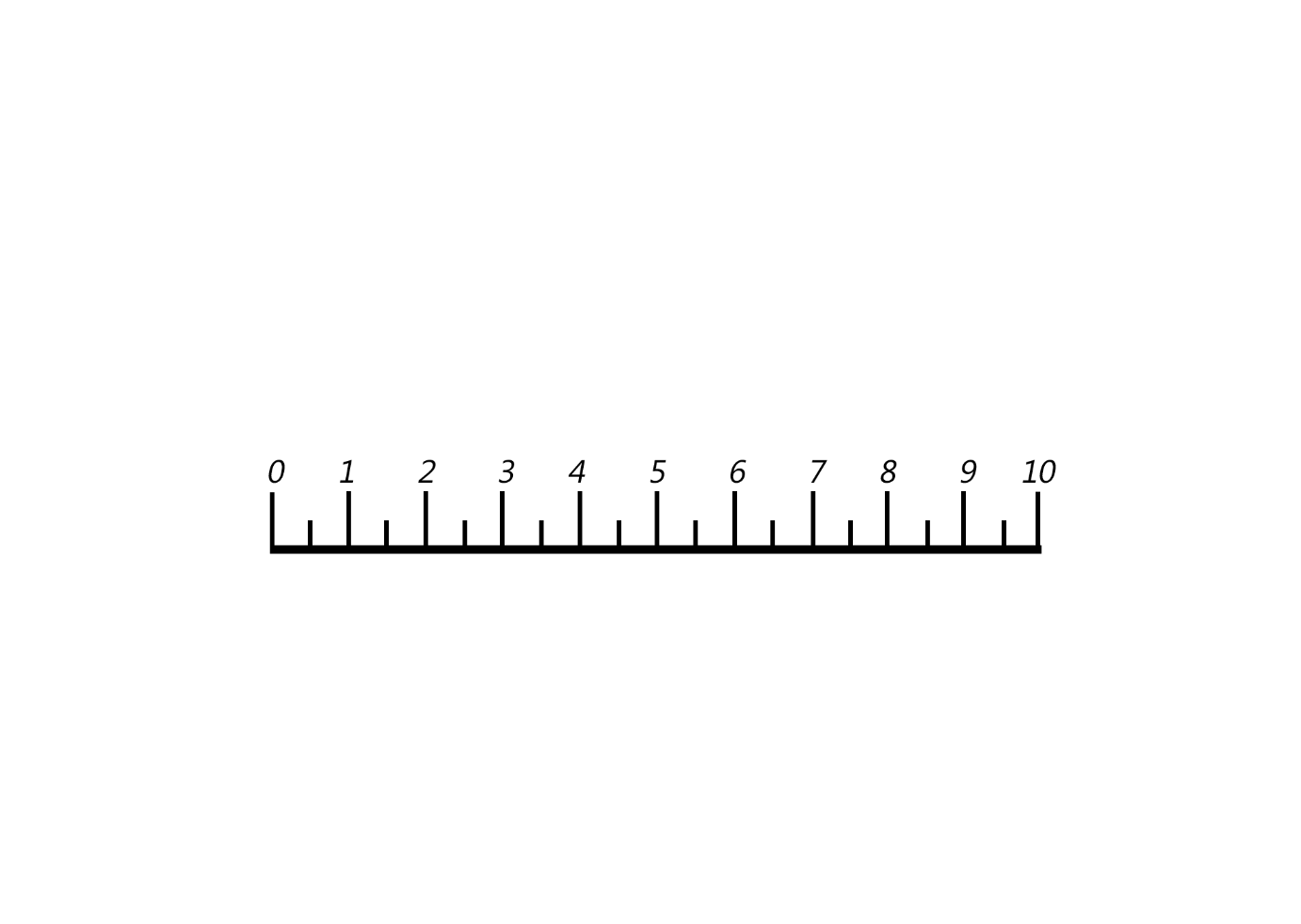


*Quanto mi sento in grado di gestire questo comportamento?*


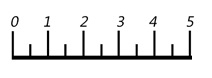


1. La persona di cui si occupa va stimolata per mangiare altrimenti si alimenterebbe in maniera insufficiente? Oppure al contrario mangerebbe in continuazione, vorrebbe sempre dolci e tende a prendere peso? Ha sviluppato preferenze alimentari bizzarre, mangia solo cibi molto selezionati? Rifiuta di bere in maniera adeguata?

*Entità del comportamento*


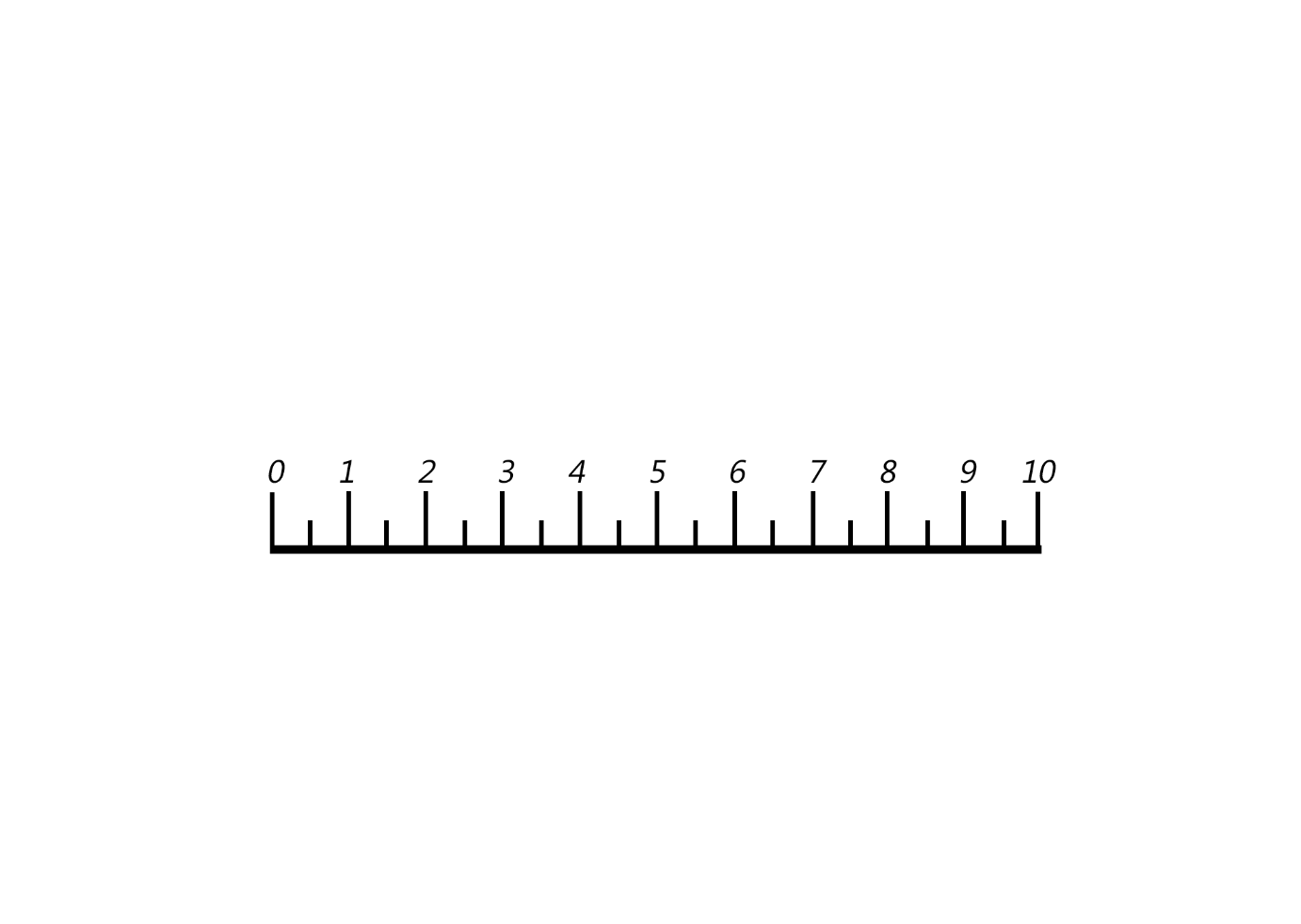


*Quanto mi sento in grado di gestire questo comportamento?*


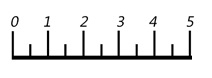


1. La persona di cui si occupa è più agitata e confusa nel pomeriggio e verso sera? Manifesta un aumento dei disturbi dell’umore e del comportamento quando scende il buio? Oppure questi disturbi variano a seconda della stagione o del tempo metereologico?

*Entità del comportamento*


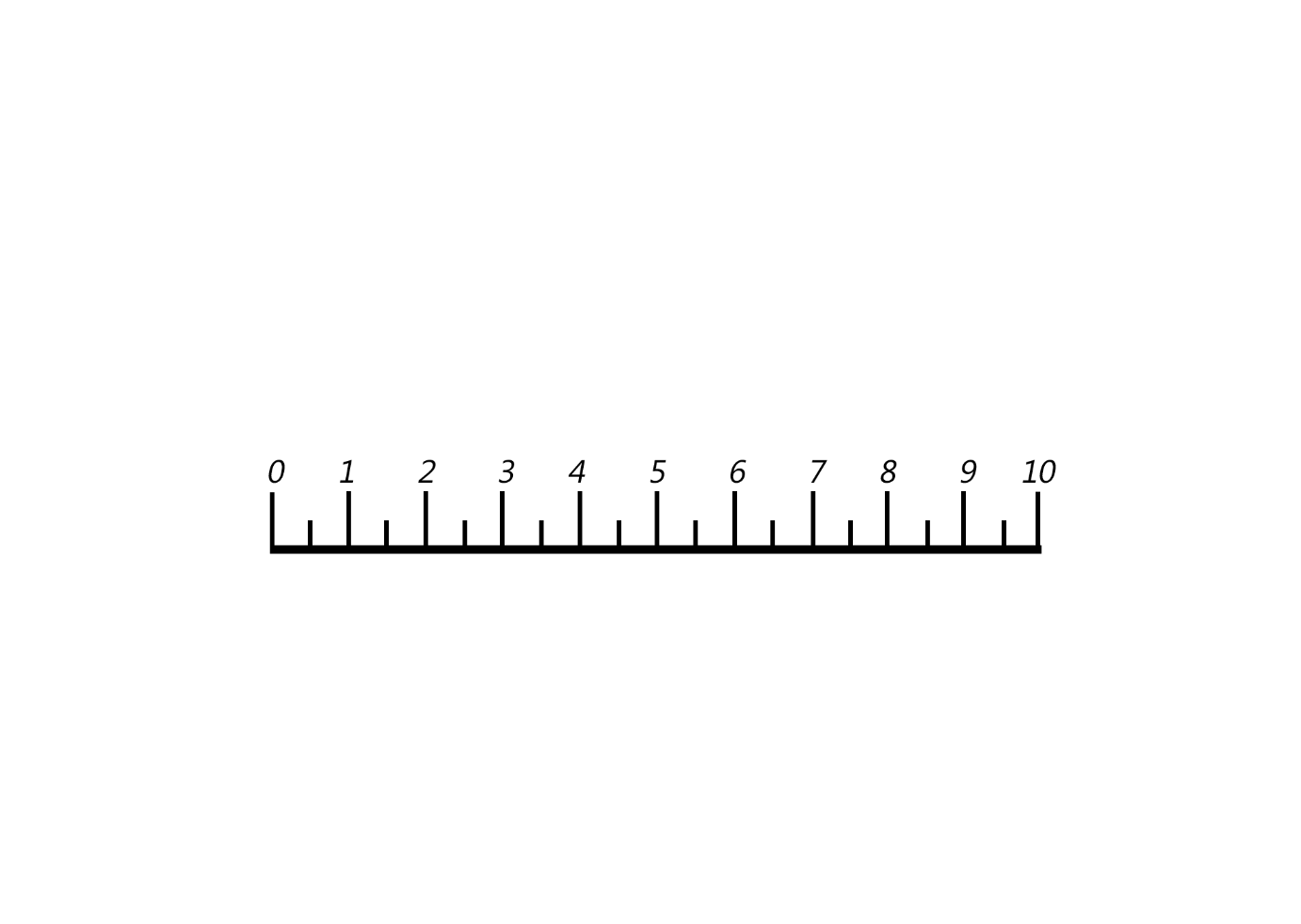


*Quanto mi sento in grado di gestire questo comportamento?*


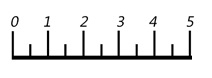


La ringraziamo per avere risposto alle nostre domande. Le sue risposte ci aiuteranno a prenderci cura nella maniera migliore della persona che assiste. Per favore risponda anche ad alcune domande che la riguardano:

Indichi la Sua Età____________Scolarità in anni____________e Sesso  M F Non dichiarato

Relazione con La persona che assiste:

- Coniuge/Compagno/a
- Figlio/Figlia
- Fratello/Sorella
- Altro parente
- Amico/Amica
- Caregiver professionale

E’ Lei il caregiver principale? Sì No

Ci sono altri caregiver Sì No

Se sì, indichi se gli altri caregiver sono familiari o professionisti

Convive con l’assistito? Sì No

Dove vive il Suo assistito? a domicilio

a domicilio ma frequenta un Centro Diurno

in una RSA

in un Villaggio Alzheimer
