# Supplementary material for: Italian standardization of the BPSD-SINDEM scale for the assessment of neuropsychiatric symptoms in persons with dementia
Source: Front Neurol. 2024 Nov 21;15:1455787. doi: 10.3389/fneur.2024.1455787 (PMC11617322; doi:10.3389/fneur.2024.1455787)
Supplement: Supplementary file 3 [file Data_Sheet_2.docx]

**SINDEM BEHAVIORAL AND PSYCHOLOGICAL SYMPTOMS DISORDER SCALE (BPSD-SINDEM)**

**CLINICIAN OBSERVATIONAL SCALE**

Answer the questions referring to *your direct observation of the behavior* of the person affected by dementia *during the visit/activity.* The aim is to describe the subject's behavior at that moment accurately. Behaviors that the person affected by dementia has exhibited at another moment should not be considered. Observed behaviors should be accurately grated according to a visual graduated scale that assesses the extent of the behavior itself, ranging from 0 (“the behavior is not present”) to 10 (“the extent of the behavior is the greatest you can imagine; the extent should be assessed based on the intensity and frequency of the behavior during the examination”). Place a cross between 0 and 10 to rate the extent of the behavior itself. We ask you to highlight, for the description of each behavior, the points that describe the observed behaviors (the respondent should use a pencil with an eraser - if not available, a pen - and a highlighter)."

**Apathy**

The person with dementia:

1. appears minimally interested in the visit/activity, the people present, and their surroundings; withdraws from the context and conversation;
2. doesn't take any verbal initiative and always needs to be prompted to do things;
3. appears emotionally blunted, indifferent to others' emotions, and has limited emotional reactions;

*Extent of behavior*


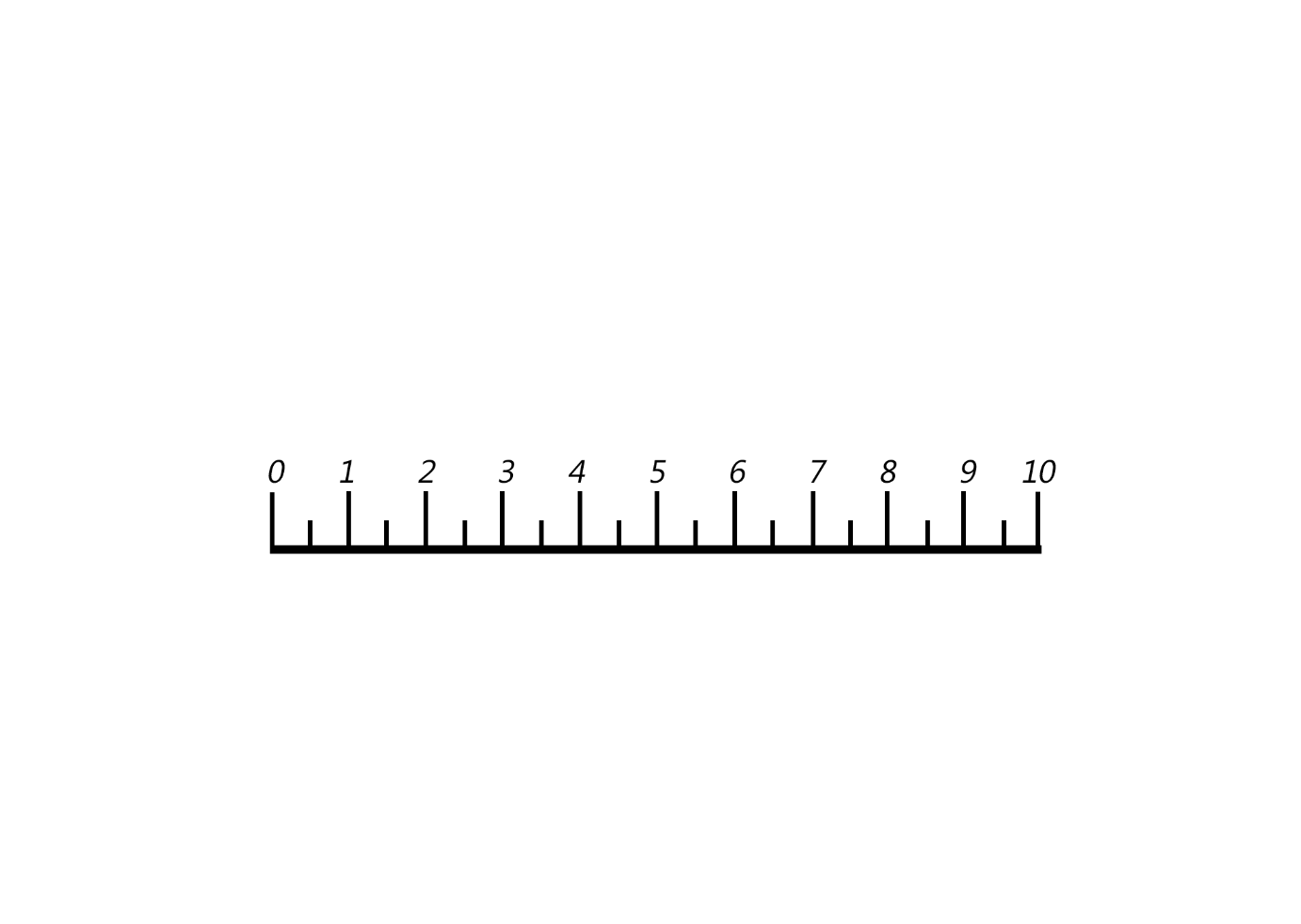


**Depression**

The person with dementia:

1. shows signs of depression, appears sad, discouraged, hopeless, and cries easily;
2. states that life is no longer worth living;
3. states that he/she is no longer himself/herself or that he/she is worthless;
4. complains of physical discomfort or disturbances that appear to have a psychological cause;

*Extent of behavior*


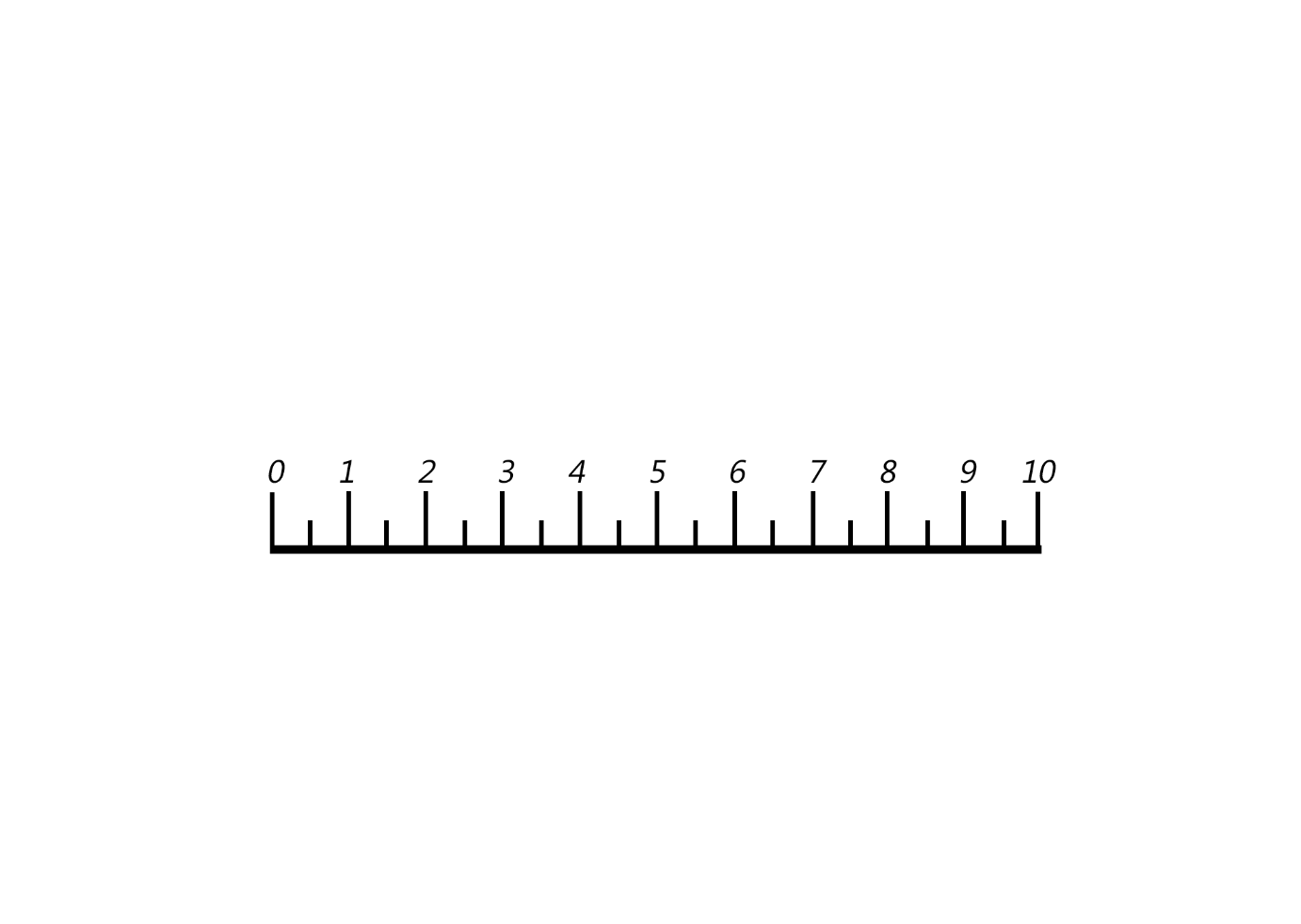


**Anxiety**

The person with dementia;

1. shows anxiety symptoms;
2. is concerned because s/he is far from home or has to stay away from the caregiver;
3. is afraid of running out of money;
4. is afraid of losing memory or his/her health;
5. cannot tolerate waiting;
6. appear tense or worried, especially when confronted with new things;
7. exhibits phobias;

*Extent of behavior*


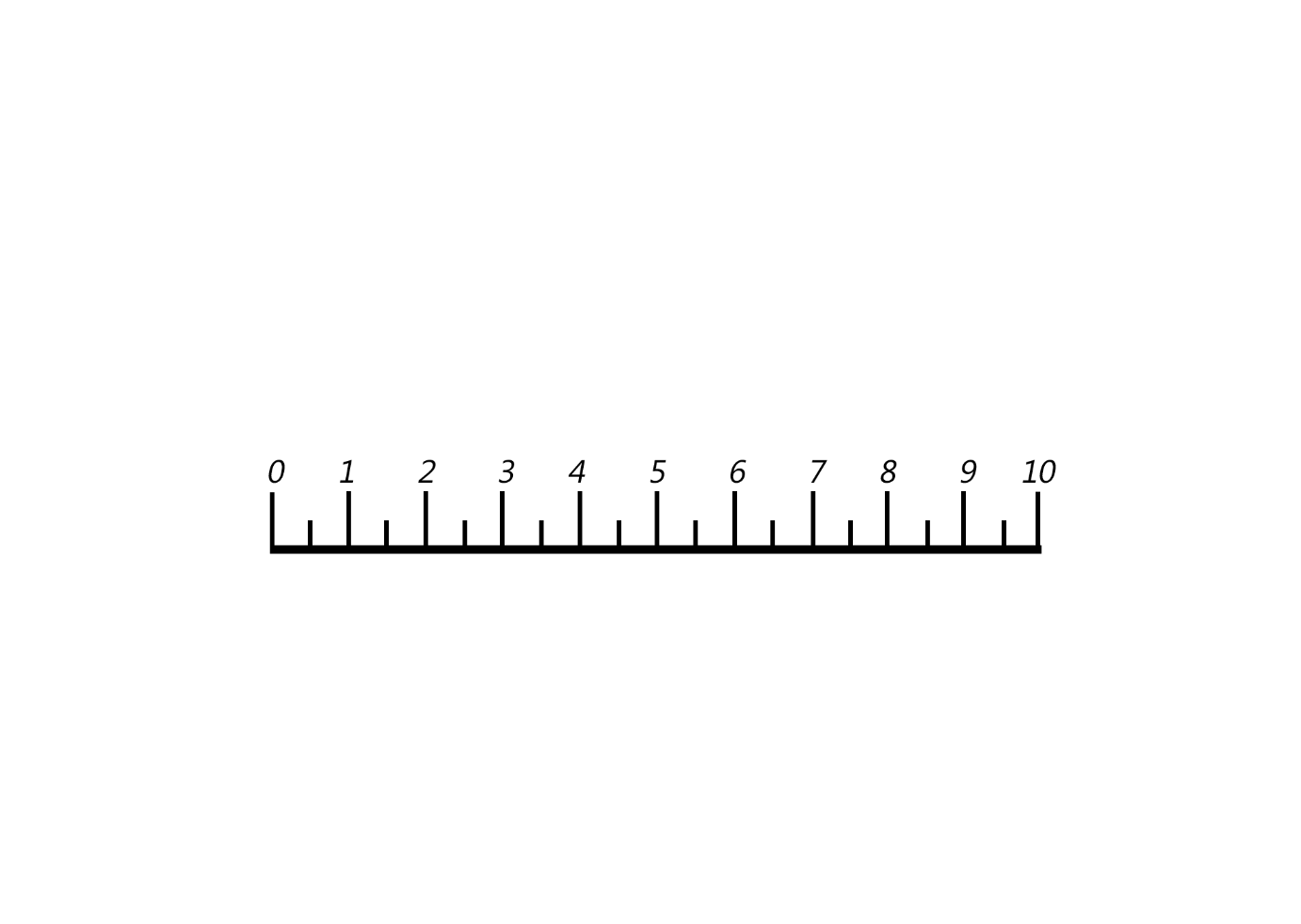


**Compulsion**

The person with dementia:

1. exhibits compulsive behaviors, such as repeatedly performing specific gestures or verbalizations or engaging in certain rituals;
2. collects or touches or puts things found around into his/her mouth;
3. obsessively asks for cigarettes or drinks/food;

.

*Extent of behavior*


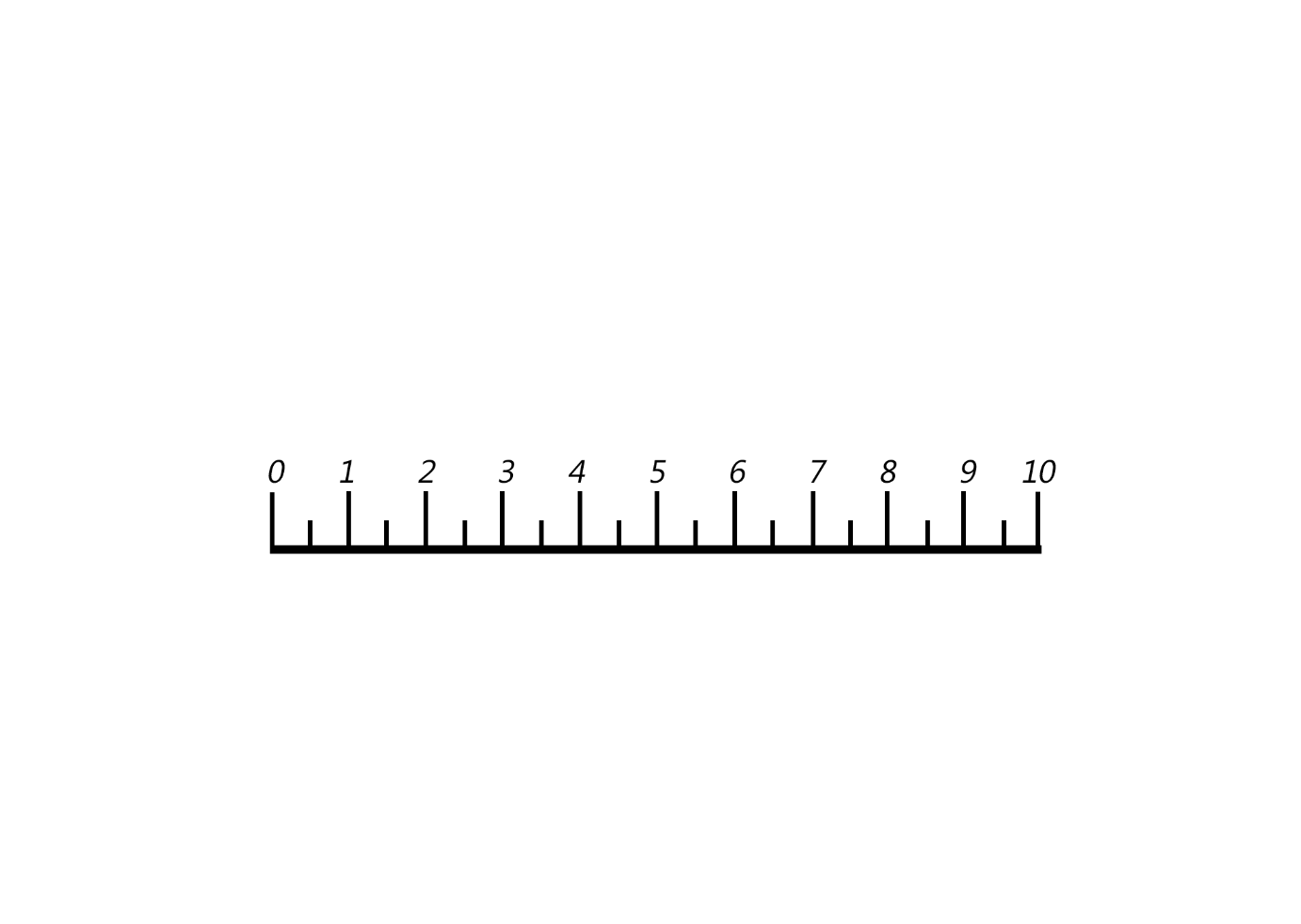


**Agitation**

The person with dementia:

1. appears agitated and restless, continuously seeks attention by shouting and complaining;
2. grabs at those passing by or obsessively asks for help;
3. tries to escape from the visit location or cannot remain seated;

*Extent of behavior*


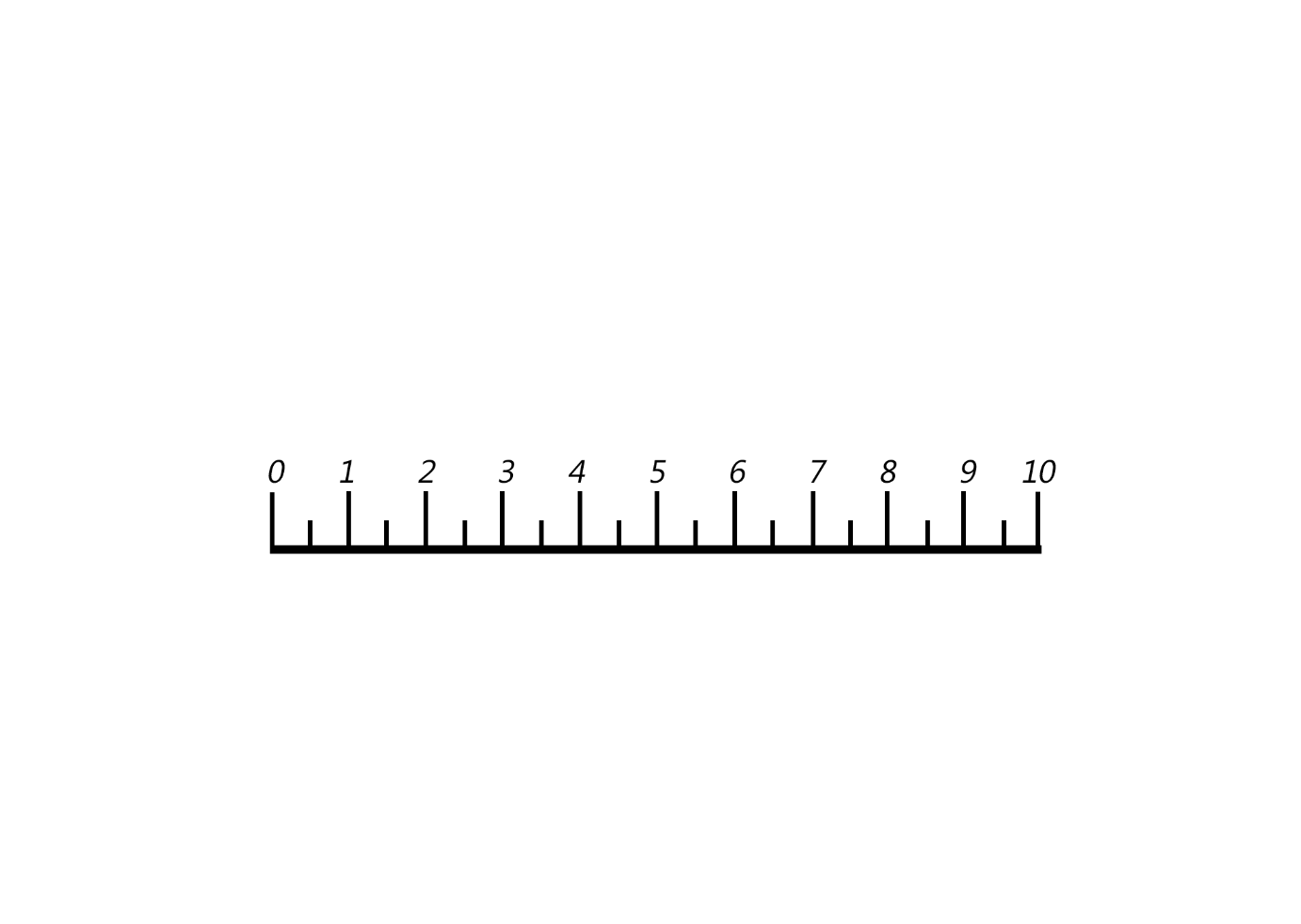


**Purposeless behaviors**

The person with dementia:

1. walks around the room without a specific purpose or rummages through objects or the desk;

2. repeatedly performs specific gestures, such as twisting or rubbing hands or feet, rocking the head;

3. keeps putting on and taking off clothes;

*Extent of behavior*


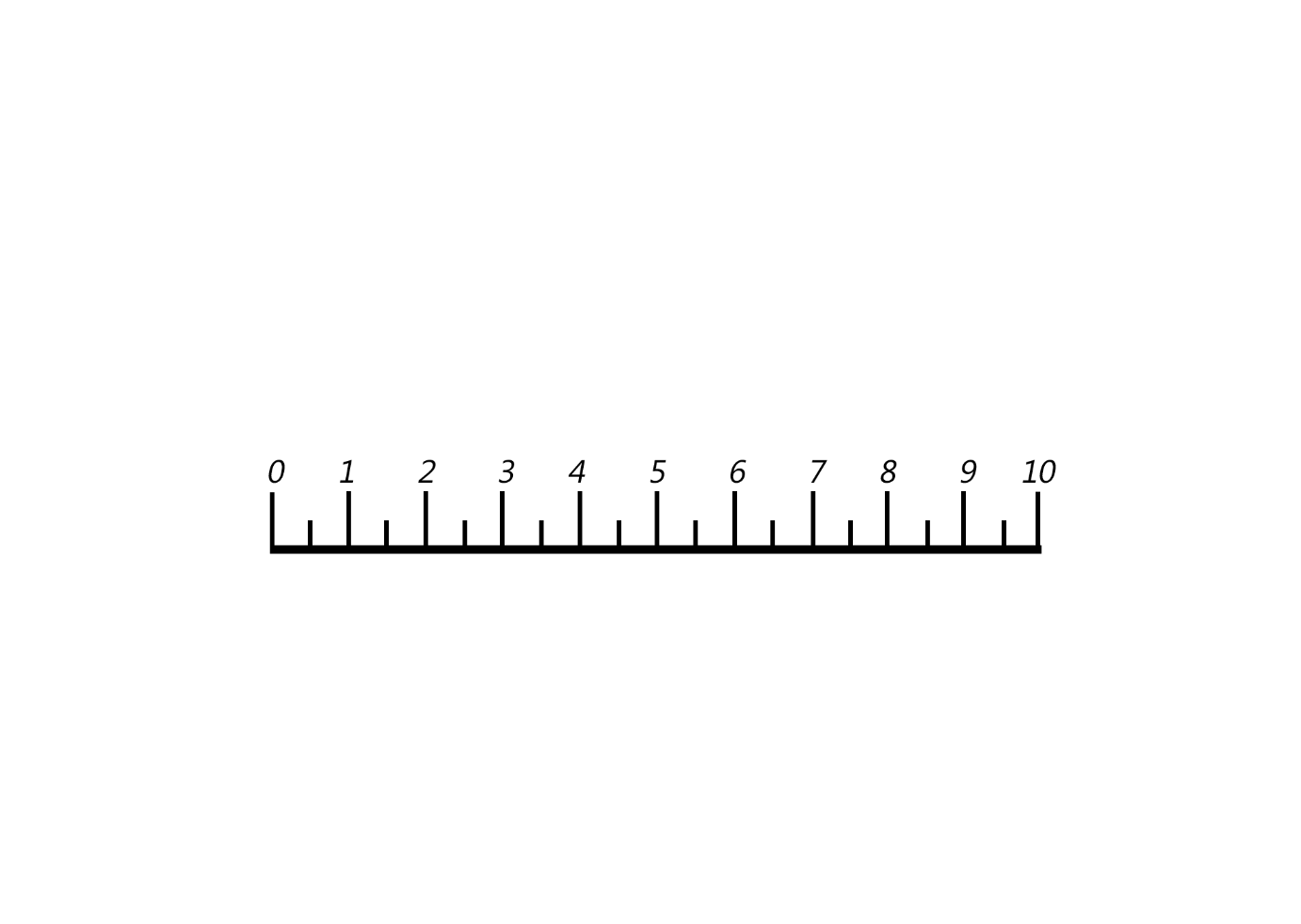


**Verbal aggression**

The person with dementia:

1. displays verbal aggression towards the healthcare provider or others present, insulting them, using derogatory epithets, and using foul language;
2. raises his/her voice or uses an aggressive tone when speaking;

*Extent of behavior*


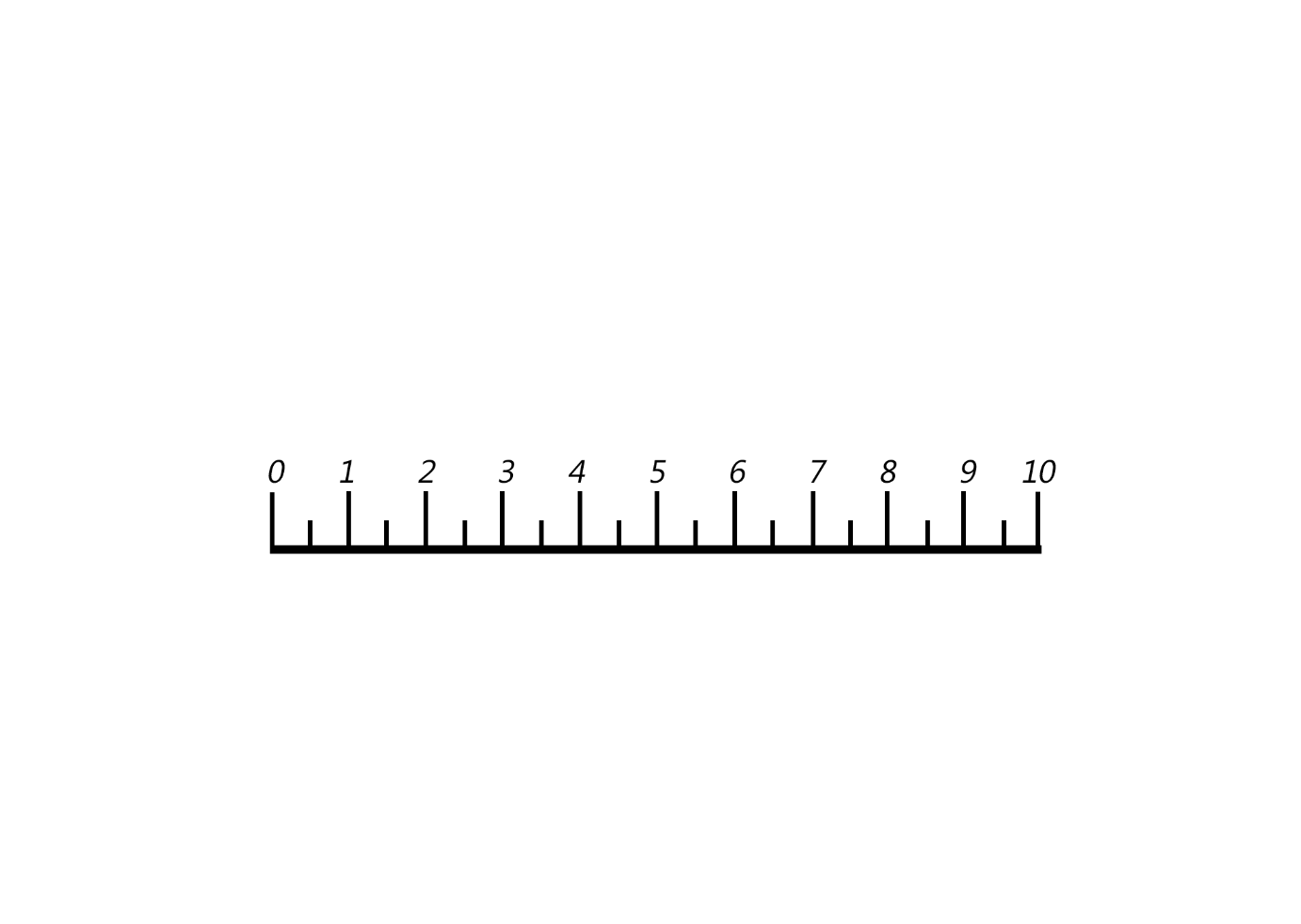


**Physical aggression**

The person with dementia:

1. displays physical aggression towards the healthcare provider or others present by punching, slapping, kicking, biting, spitting, scratching, or throwing objects;
2. destroys objects, damages them, or tears them apart;
3. becomes aggressive or resists during certain activities, such as undressing, dressing, or undergoing the examination;

*Extent of behavior*


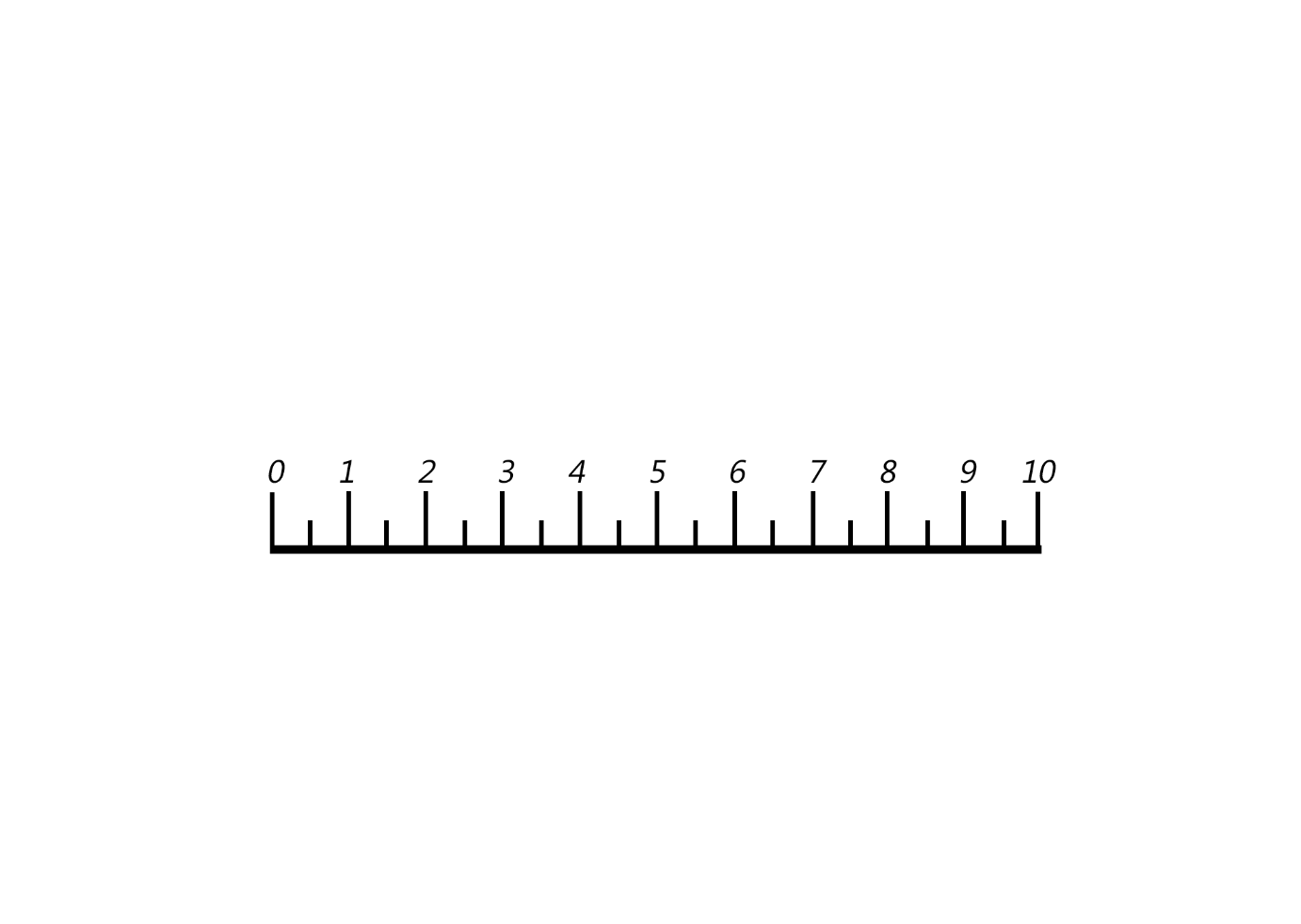


**Irritability**

The person with dementia:

1. is easily irritable;
2. does not accept any feedback; reacts nervously to even the slightest request or any mention of his/her cognitive deficits or his/her inability to perform certain tasks;
3. has highly variable mood swings;
4. exhibits sudden or unjustified outbursts of anger;

*Extent of behavior*


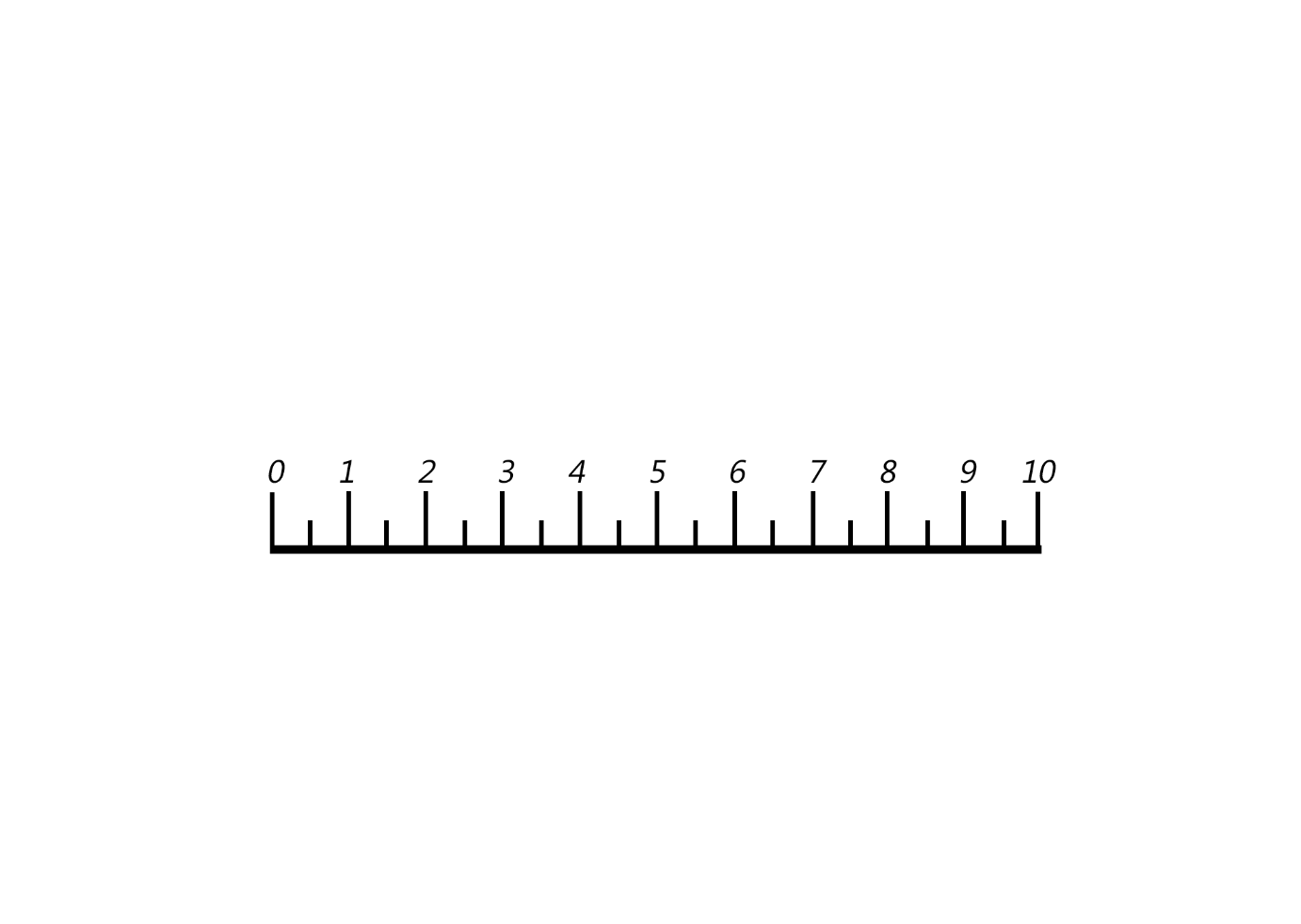


**Delusions**

The person with dementia:

1. exhibits delusional beliefs, such as thinking that someone is stealing from him/her or hiding his/her personal belongings;
2. is convinced that his/her spouse is cheating on him/her or that family members want to abandon him/her;
3. feels persecuted or believes that someone wants to harm him/her or poison him/her;
4. believes that the house is not his/her or that there is another identical one;
5. believes that family members are impostors or reacts with anger upon seeing their own reflection in the mirror;

*Extent of behavior*


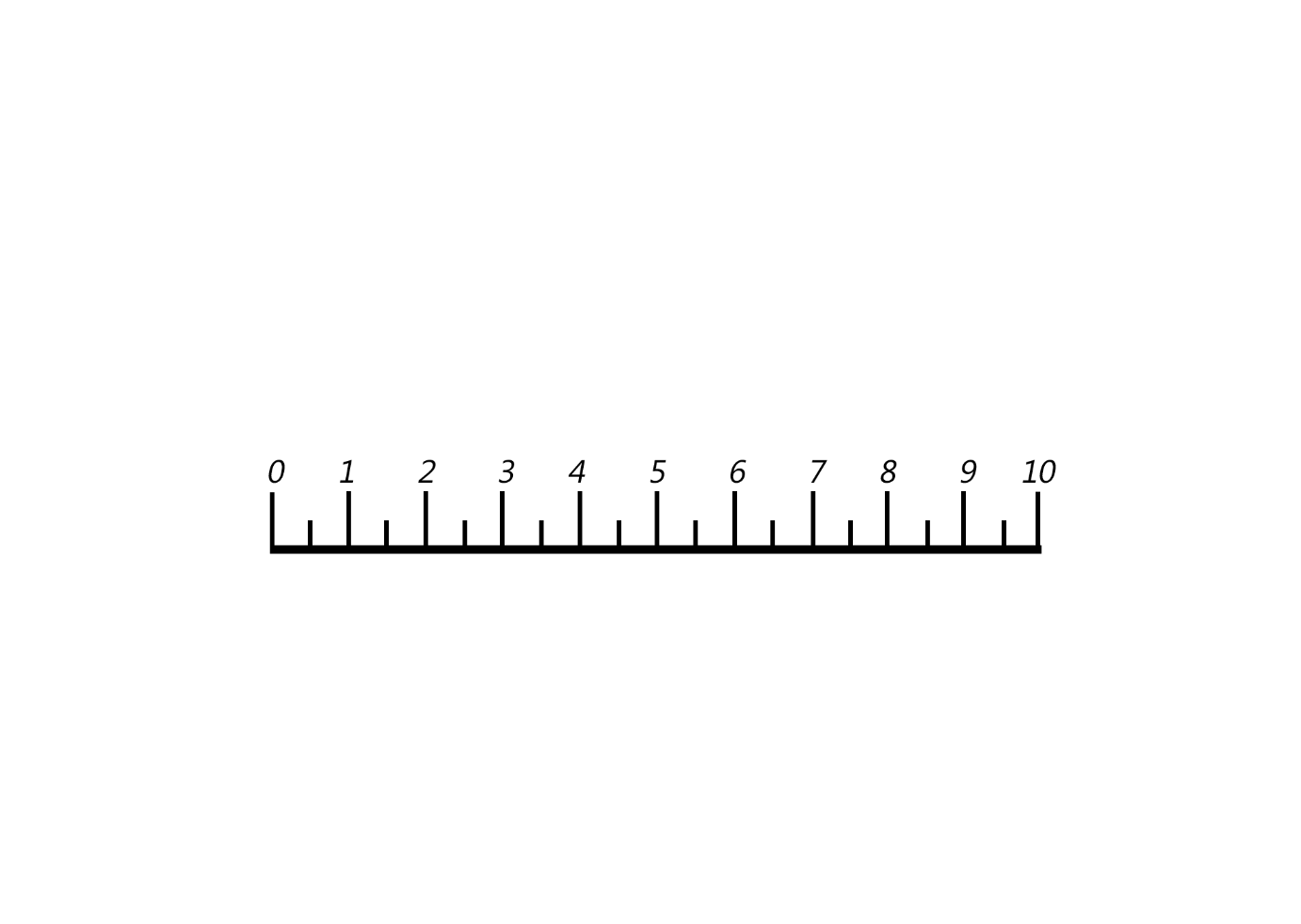


**Hallucinations**

The person with dementia:

1. displays or behaves as if experiencing visual hallucinations (e.g., claims there are nonexistent people or animals in the room);

2. auditory (e.g., hears imaginary sounds or noises);

3. olfactory (reports nonexistent smells or scents);

4. claims to feel touched by something or someone that does not exist, feels crawling animals on the skin (e.g., insects), scratches without any apparent reason;

5. complains of tastes or visceral sensations (e.g., abdominal pain) without plausible cause;

6. displays distorted sensory perceptions;

*Extent of behavior*


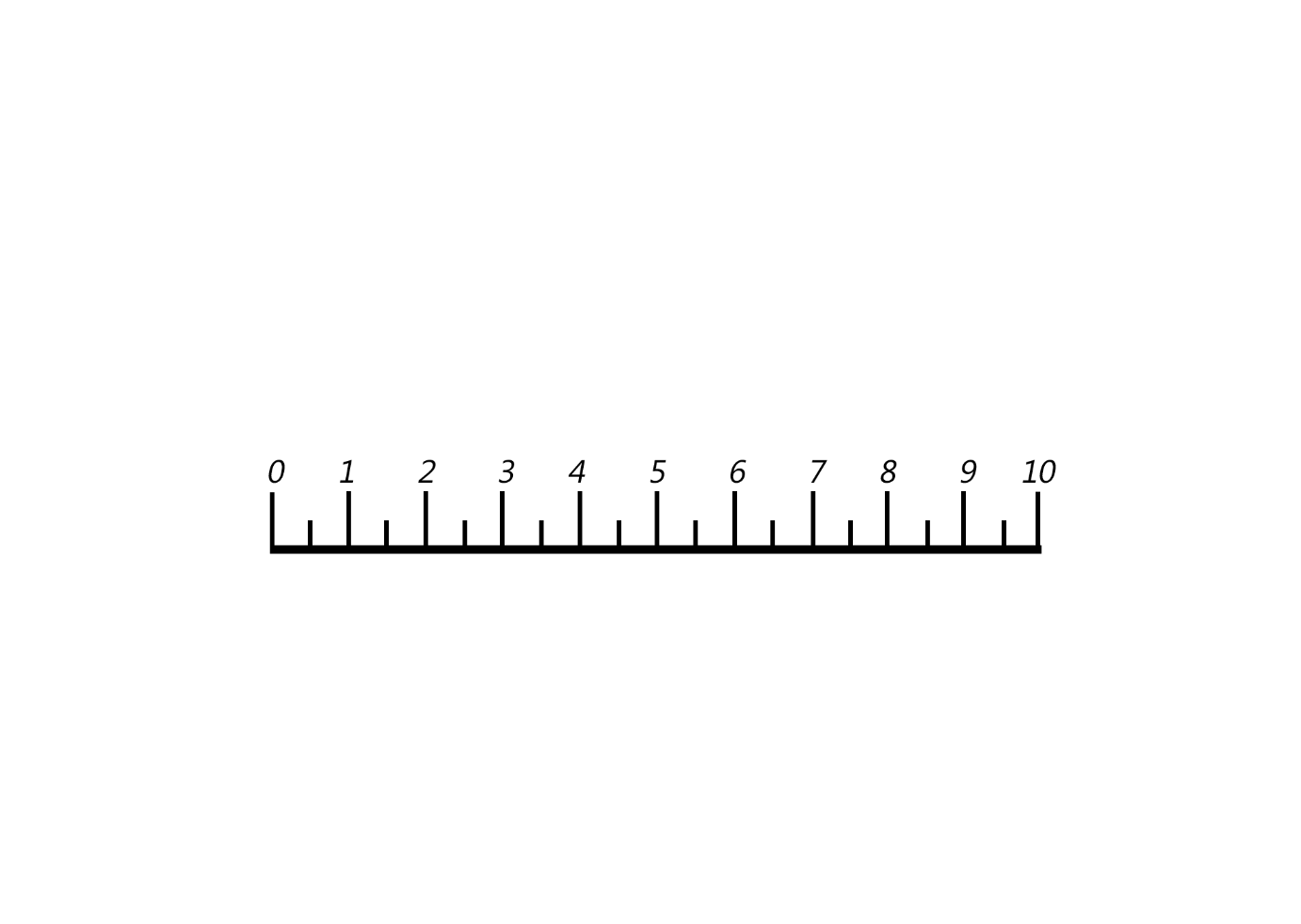


**Euphoria**

The person with dementia:

1. displays excessive or inappropriate cheerfulness during the visit/activity, appears euphoric and playful, makes silly jokes;

2. mocks healthcare staff or other present individuals, even at the risk of offending them;

3. laughs without reason;

4. finds things funny or ridiculous that are not so, or reacts with cheerfulness when discussing sad topics;

*Extent of behavior*


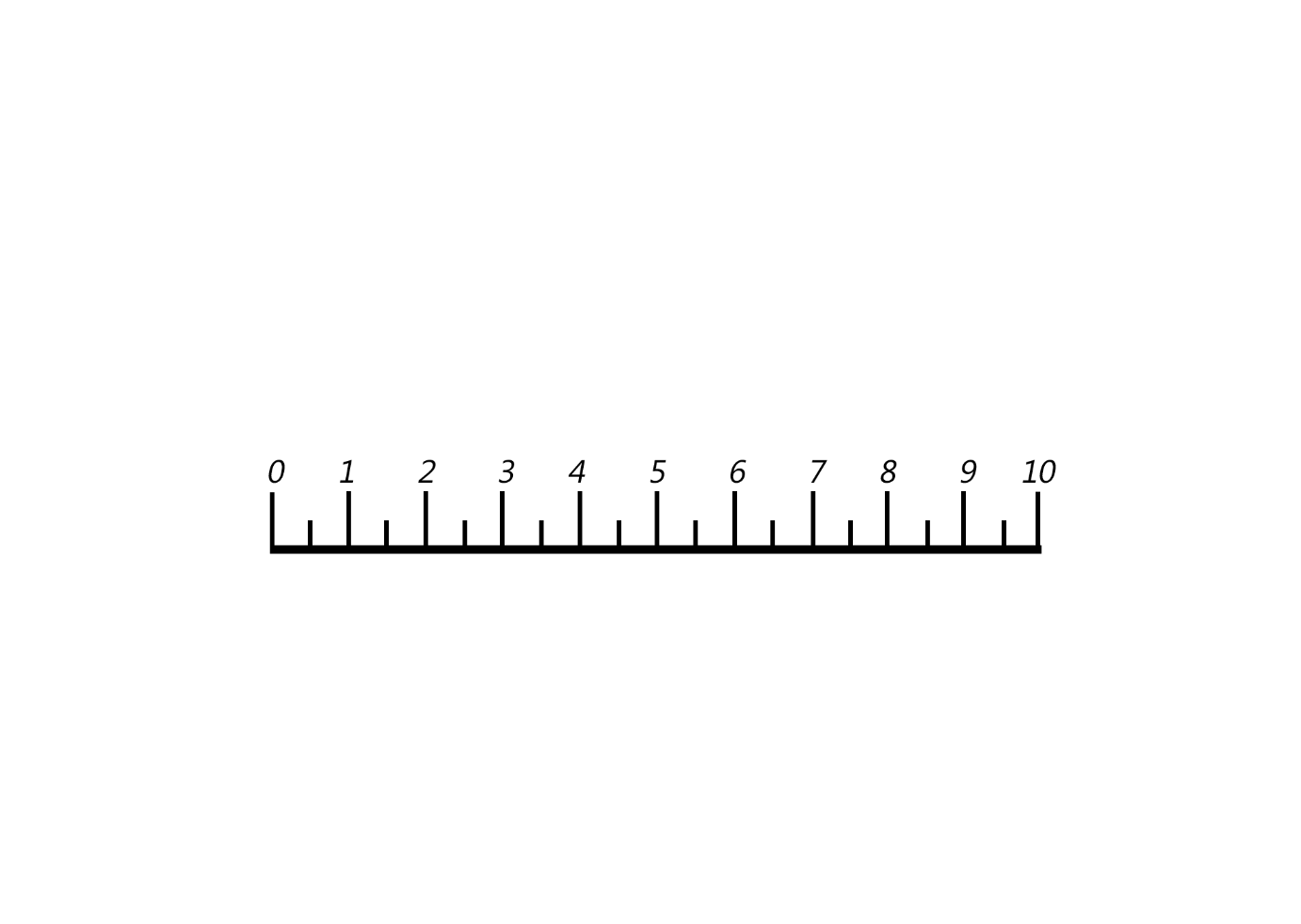


**Disinhibition**

The person with dementia:

1. behaves impulsively or uninhibitedly, does embarrassing things;

2. uses vulgar language, disregards social conventions, and behaves rudely;

3. speaks to healthcare staff in an excessively familiar manner;

4. makes sexual advances, undresses, tries to touch or grope those present, or engages in genital manipulation during the visit/activity;

*Extent of behavior*


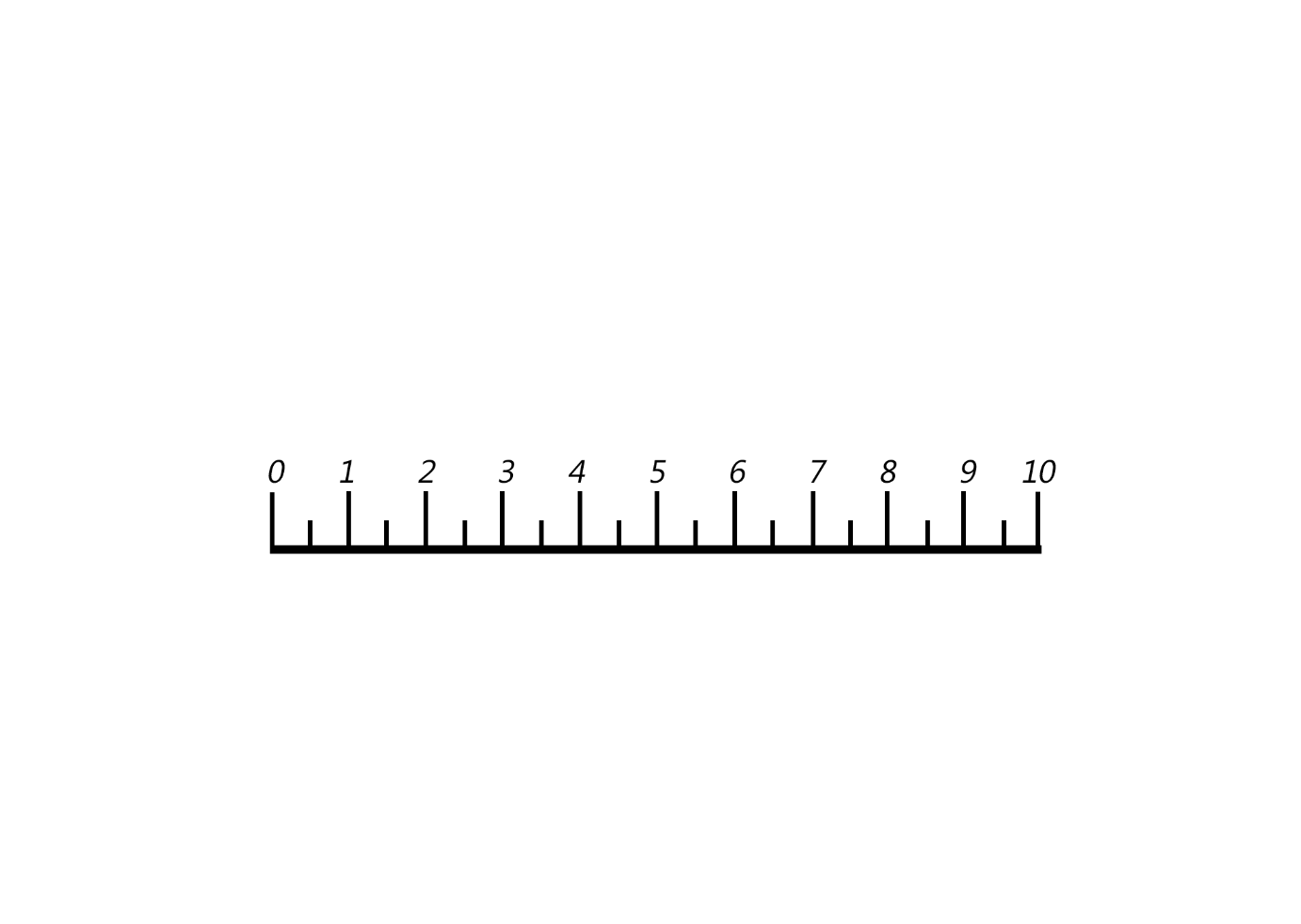


**Sleep/wake disturbances**

The person with dementia:

1. exhibits fluctuations in alertness, at certain moments during the visit or activity, appears "dazed”;

2. tends to fall asleep during the visit/activity, and needs to be prompted to stay awake;

*Extent of behavior*


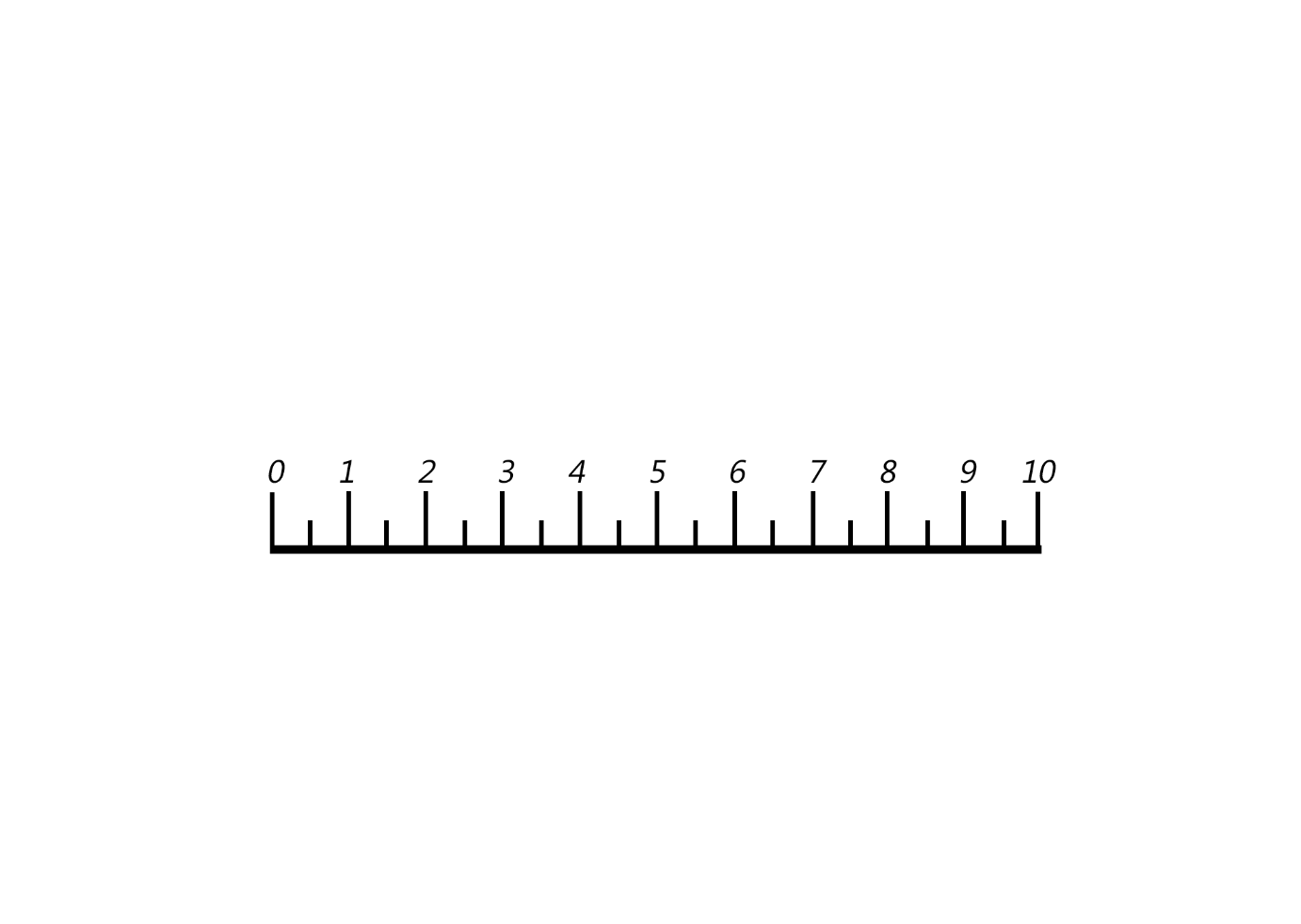


**Repetitive questions**

The person with dementia:

1. keeps repeating the same questions during the visit/activity;

2. continuously asks what day it is or where she/he is; repeats questions about deadlines or appointments;

3. returns to the same topics in a highly repetitive manner;

4. repeatedly seeks confirmation from the caregiver;

*Extent of behavior*


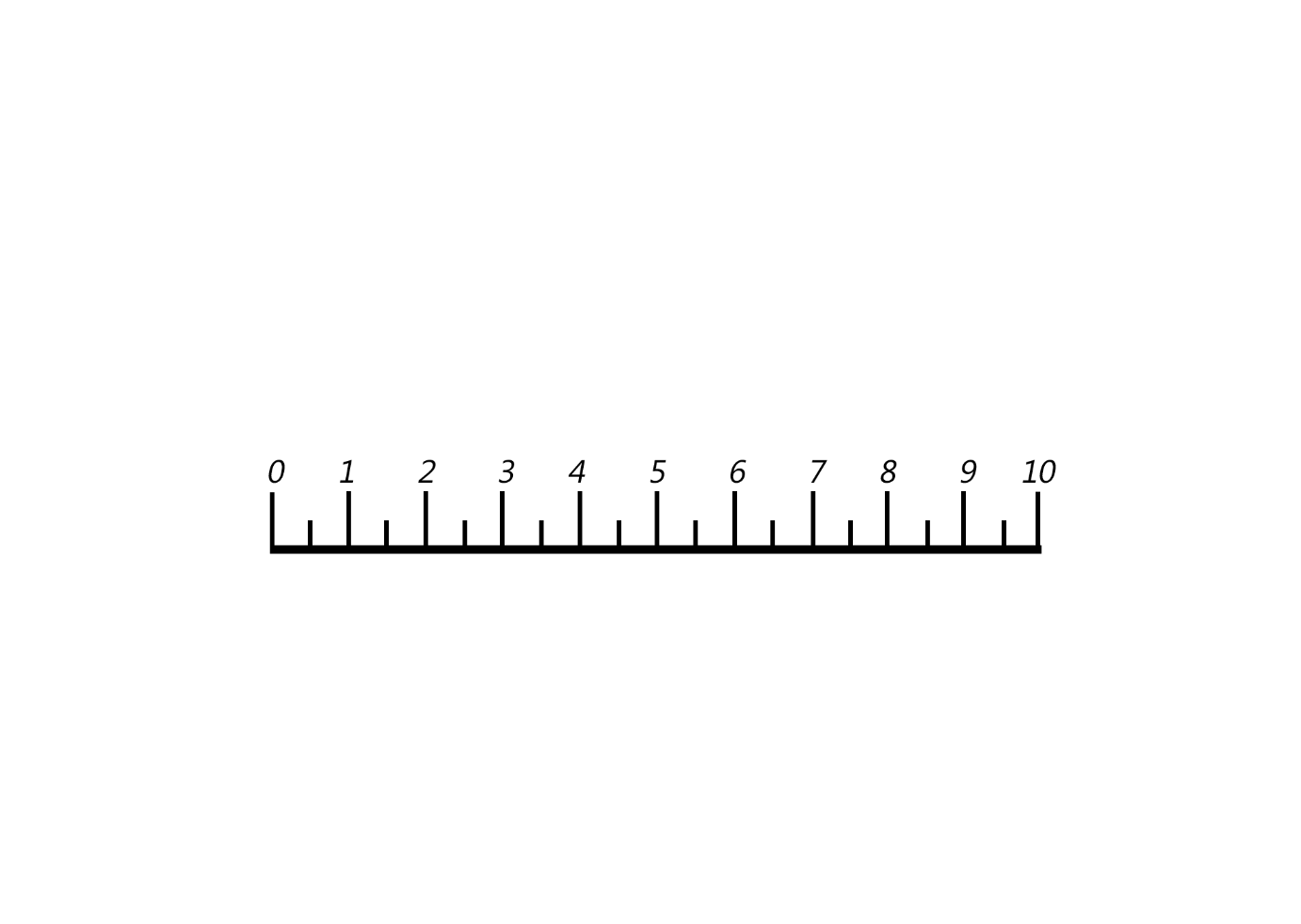


**Eating disturbances**

The person with dementia:

1. appears noticeably thinner and/or sarcopenic compared to the last visit;

2. appears to have gained significant weight (assessment can be done "by eye" or preferably using a scale);

3. continuously asks for food or drink or refuses to drink when offered;

*Extent of behavior*


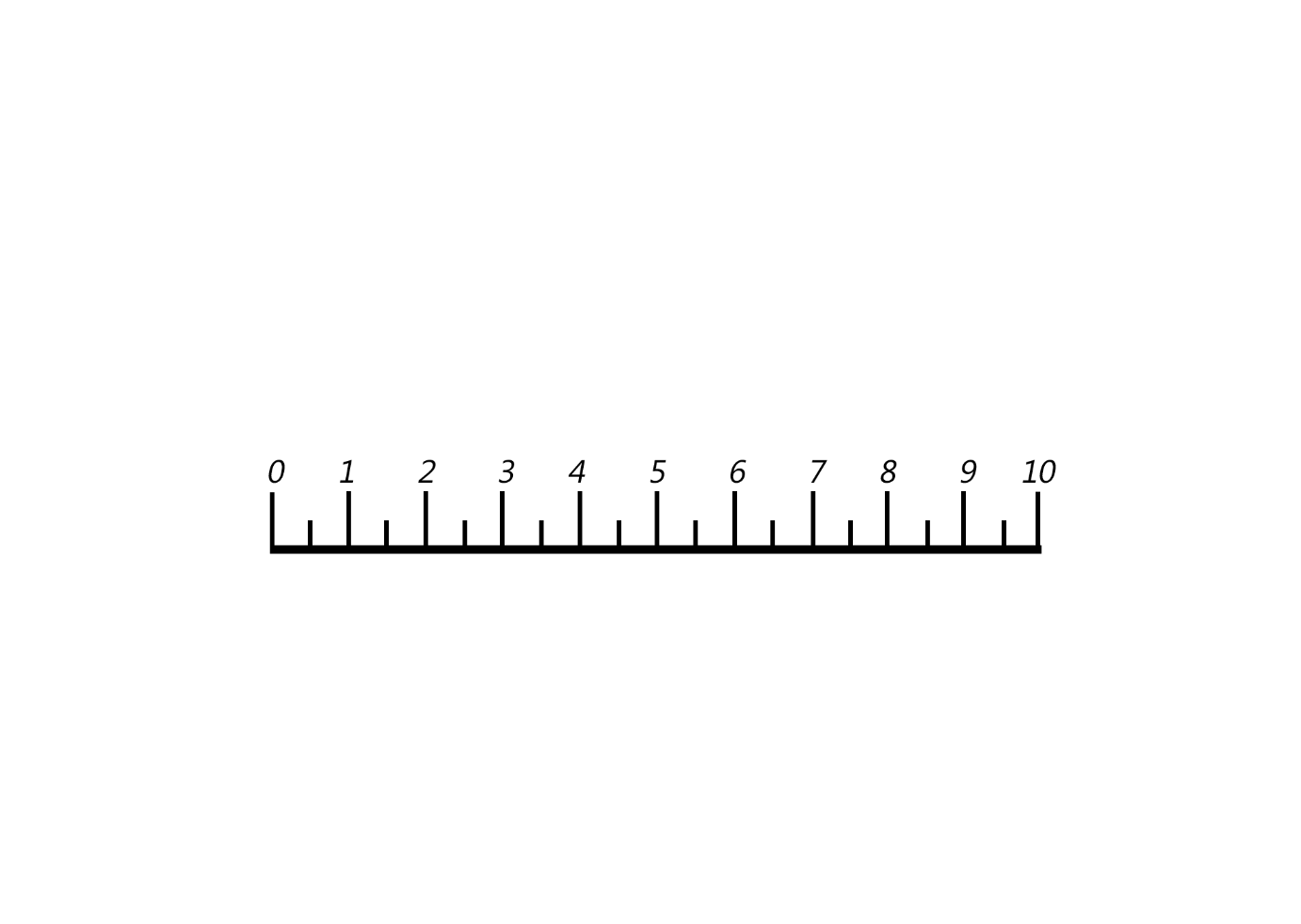


**Environmental dependency**

The person with dementia:

1. appears more agitated and confused in the final minutes of the visit/activity compared to the initial ones;

2. displays agitation or anxiety if the weather changes during the activity/visit;

*Extent of behavior*


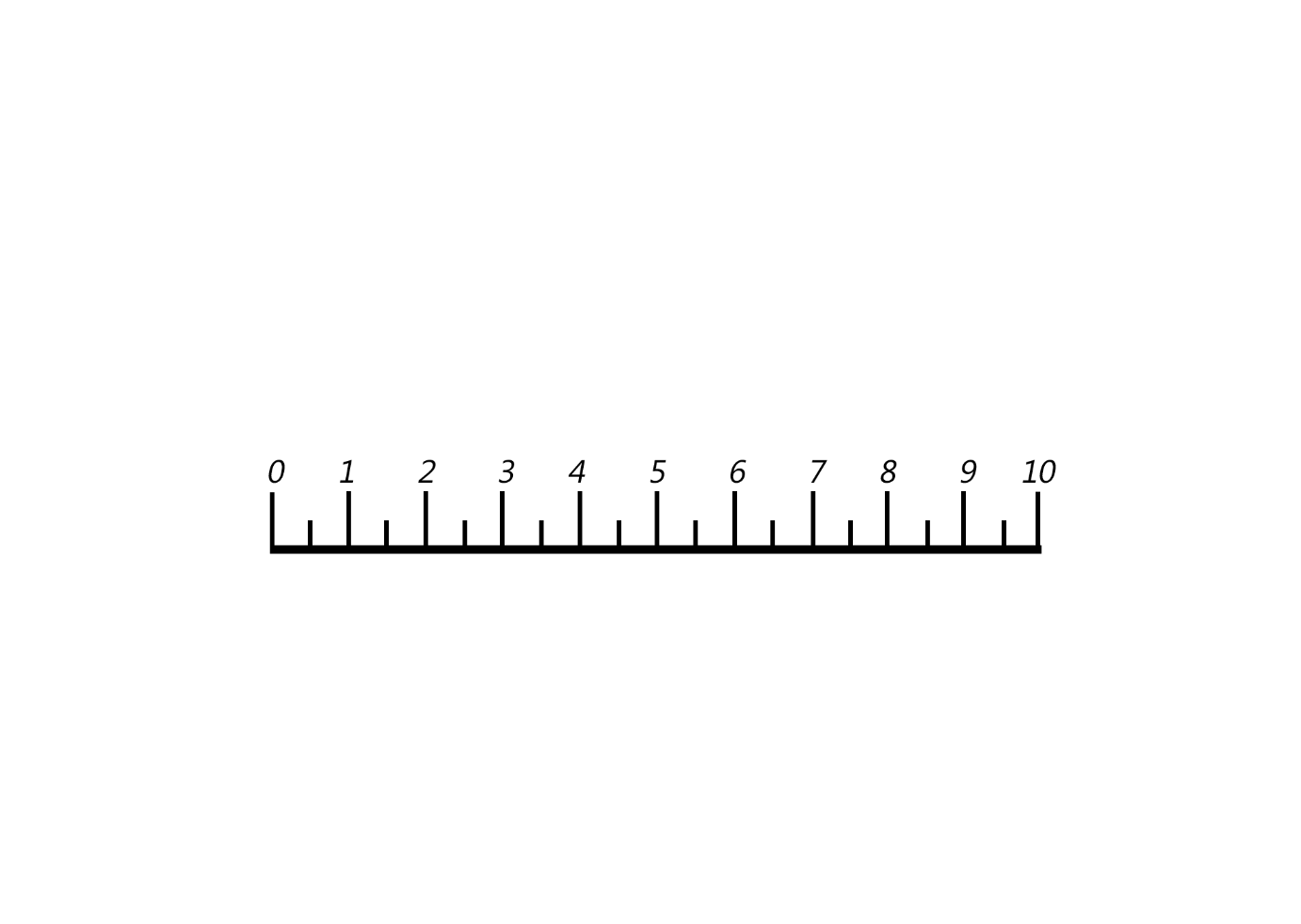


**_____________________________________________________________________**

"We thank you for answering the questions. Your responses will enhance the care of the person with dementia. Please also respond to some questions regarding yourself and note the ongoing treatments for BPSD."

Please indicate your age____

Education in years___________
Gender  M  F  Unspecified

Profession :

- Physician
- Nurse
- Psychologist
- TDR
- Educator
- Psychomotor therapist
- Auxiliary
- Other

Had already assessed/encountered the person with dementia

 YES  NO

Ongoing pharmacological treatment for BPSD:

NO 

YES 

 neuroleptics_____________________________________________

 antidepressants __________________________________________

 benzodiazepines_________________________________________

 mood stabilizers_____________________________________________

 melatonin_____________________________________________

 supplements______________________________________________

 other____________________________________________________

Ongoing non-pharmacological treatment for BPSD:

NO 

YES 

 cognitive stimulation __________________________________

occupational/recreational therapy______________________

psychomotor therapy____________________________________

art therapy____________________________________________

music therapy__________________________________________

pet therapy_____________________________________________

Gentle care®/Person Centred Care_____________________

Doll therapy____________________________________________

aromatherapy__________________________________________

other___________________________________________________

Duration of today's visit/activity in minutes:________________________________________

**Scores**

|  |  | **Caregiver extent (C.E.)** | **Caregiver coping skills** | **Examiner**  **(EX)** | **Total I e II column ( C.E.+ E.X)** |
| --- | --- | --- | --- | --- | --- |
| 1 | Apathy |  |  |  |  |
| 2 | Depression |  |  |  |  |
| 3 | Anxiety |  |  |  |  |
| 4 | Compulsion |  |  |  |  |
| 5 | Agitation |  |  |  |  |
| 6 | Purposeless behaviors |  |  |  |  |
| 7 | Verbal aggression |  |  |  |  |
| 8 | Physical aggression |  |  |  |  |
| 9 | Irritability |  |  |  |  |
| 10 | Delusions |  |  |  |  |
| 11 | Hallucinations |  |  |  |  |
| 12 | Euphoria |  |  |  |  |
| 13 | Disinhibition |  |  |  |  |
| 14 | Sleep/wake disturbance |  |  |  |  |
| 15 | Repetitive questions |  |  |  |  |
| 16 | Eating disturbances |  |  |  |  |
| 17 | Environmental dependency |  |  |  |  |
| **Total** | |  |  |  |  |
