# Supplementary material for: Italian standardization of the BPSD-SINDEM scale for the assessment of neuropsychiatric symptoms in persons with dementia
Source: Front Neurol. 2024 Nov 21;15:1455787. doi: 10.3389/fneur.2024.1455787 (PMC11617322; doi:10.3389/fneur.2024.1455787)
Supplement: Supplementary file 4 [file Data_Sheet_3.docx]

**SCALA PER I DISTURBI PSICOCOMPORTAMENTALI DELLA SINDEM (SDPC-SINDEM)**

**SOTTOSCALA OSSERVAZIONALE PER L’ESAMINATORE**

Risponda alle domande riferendosi alla Sua *osservazione diretta del comportamento* della persona affetta da demenza *durante la visita/attività*. Si vuole ottenere una “fotografia” puntuale del comportamento del soggetto in quel momento, comportamenti che la persona con demenza ha avuto in altri momenti non vanno tenuti in considerazione. I *comportamenti osservati devono essere graduati* secondo una scala che valuta l’entità del comportamento stesso e che va da 0 (il comportamento non è presente) a 10 (l’entità del comportamento è la maggiore che si possa immaginare; l’entità va valutata sulla base dell’intensità e della frequenza del comportamento durante l’esame). Apponga una crocetta fra 0 e 10 per graduare l’entità. Le chiediamo inoltre di evidenziare, nella descrizione relativa ad ogni comportamento, i punti che descrivono i comportamenti osservati *(chi risponde dovrebbe usare una matita con gommino – se non disponibile una penna- e un evidenziatore).*

**Apatia**

La persona con demenza:

1. appare scarsamente interessata alla visita/attività, alle persone presenti e a quanto la circonda, si estrania dal contesto e dalla conversazione;
2. non prende iniziative verbali e deve essere sempre spinta a fare le cose;
3. appare anaffettiva, indifferente rispetto alle emozioni altrui, ha scarse reazioni emotive.

*Entità del comportamento*


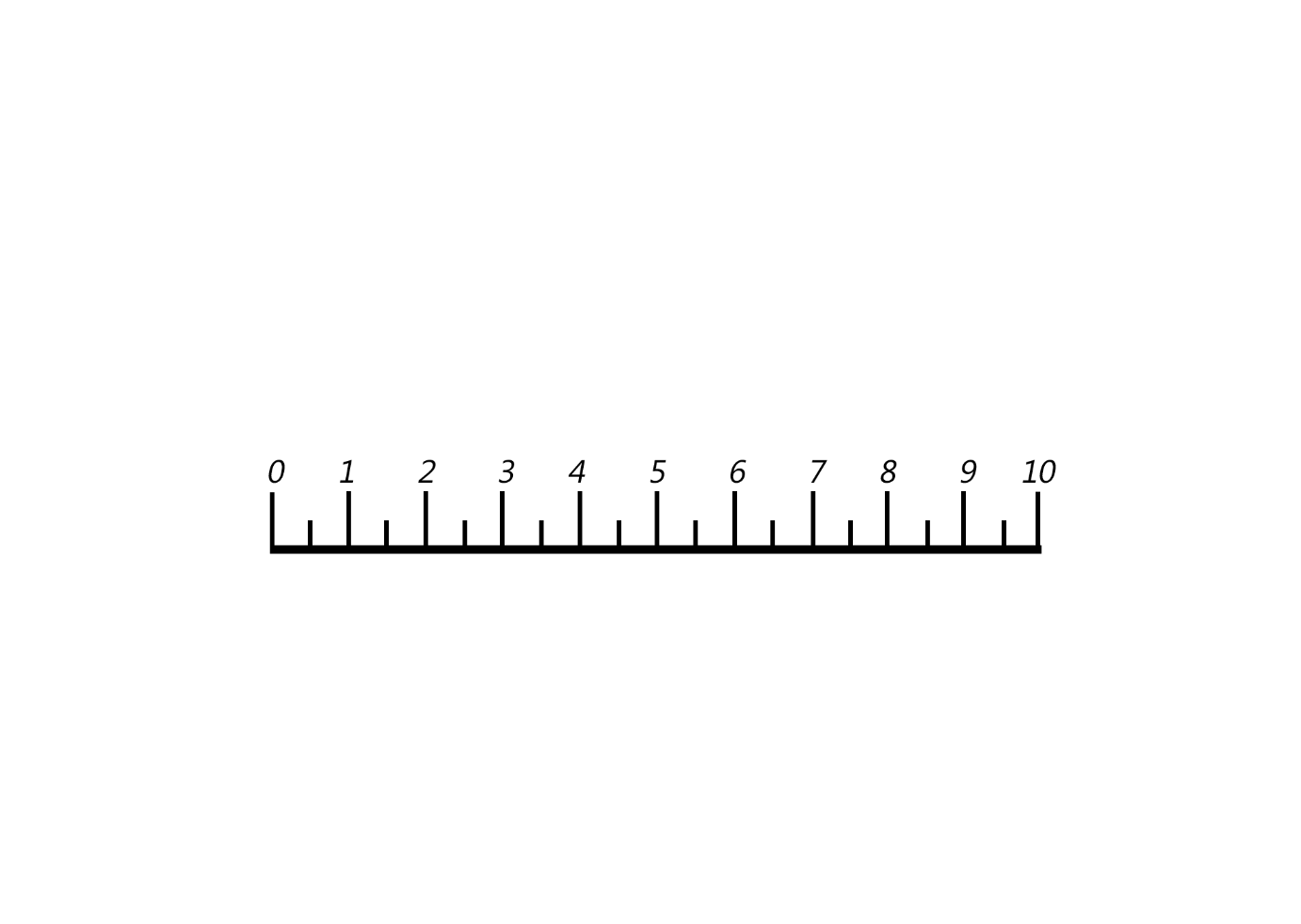


**Depressione**

La persona con demenza:

1. mostra segni di depressione, appare triste, scoraggiata, senza speranze, piange facilmente;
2. afferma che la vita non vale più la pena di essere vissuta;
3. afferma di non essere più lui/lei o di essere un/a buono/a a nulla;
4. lamenta disturbi fisici o malesseri che sembrano avere una causa psicologica.

*Entità del comportamento*


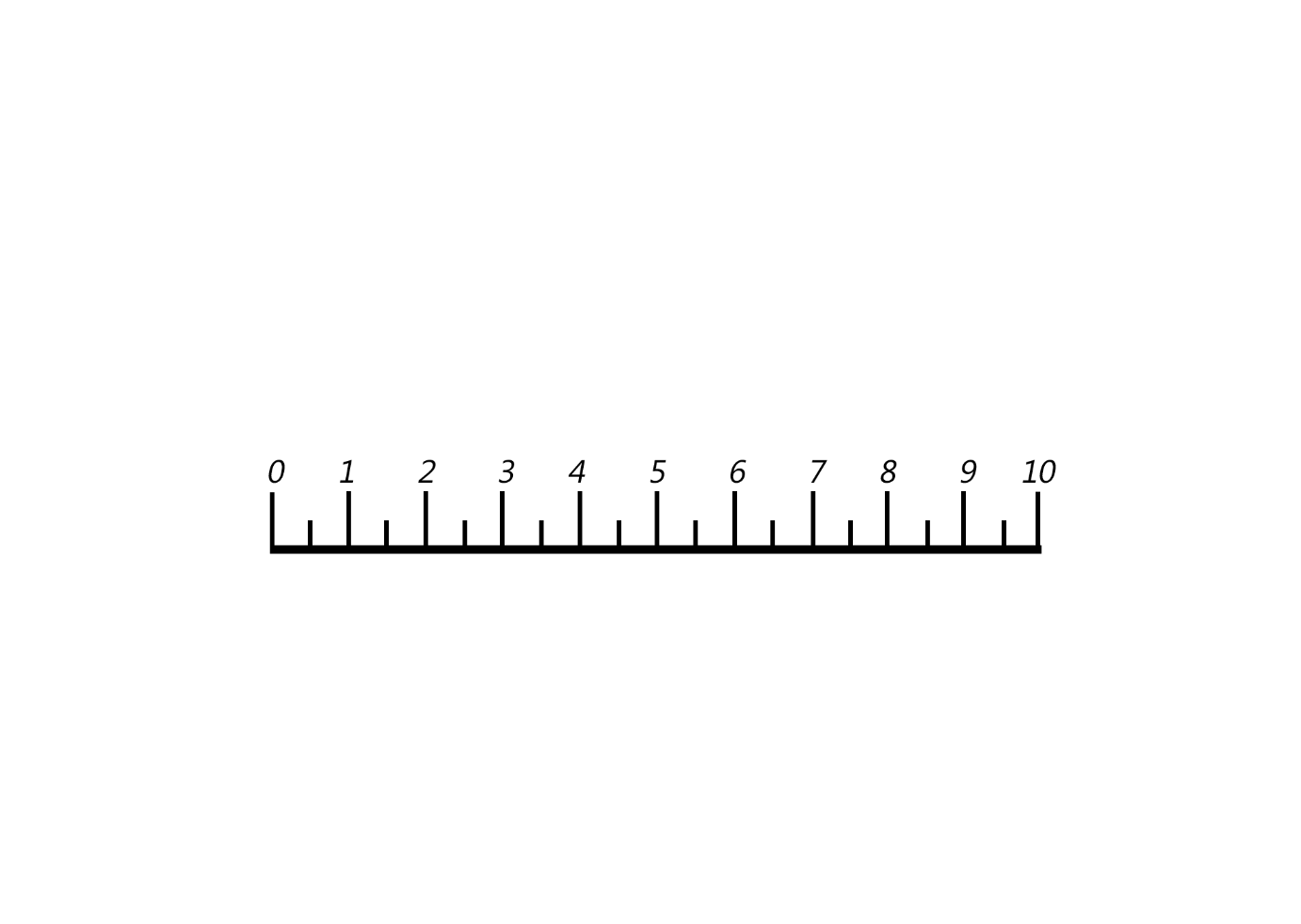


**Ansia**

La persona con demenza:

1. mostra sintomi di ansia;
2. è preoccupata perché si trova lontano da casa o se deve rimanere lontana dal caregiver;
3. ha paura di rimanere senza soldi;
4. ha paura di perdere la memoria o la propria salute;
5. non tollera le attese;
6. appare tesa o preoccupata in particolare se confrontata a cose nuove;
7. manifesta delle fobie.

*Entità del comportamento*


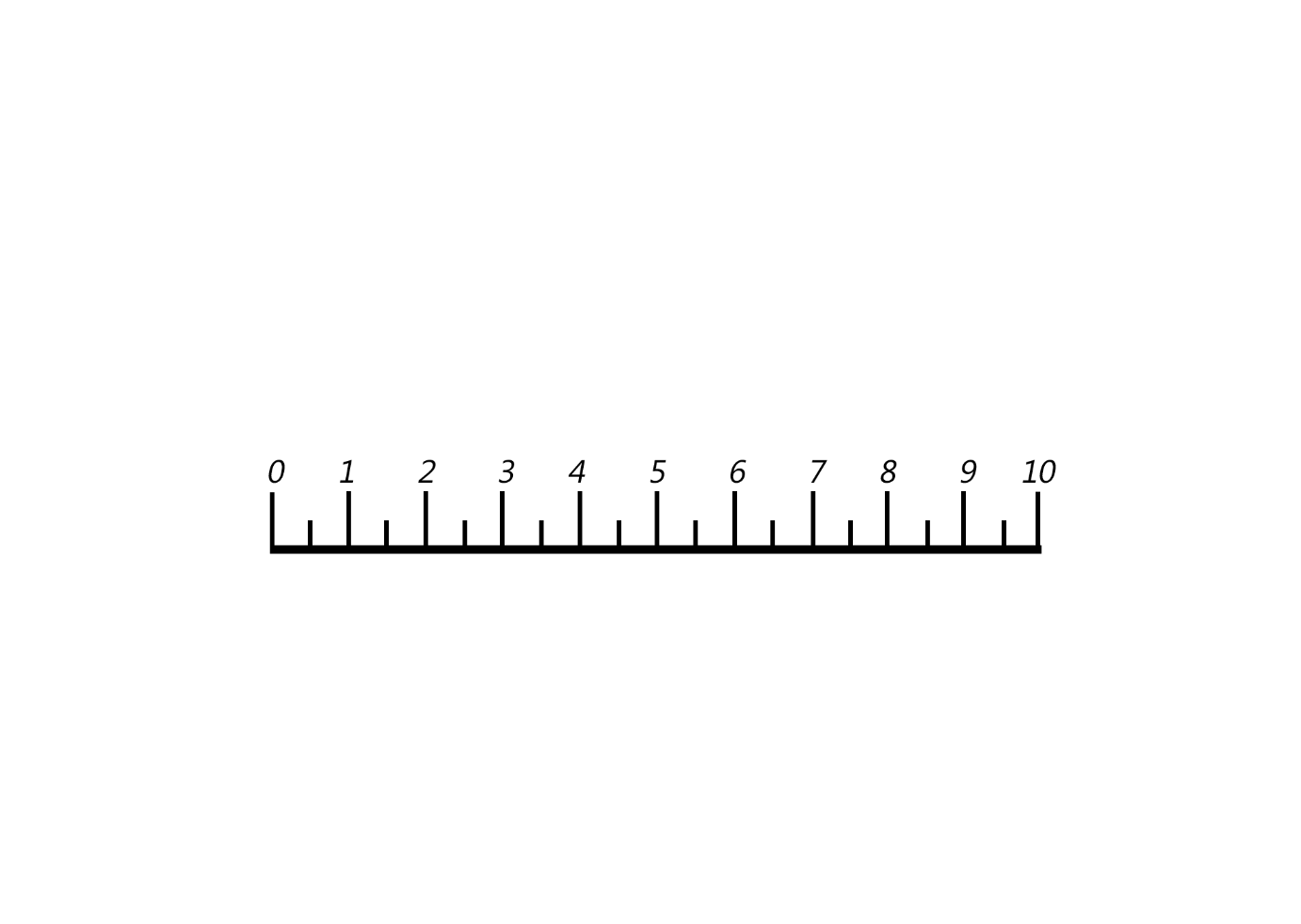


**Compulsioni**

La persona con demenza:

1. mostra comportamenti compulsivi, ad esempio ripete in continuazione precisi gesti o verbalizzazioni oppure osserva certi rituali;
2. raccoglie o tocca o mette in bocca ciò che trova in giro;
3. chiede ossessivamente di fumare o bere/mangiare.

*Entità del comportamento*


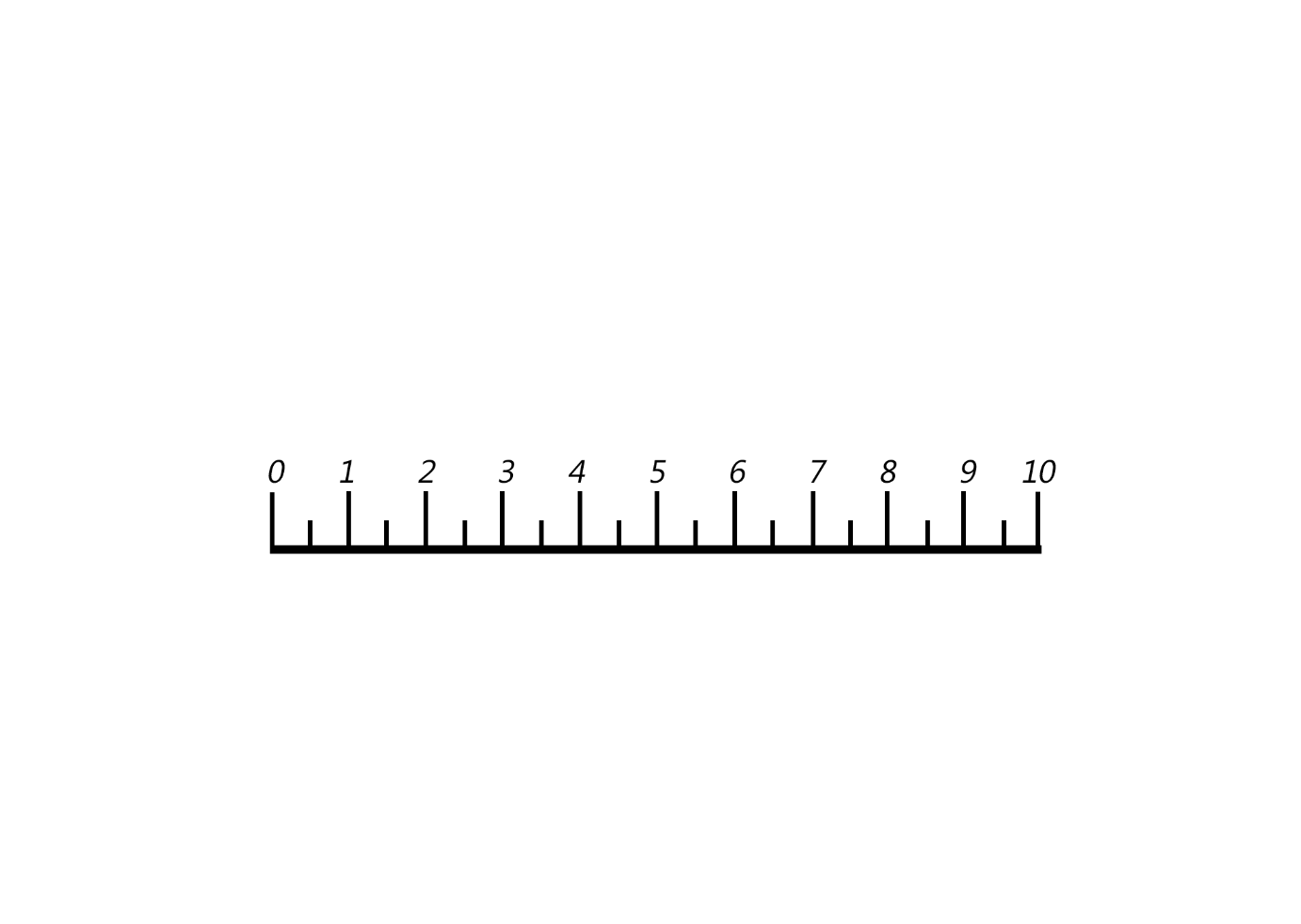


**Agitazione**

La persona con demenza:

1. si mostra agitata, inquieta, cerca di richiamare in continuazione l’attenzione, gridando, lamentandosi;
2. afferra chi le passa vicino o chiedendo aiuto ossessivamente;.
3. cerca di scappare dal luogo della visita o non riesce a stare seduta.

*Entità del comportamento*


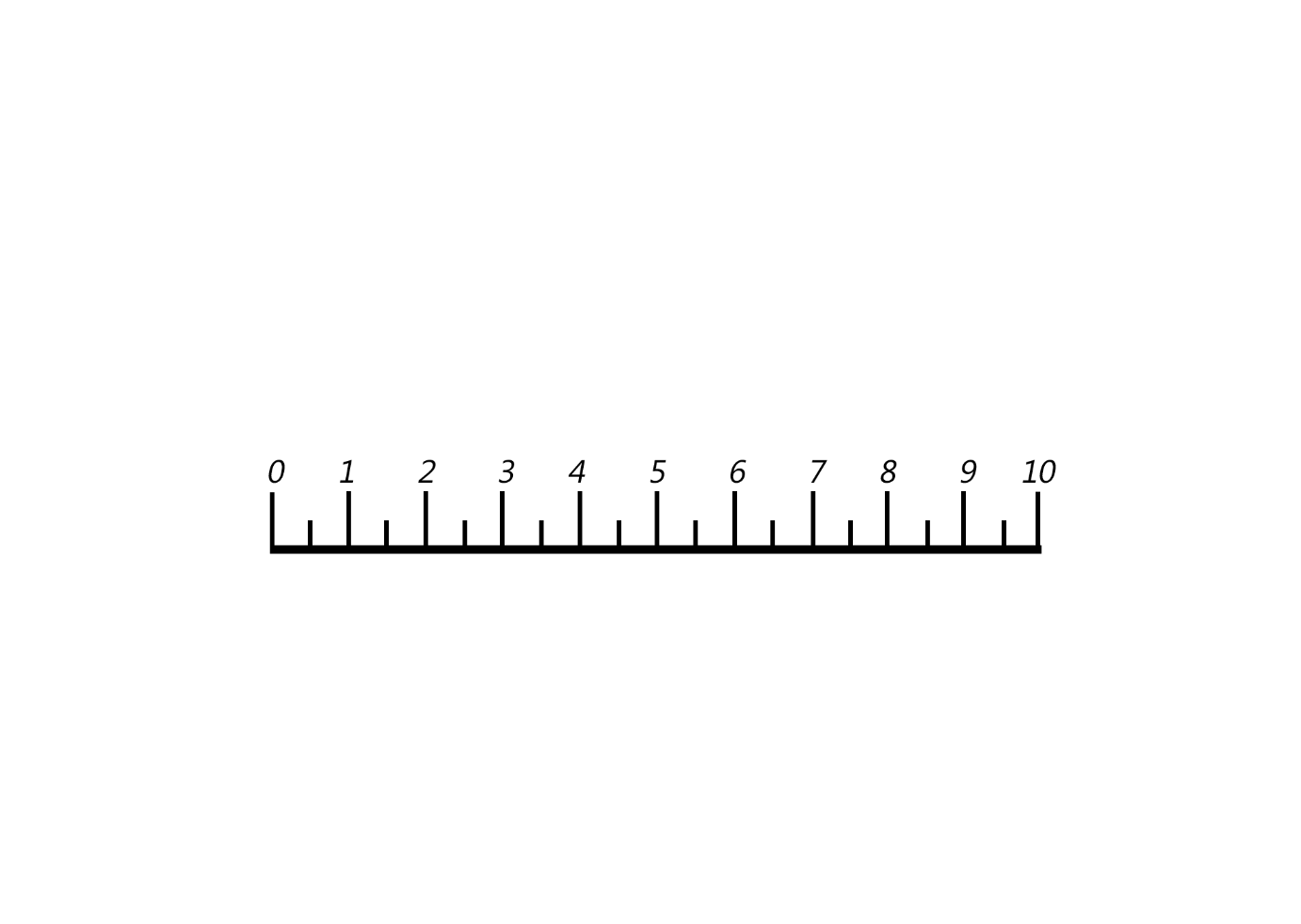


**Affaccendamento**

La persona con demenza:

1. cammina per la stanza senza uno scopo preciso oppure, rovista fra gli oggetti o sulla scrivania;
2. ripete determinati gesti in continuazione, torcendosi o strofinandosi le mani o i piedi, dondolando la testa;
3. continua a mettersi e togliersi i vestiti.

*Entità del comportamento*


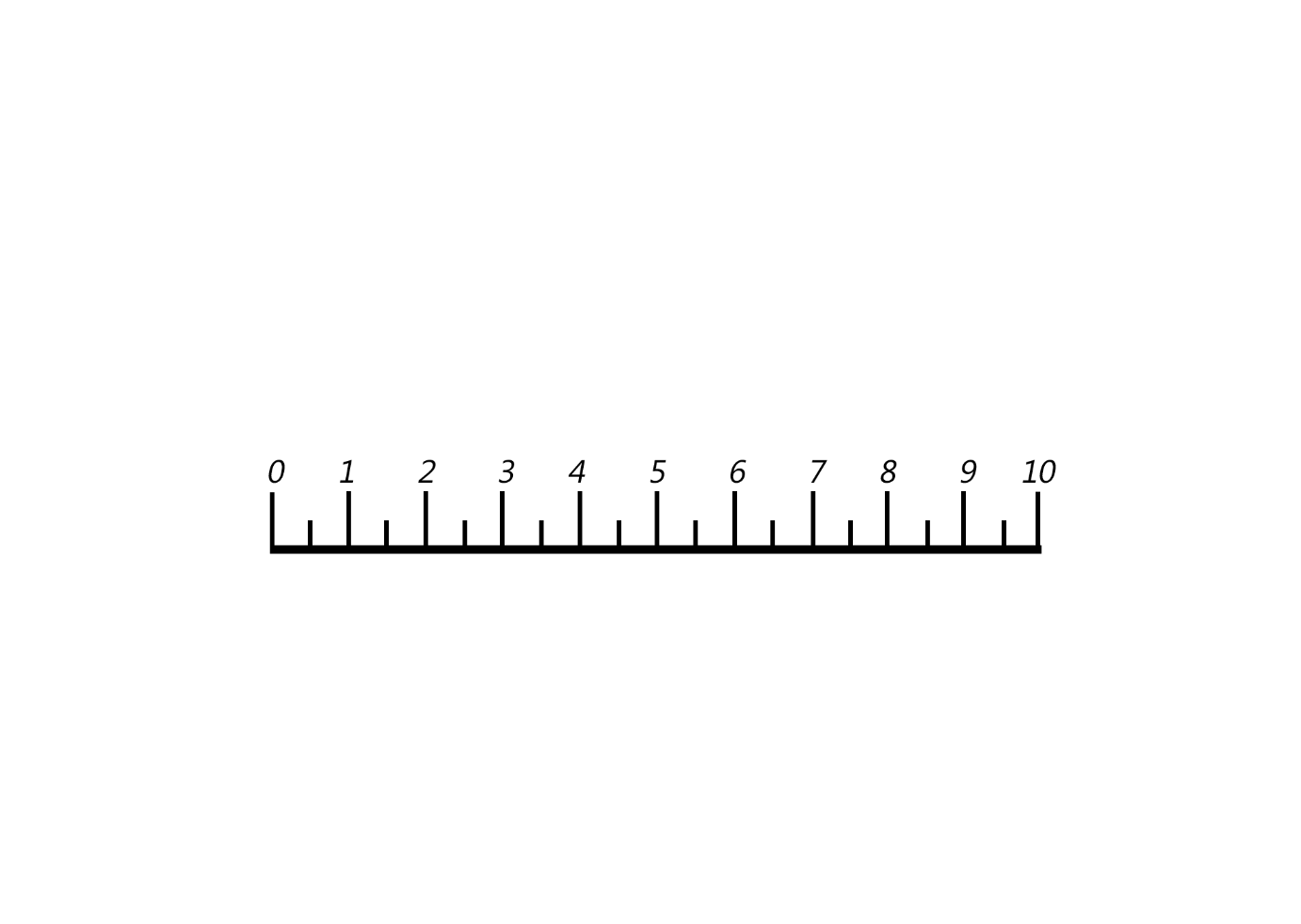


**Aggressività verbale**

La persona con demenza:

1. mostra aggressività verbale verso l’operatore sanitario o altri presenti, insultandoli, lanciando epiteti ingiuriosi, usando parolacce;
2. alza la voce o usa un tono aggressivo quando parla.

*Entità del comportamento*


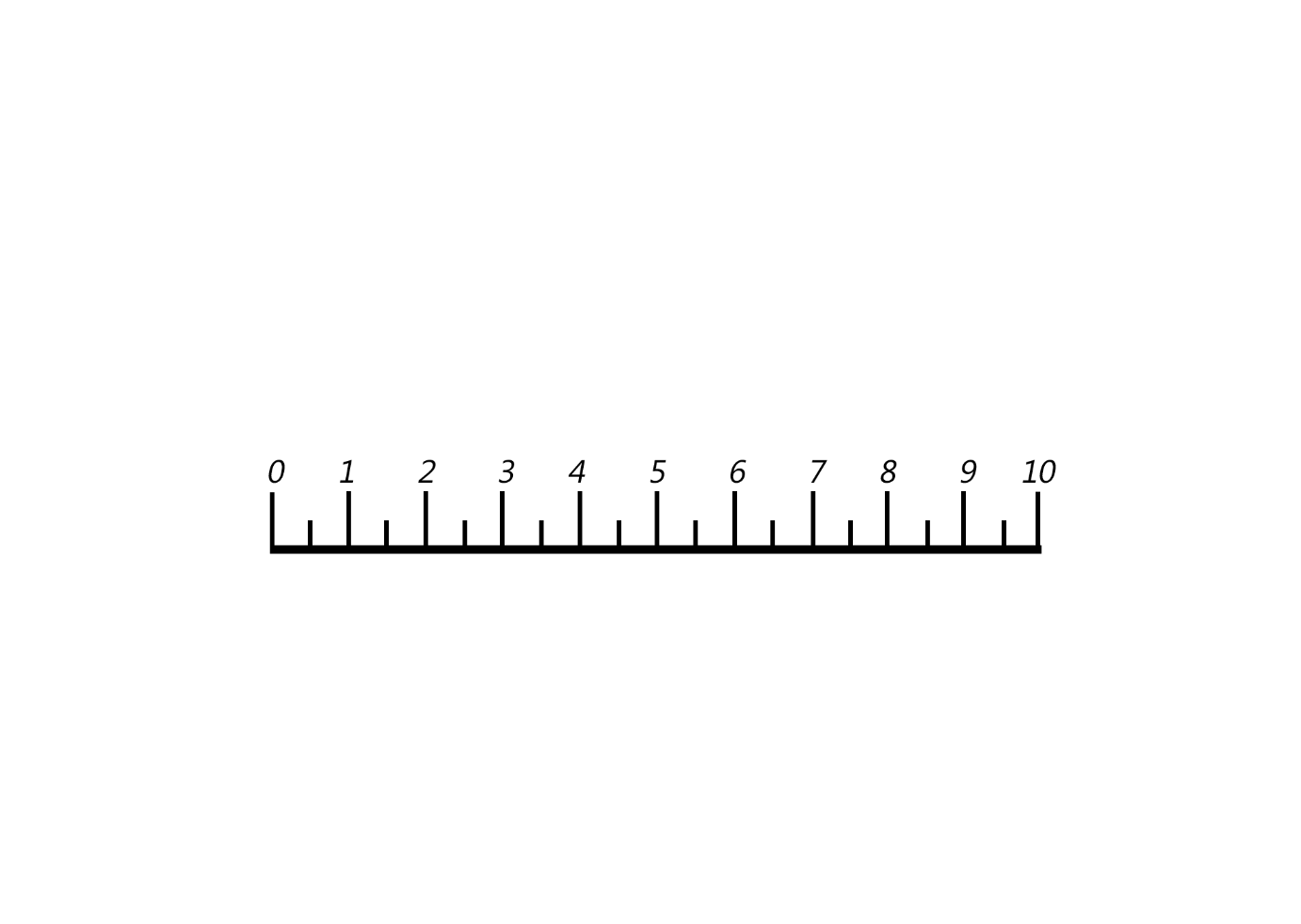


**Aggressività fisica**

La persona con demenza:

1. mostra aggressività fisica verso l’operatore sanitario o altri presenti, dando pugni o schiaffi, calciando, mordendo, sputando, graffiando o lanciando oggetti;
2. distrugge oggetti, li danneggia, li strappa;
3. diventa aggressiva o resiste durante certe attività, come svestirsi o vestirsi o eseguire la visita.

*Entità del comportamento*


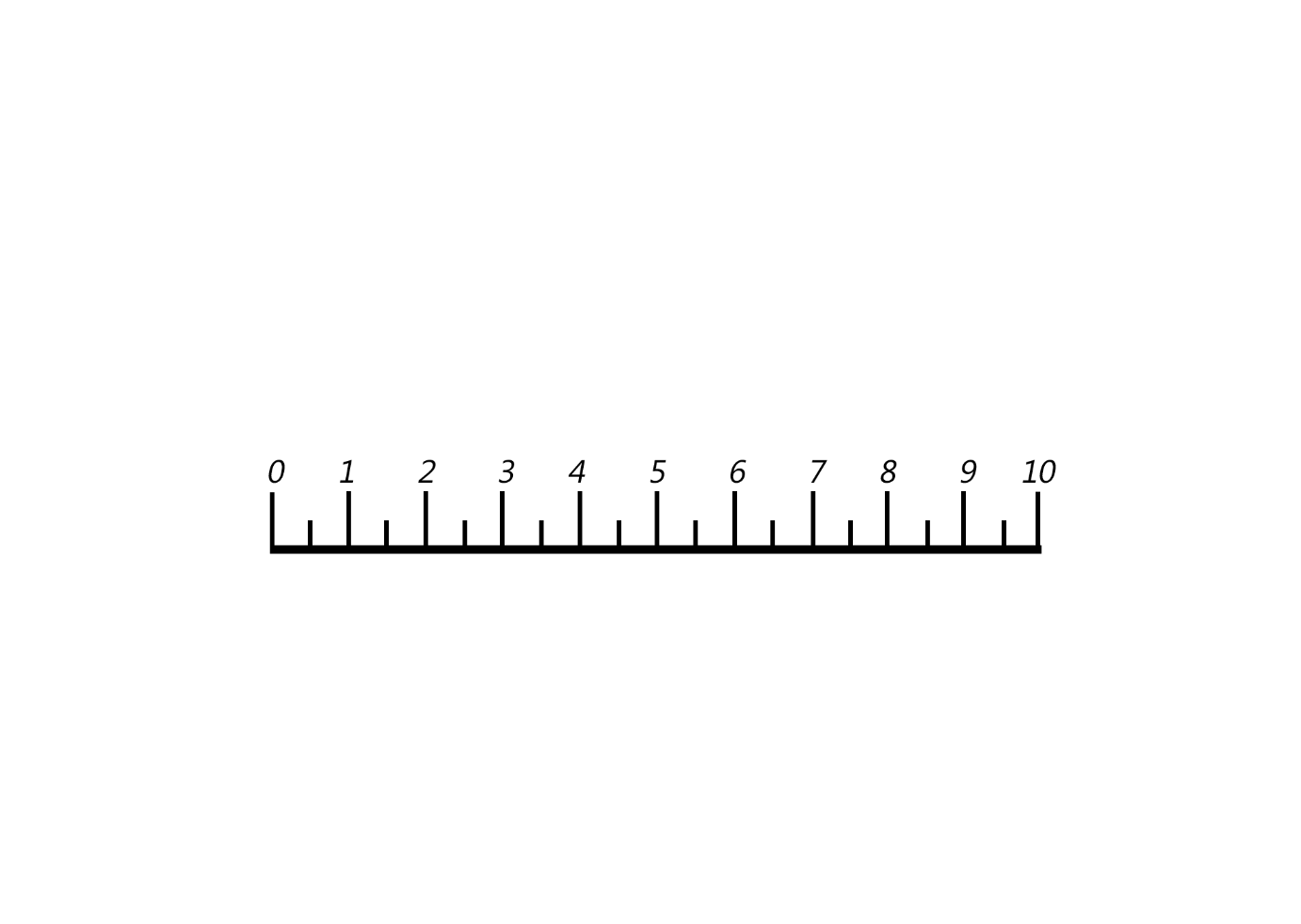


**Irritabilità/Labilità emotiva**

La persona con demenza:

1. è facilmente irritabile;
2. non accetta alcuna osservazione; reagisce nervosamente a ogni minima richiesta o a ogni riferimento ai suoi deficit cognitivi o alla sua mancata capacità di svolgere determinati compiti;
3. ha un umore molto variabile;
4. manifesta improvvisi o ingiustificati scoppi d’ira.

*Entità del comportamento*


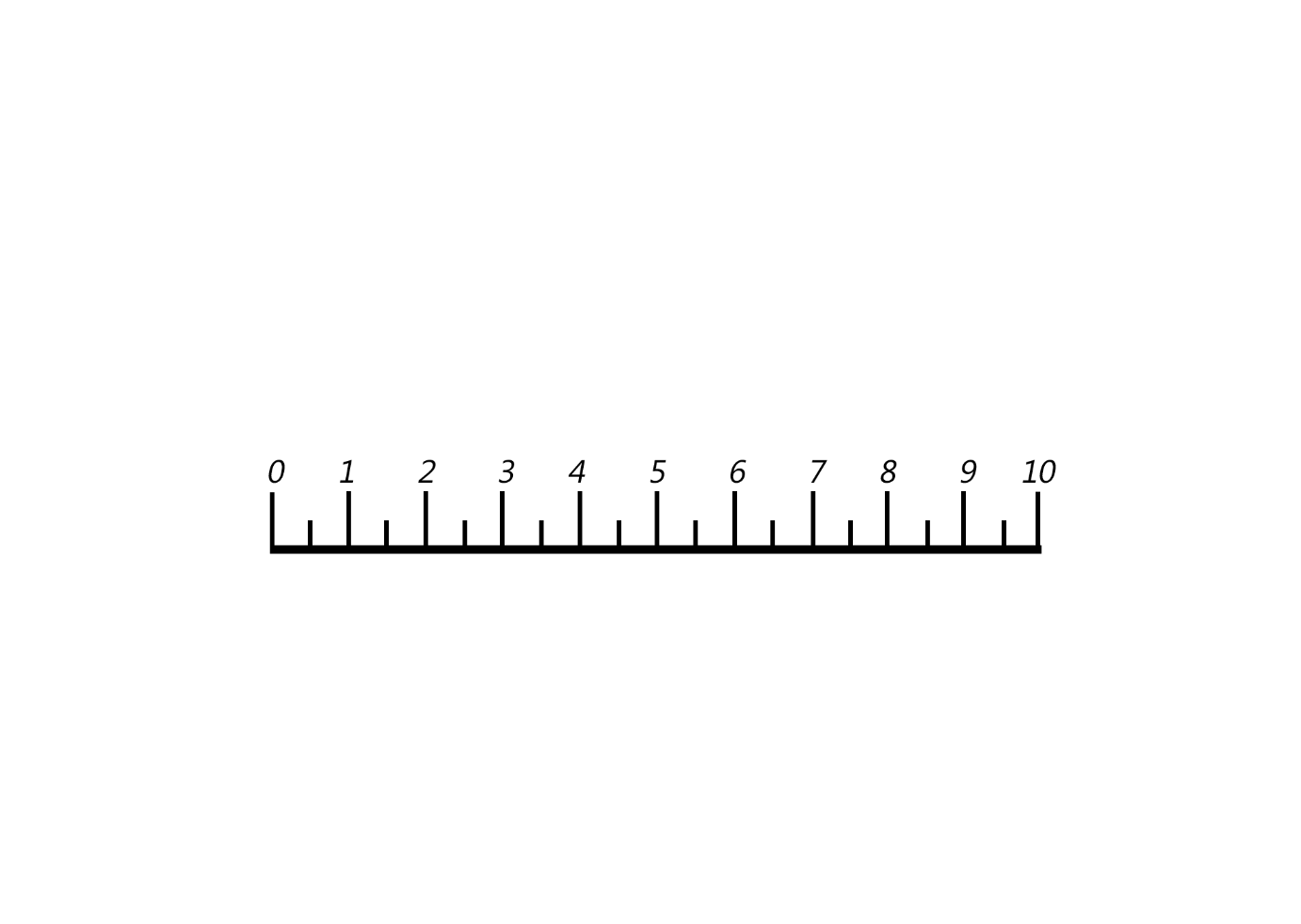


**Deliri**

La persona con demenza:

1. manifesta convinzioni deliranti, ad esempio pensa che qualcuno la stia derubando, oppure nasconda i suoi effetti personali;
2. è convinta che il coniuge la tradisca o che i familiari vogliano abbandonarla;
3. si sente perseguitata o pensa che qualcuno voglia farle del male o avvelenarla;
4. pensa che la casa non sia sua, o che ne esista un’altra identica;
5. ritiene che i familiari siano degli impostori o reagisce con rabbia vedendo la propria immagine nello specchio.

*Entità del comportamento*


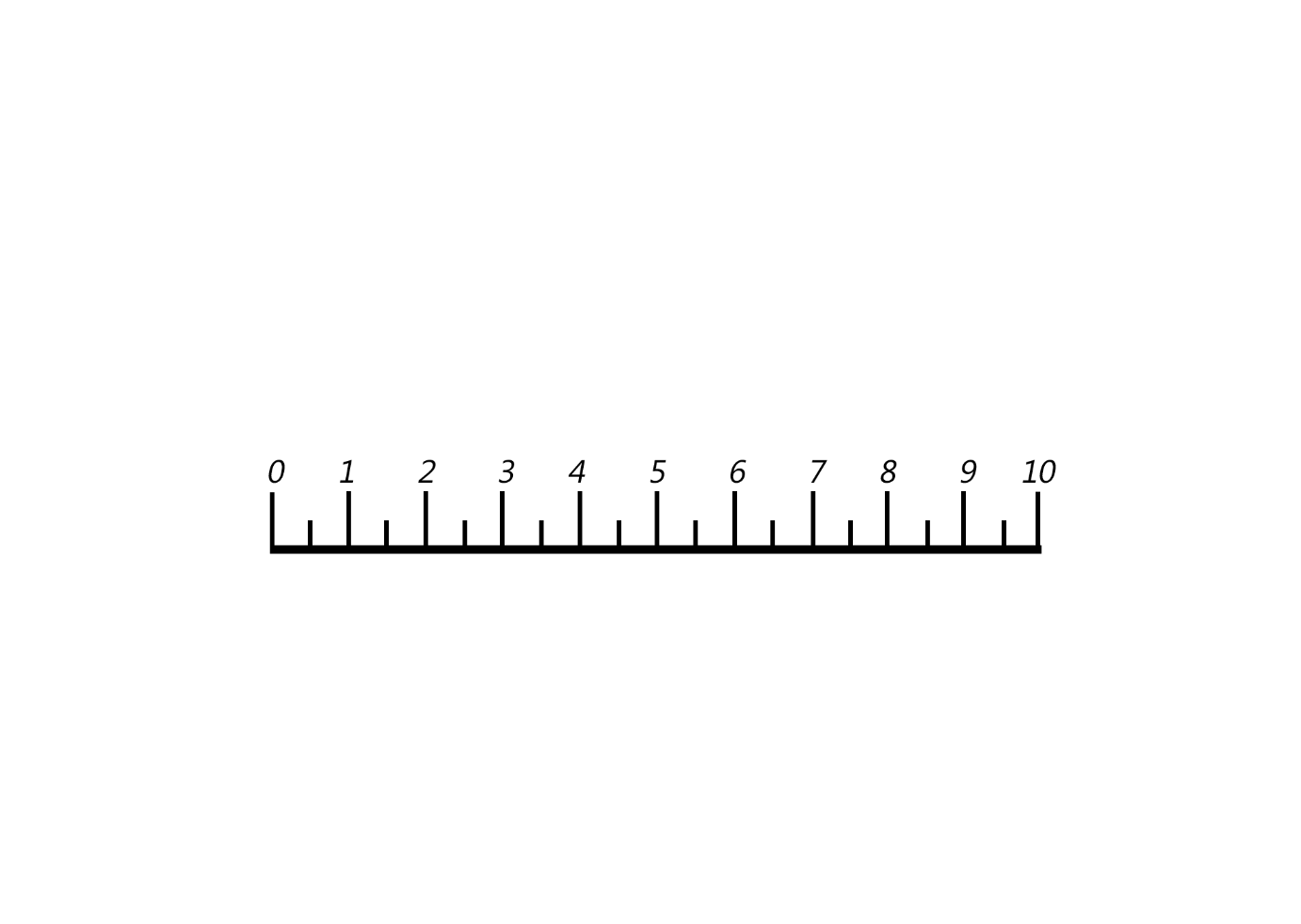


**Allucinazioni**

La persona con demenza:

1. manifesta o si comporta come se avesse allucinazioni visive (ad esempio afferma che vi sono persone inesistenti o animali nella stanza);
2. uditive (ad esempio sente suoni o rumori immaginari);
3. olfattive (riferisce odori o profumi inesistenti);
4. afferma di sentirsi toccare da qualcosa o qualcuno che non esiste, sente strisciare animali sulla pelle (ad es. insetti), si gratta senza una logica spiegazione;
5. lamenta sapori o sensazioni viscerali (ad esempio dolore addominale) senza una causa plausibile;
6. manifesta percezioni sensoriali distorte.

*Entità del comportamento*


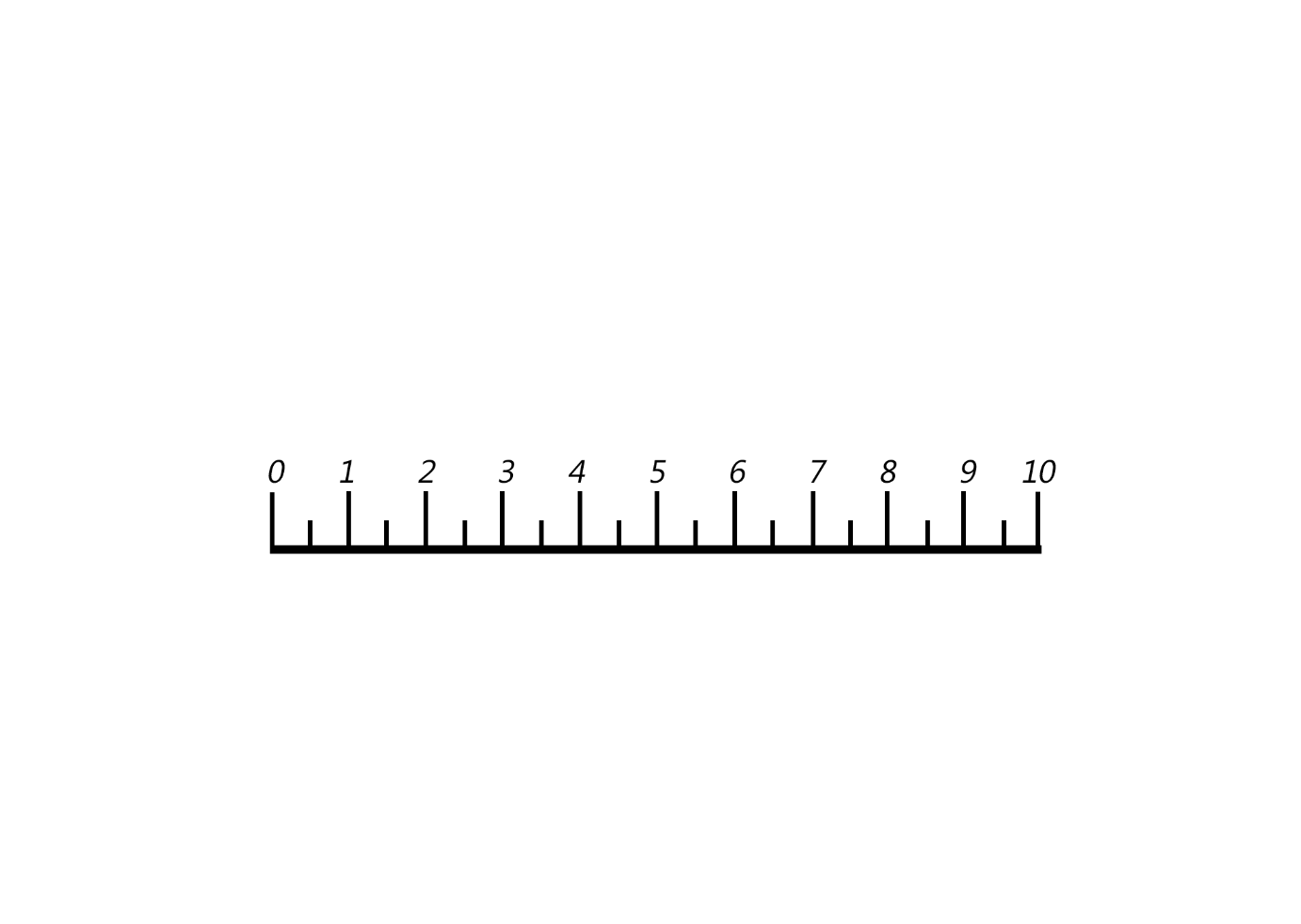


**Euforia**

La persona con demenza:

1. mostra eccessiva o inappropriata allegria durante la visita/attività, si mostra euforica, giocherellona, fa battute stupide;
2. si prende gioco del personale sanitario o delle altre persone presenti anche a rischio di offenderle;
3. ride senza motivo;
4. trova divertenti o ridicole anche cose che in realtà non lo sono o reagisce con allegria quando si parla di argomenti tristi.

*Entità del comportamento*


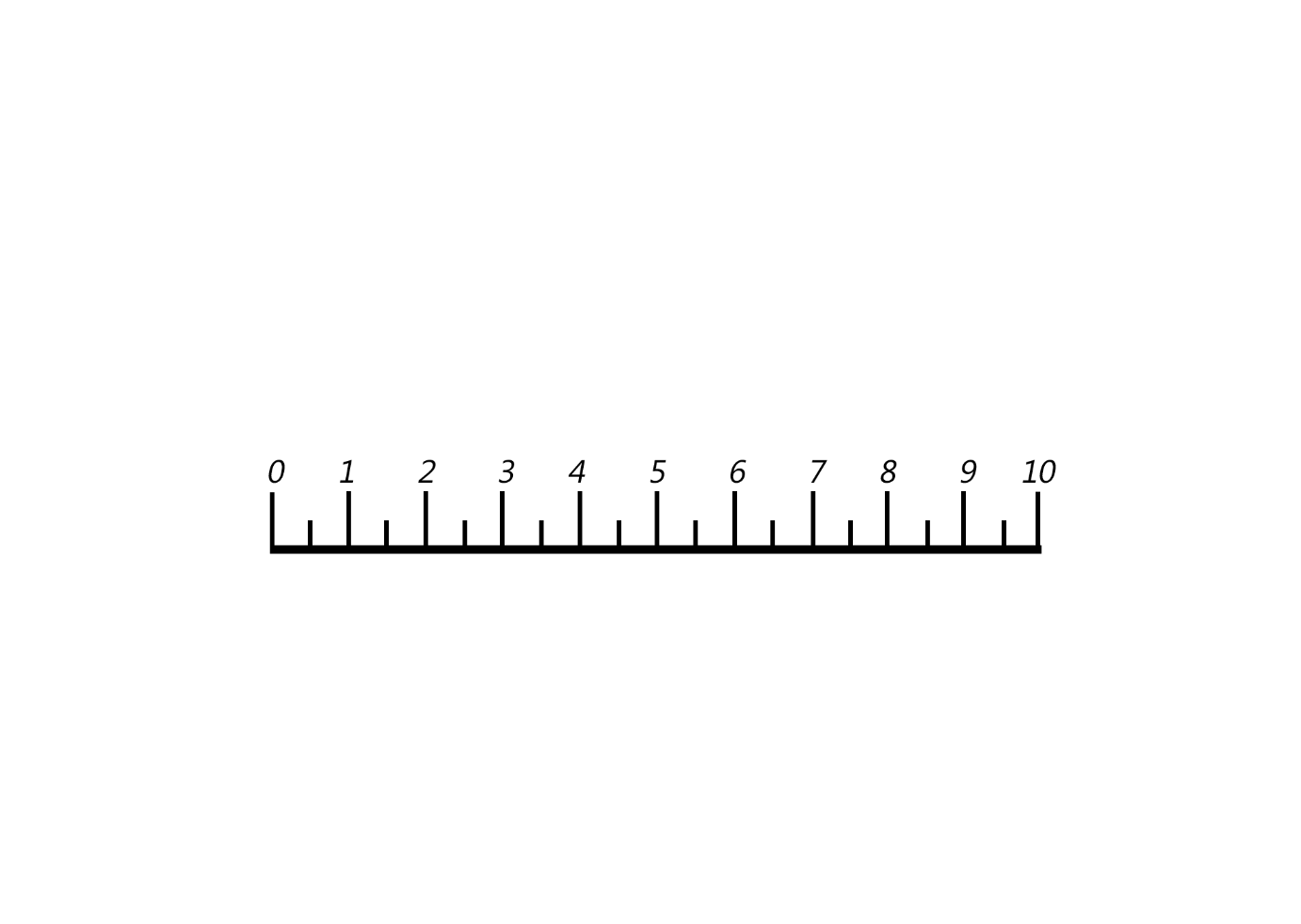


**Disinibizione**

La persona con demenza:

1. si comporta in maniera impulsiva o disinibita, fa cose imbarazzanti;
2. usa un linguaggio volgare, non rispetta le convenzioni sociali, si comporta in maniera maleducata;
3. parla al personale sanitario in maniera eccessivamente confidenziale;
4. fa avance sessuali, si spoglia, cerca di toccare o palpare i presenti o si manipola i genitali durante la visita/attività.

*Entità del comportamento*


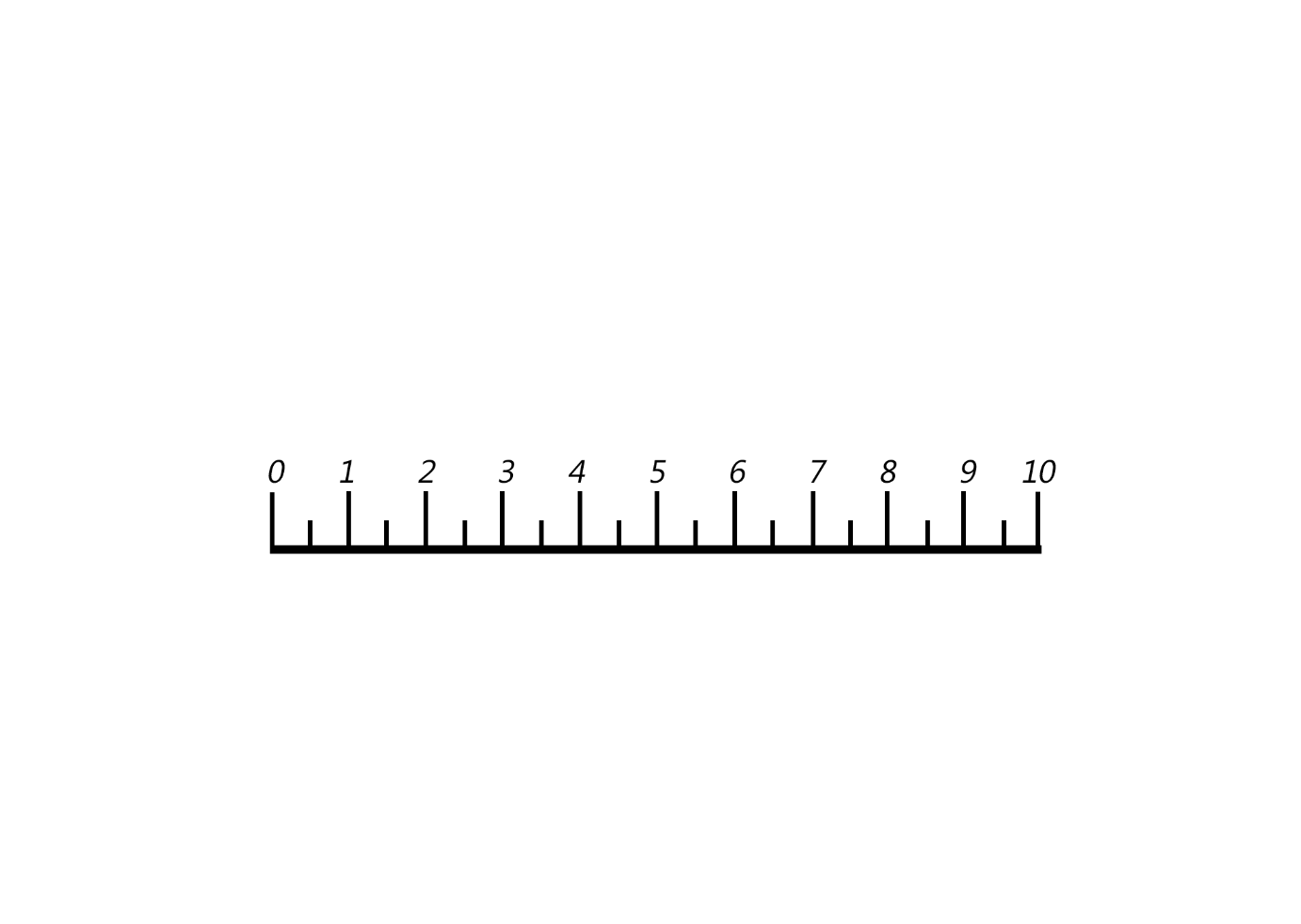


**Disturbi del ritmo sonno/veglia**

La persona con demenza:

1. presenta fluttuazioni della vigilanza, in alcuni momenti della visita o dell’attività sembra “imbambolata”;
2. tende ad addormentarsi durante la visita/attività, deve essere stimolata per stare sveglia.

*Entità del comportamento*


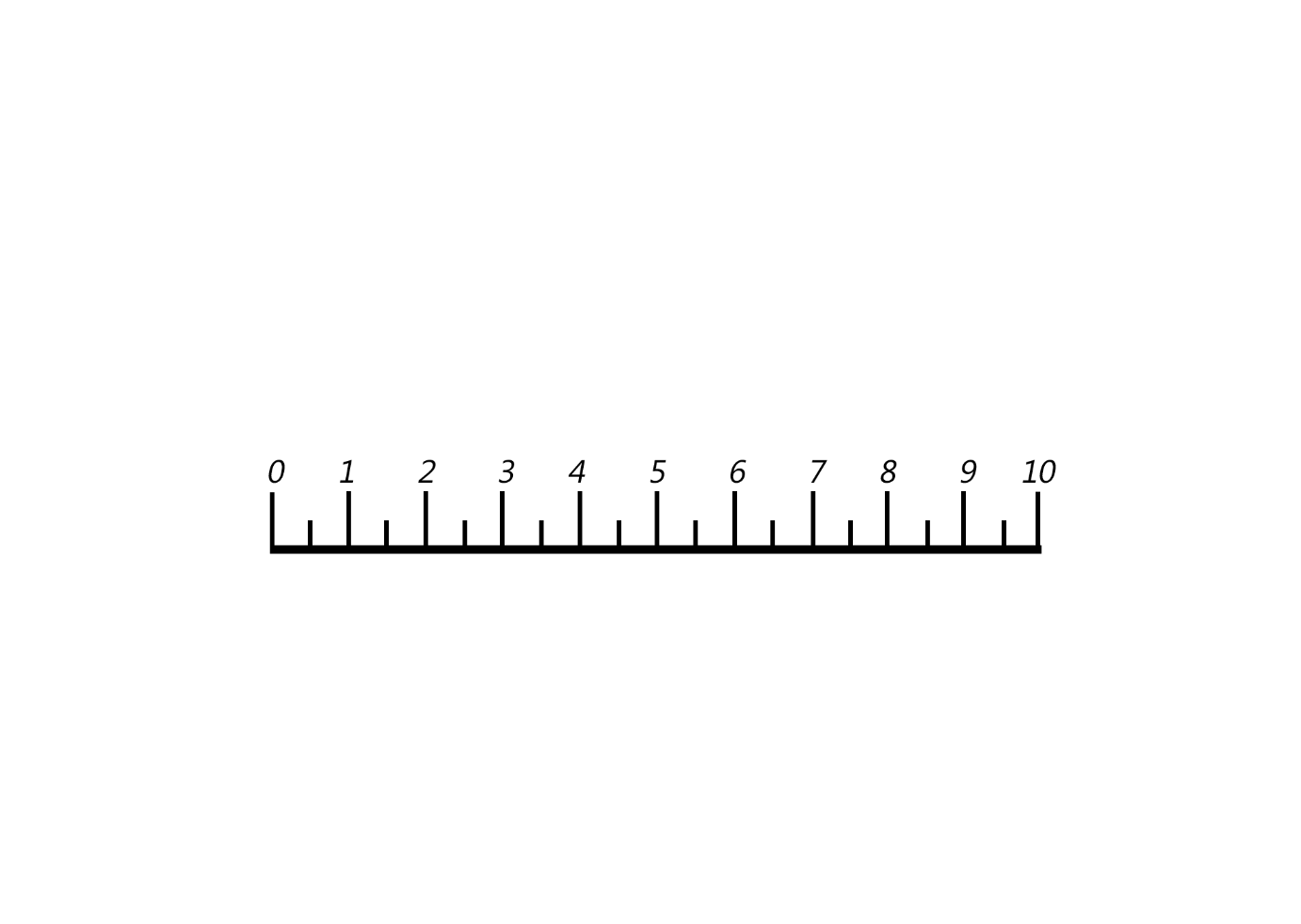


**Ripetitività**

La persona con demenza:

1. continua a ripetere le stesse domande durante la visita/attività;
2. chiede in continuazione che giorno è o dove si trova; reitera domande riguardo a scadenze o appuntamenti;
3. torna sugli stessi argomenti in maniera estremamente ripetitiva;
4. chiede in continuazione conferme al caregiver.

*Entità del comportamento*


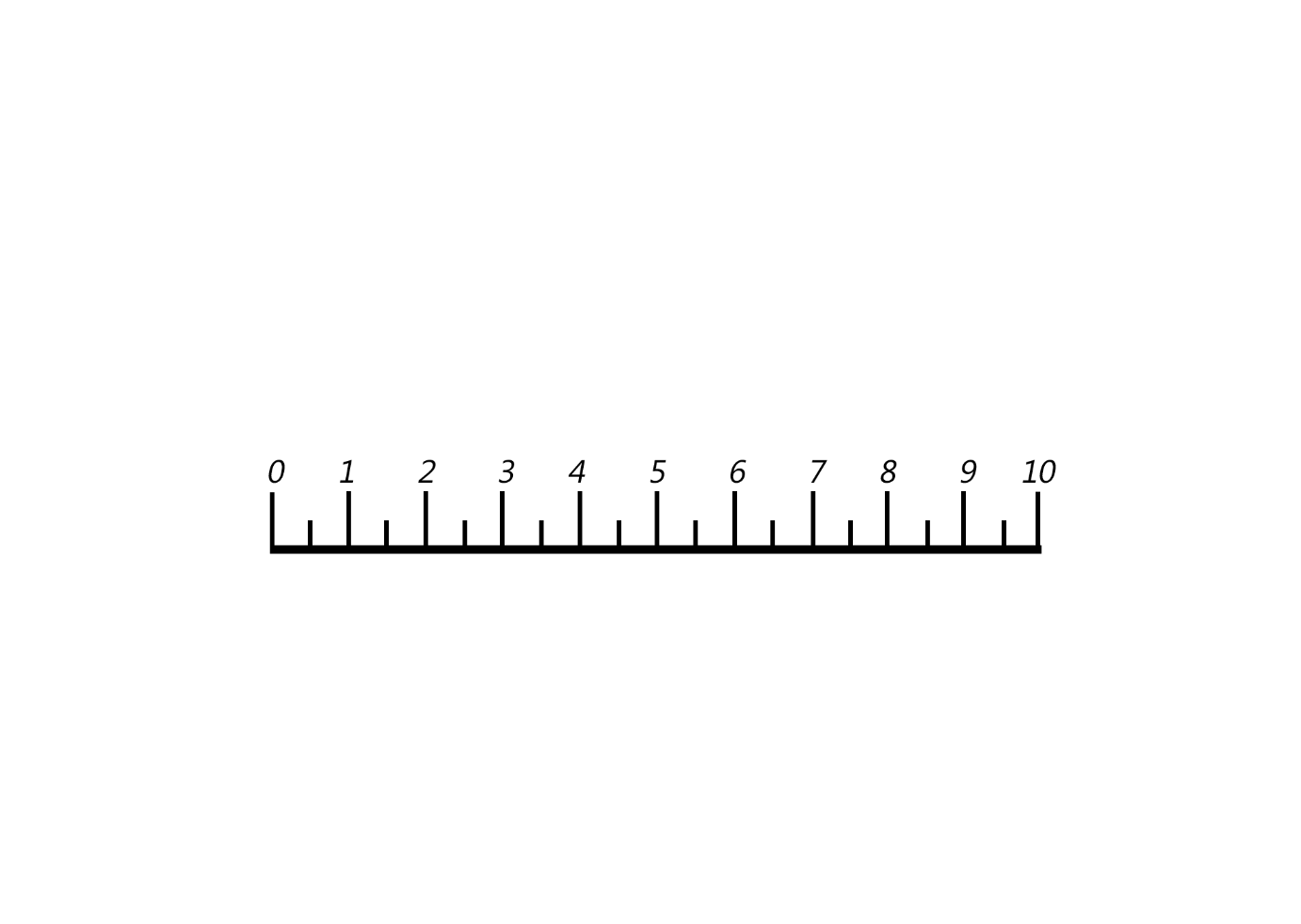


**Disturbi dell’alimentazione**

La persona con demenza:

1. appare chiaramente dimagrita e/o sarcopenica rispetto all’ultima visita;
2. appare avere preso significativamente peso (la valutazione può essere fatta “solamente a occhio” oppure, meglio, con una bilancia);
3. chiede in continuazione di mangiare o di bere o rifiuta di bere se le viene offerto.

*Entità del comportamento*


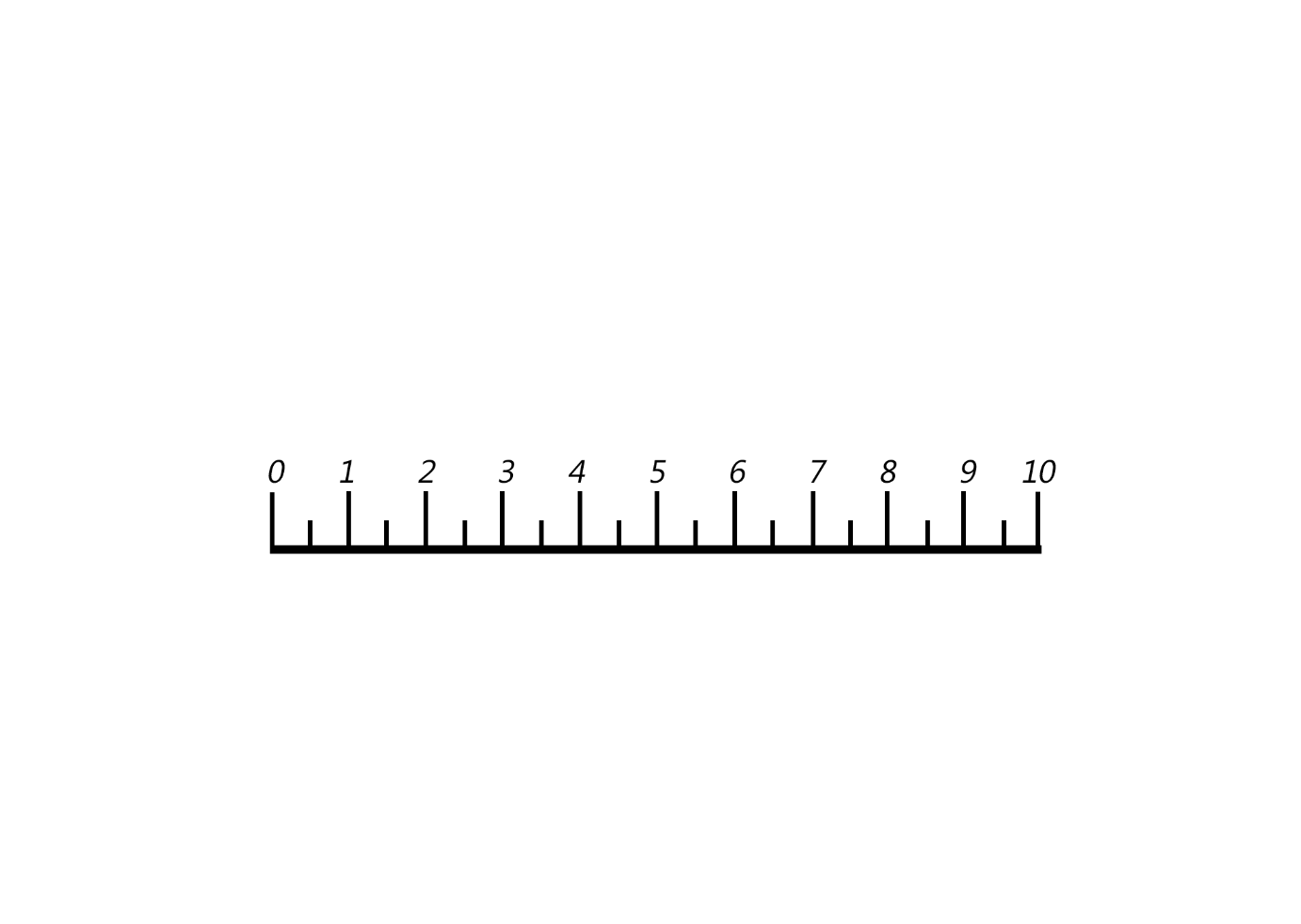


**Variazioni circadiane/stagionali**

La persona con demenza:

1. appare più agitata e confusa nei minuti finali della visita/attività rispetto a quelli iniziali;
2. mostra agitazione o ansia se durante l’attività/visita si modifica il tempo meteorologico.

*Entità del comportamento*


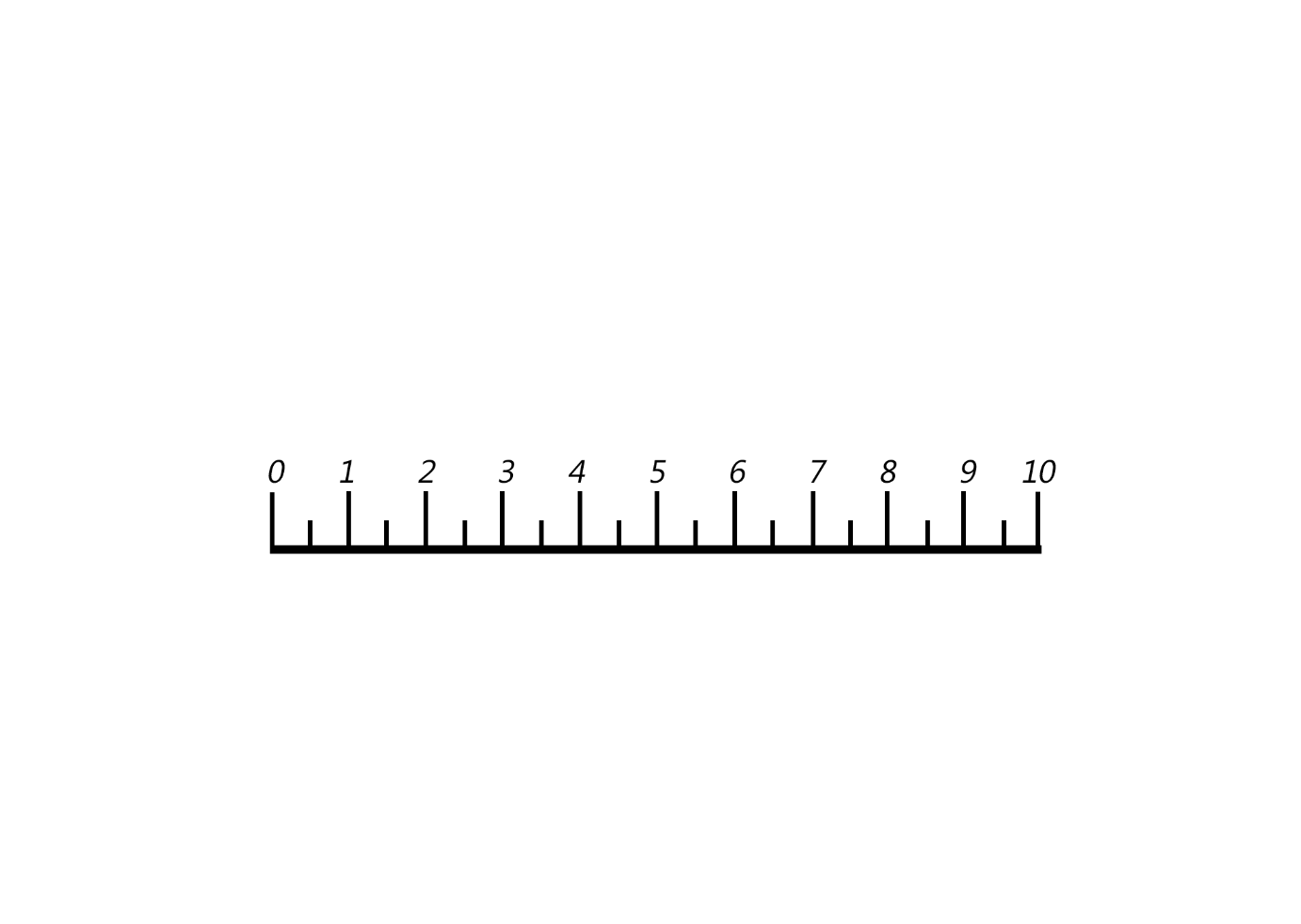


**_____________________________________________________________________**

La ringraziamo per avere risposto alle domande. Le sue risposte miglioreranno la presa in carico della persona con demenza. Per favore risponda anche ad alcune domande che la riguardano e annoti i trattamenti per i BPSD in corso:

Indichi la Sua Età____________Scolarità in anni____________e Sesso  M  F  Non dichiarato

Professione :

- Medico
- Infermiere
- OSS/ASA
- Psicologo
- Tdr
- Educatore
- Psicomotricista
- Ausiliario
- Altro

Aveva già valutato/incontrato la persona con demenza  Sì  No

Trattamenti farmacologici in corso per i BPSD:

NO 

SI 

 neurolettici_____________________________________________

 antidepressivi __________________________________________

 benzodiazepine_________________________________________

 stabilizzanti_____________________________________________

 melatonina_____________________________________________

 integratori______________________________________________

 altro____________________________________________________

Trattamenti non farmacologici in corso per i BPSD:

NO 

SI 

 stimolazione cognitiva__________________________________

 terapia occupazionale/ricreativa________________________

 psicomotricità___________________________________________

 arteterapia___________________________________________

 musicoterapia___________________________________________

 pet therapy______________________________________________

 Gentle care®/Person Centred Care_____________________

 Terapia della bambola__________________________________

 aromaterapia____________________________________________

 altro_____________________________________________________

Durata delle visita/attività odierna in minuti:________________________________________

**Punteggi**

|  |  | **Caregiver entità (C.E.)** | **Caregiver gestione** | **Esaminatore**  **(ES)** | **Totale I e II colonna( C.E.+ E.S.** |
| --- | --- | --- | --- | --- | --- |
| 1 | Apatia |  |  |  |  |
| 2 | Depressione |  |  |  |  |
| 3 | Ansia |  |  |  |  |
| 4 | Compulsioni |  |  |  |  |
| 5 | Agitazione |  |  |  |  |
| 6 | Affaccendamento |  |  |  |  |
| 7 | Aggressività verbale |  |  |  |  |
| 8 | Aggressività fisica |  |  |  |  |
| 9 | Irritabilità/labilità emotiva |  |  |  |  |
| 10 | Deliri |  |  |  |  |
| 11 | Allucinazioni |  |  |  |  |
| 12 | Euforia |  |  |  |  |
| 13 | Disinibizione |  |  |  |  |
| 14 | Disturbi del ritmo sonno/veglia |  |  |  |  |
| 15 | Ripetitività |  |  |  |  |
| 16 | Disturbi dell’alimentazione |  |  |  |  |
| 17 | Variazioni circadiane/stagionali |  |  |  |  |
| **Totale** | |  |  |  |  |
