# Supplementary material for: Italian standardization of the BPSD-SINDEM scale for the assessment of neuropsychiatric symptoms in persons with dementia
Source: Front Neurol. 2024 Nov 21;15:1455787. doi: 10.3389/fneur.2024.1455787 (PMC11617322; doi:10.3389/fneur.2024.1455787)
Supplement: Supplementary file 5 [file Data_Sheet_4.docx]

**SINDEM BEHAVIORAL AND PSYCHOLOGICAL SYMPTOMS DISORDER SCALE (BPSD-SINDEM)**

**CAREGIVER SCALE**

Answer the following question referring to the behavior of the person under your care in the last month. We ask you to rate the behavior on two scales: 1) a scale of behavior extent (considering both frequency and severity) and 2) a scale of your perceived ability to manage that behavior. The first scale ranges from 0 (behavior is not present) to 10 (the most intense behavior you can imagine). Place a mark between 0 and 10 to rate the severity. Please respond even if only one of the described behaviors is present. The second scale ranges from 0 (you do not feel capable of managing the behavior) to 5 (you feel perfectly capable). Place a mark between 0 and 5 to rate your management capabilities. We also ask you to highlight, if possible, in each question, the words describing the behaviors of the person you care for (provide a pencil with an eraser, or if not available, a pen or highlighter).

The person under your care:

1. has lost interest in the world and the people around him/her;
2. no longer takes initiatives, always needs to be prompted to do things;
3. does not care about others’ emotions and has limited emotional reactions.

*Extent of behavior*


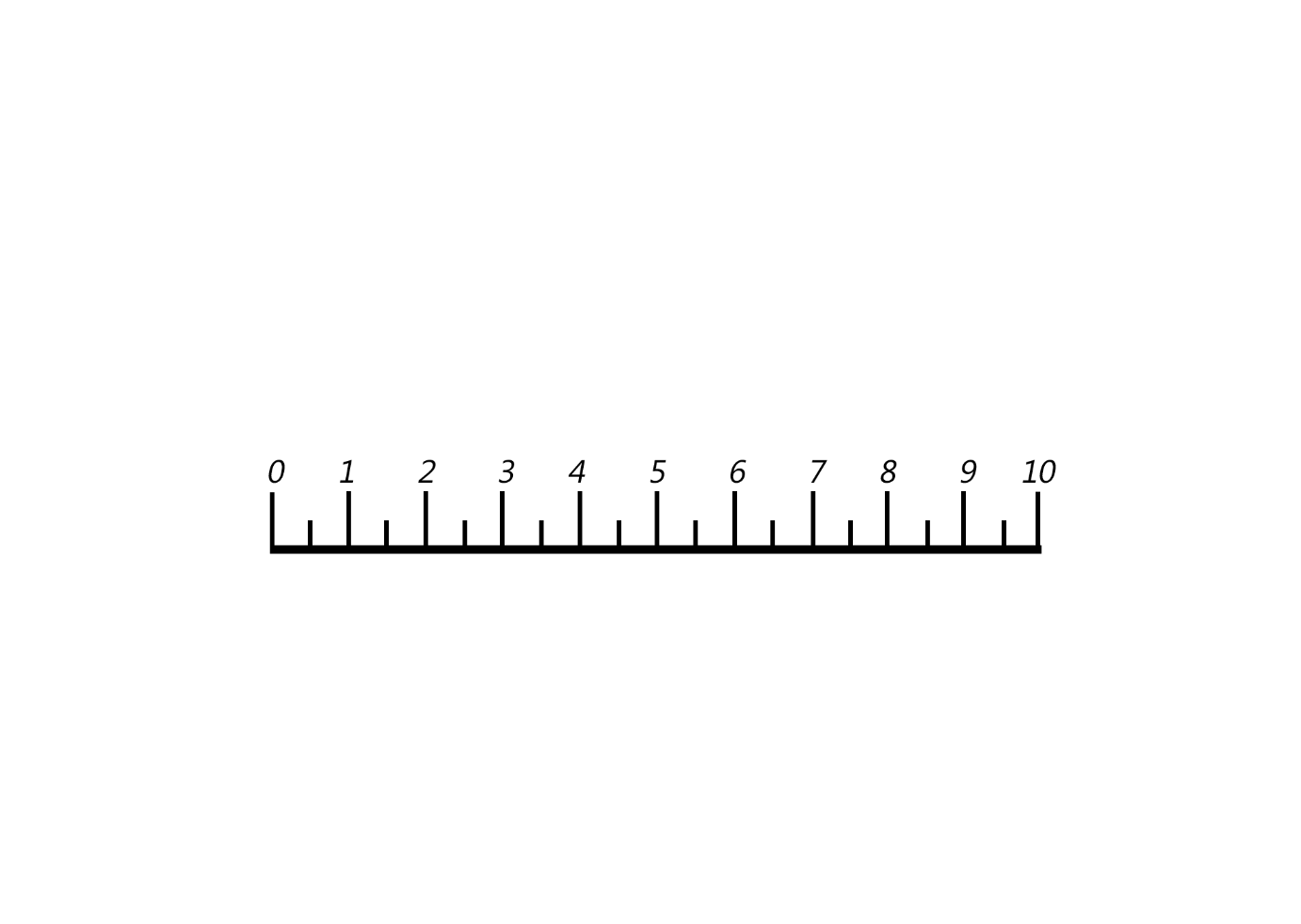


*How capable do I feel of managing this behavior?*


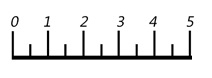


The person under your care:

1. exhibits signs of depression, appears sad, discouraged, hopeless, cries easily;
2. complains of physical discomfort or disturbances that appear not to have a psychological cause;

*Extent of behavior*


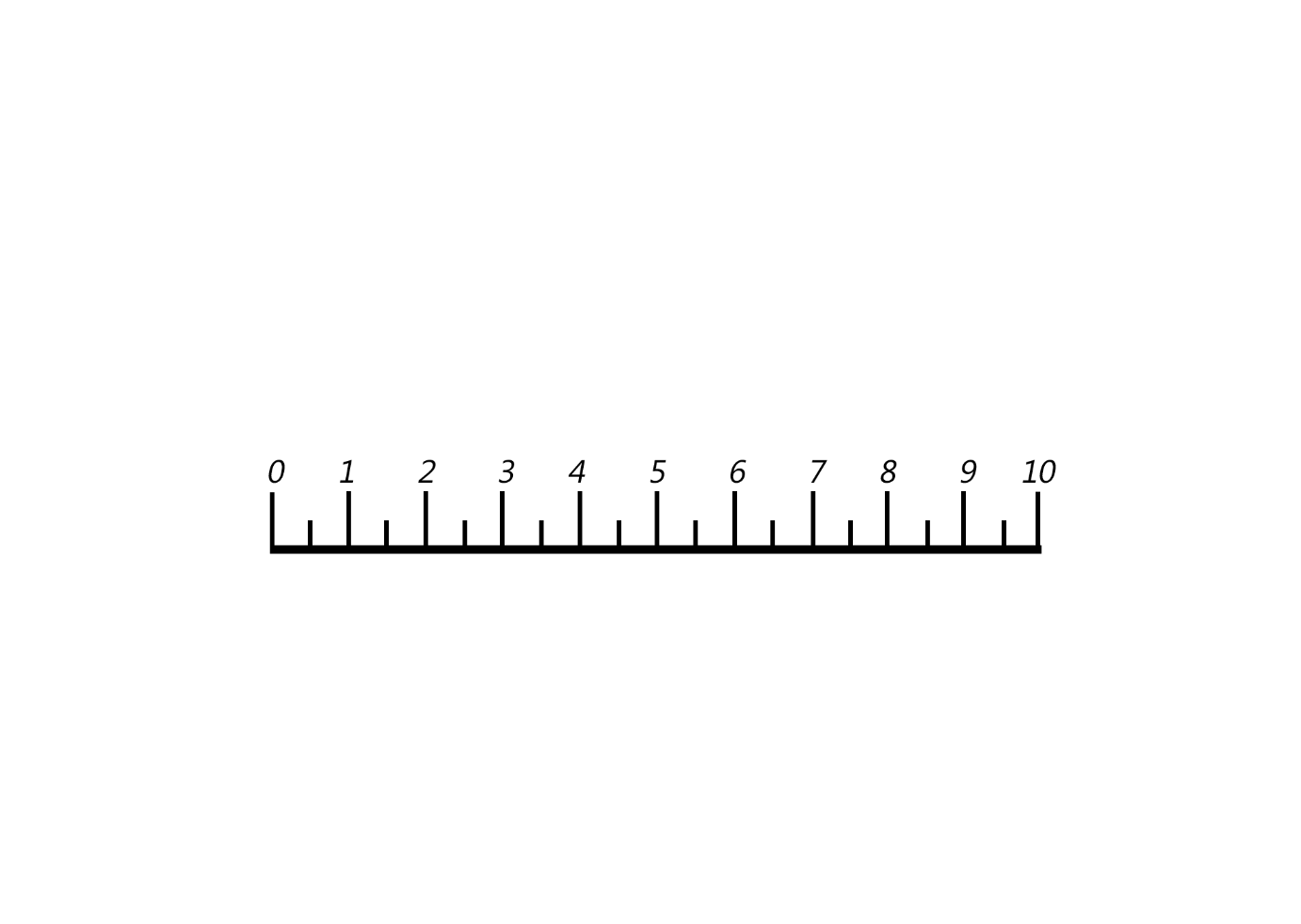


*How capable do I feel of managing this behavior?*


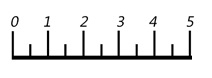


The person under your care:

1. shows anxiety symptoms;
2. is concerned because s/he is far from home;
3. is afraid of running out of money;
4. is afraid of losing memory or his/her health;
5. cannot tolerate waiting;
6. appear tense or worried, especially when confronted with new things;
7. follows you closely like a shadow;

*Extent of behavior*


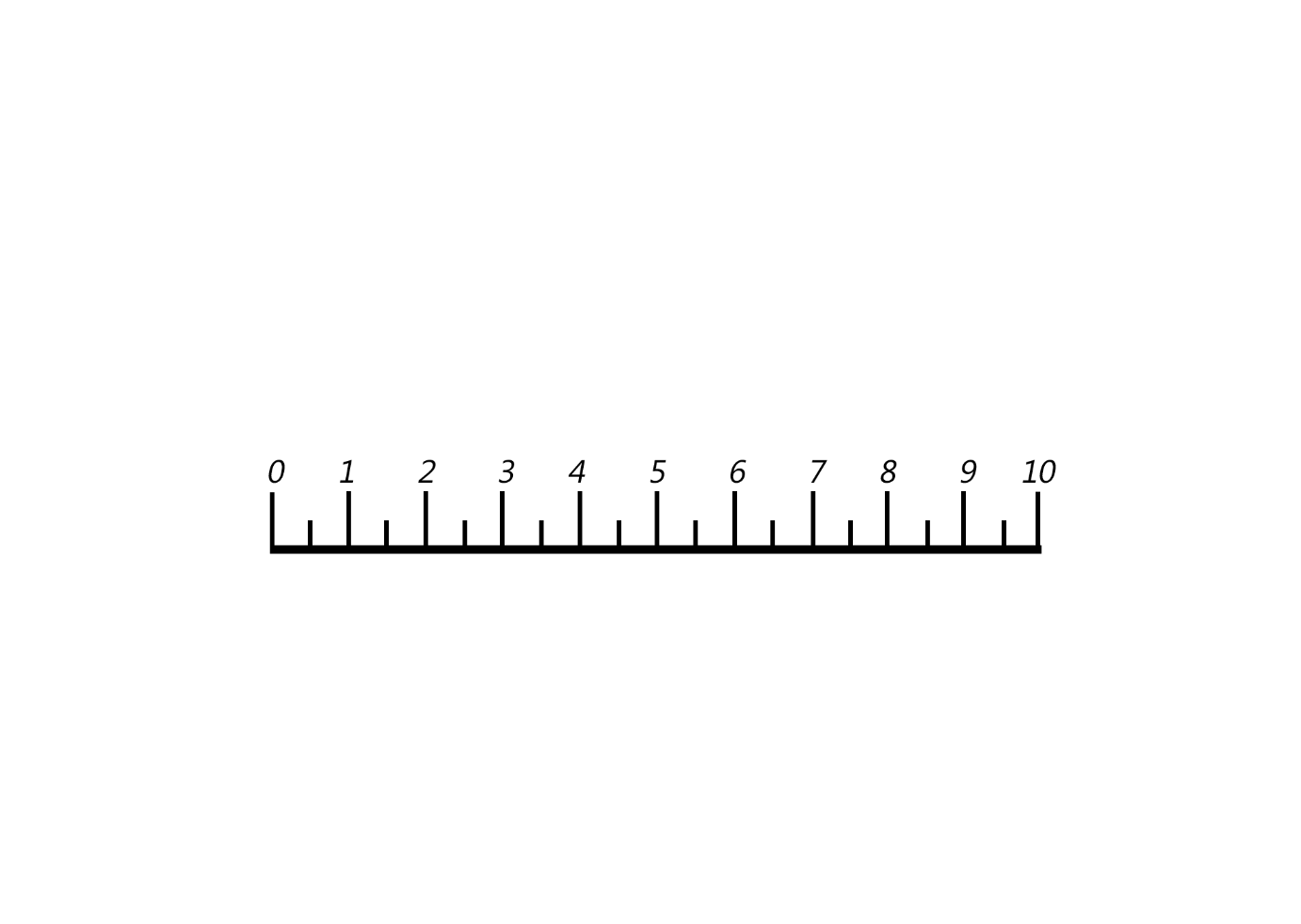


*How capable do I feel of managing this behavior?*


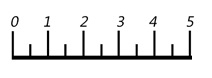


The person under your care:

1. exhibits compulsive behaviors;
2. collect everything found around;
3. puts things found around into his/her mouth;
4. demonstrates an immoderate consumption of tobacco or alcoholic substances.

*Extent of behavior*


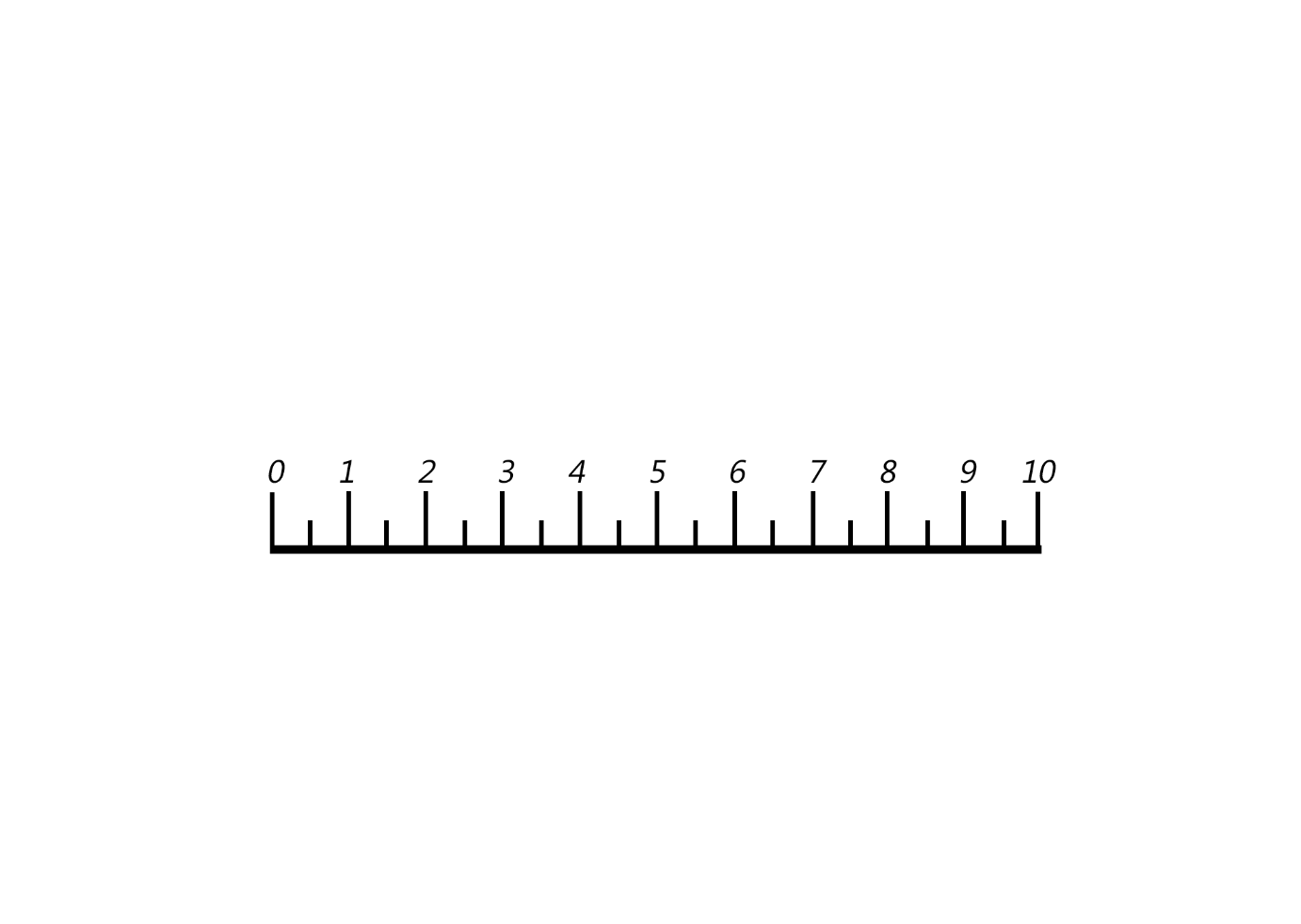


*How capable do I feel of managing this behavior?*


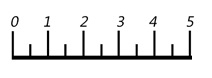


The person under your care:

1. appears agitated and restless, continuously seeks attention by shouting and complaining;
2. attempts to elope from home or the location where she is being cared for;
3. is unable to maintain a seated position;

*Extent of behavior*


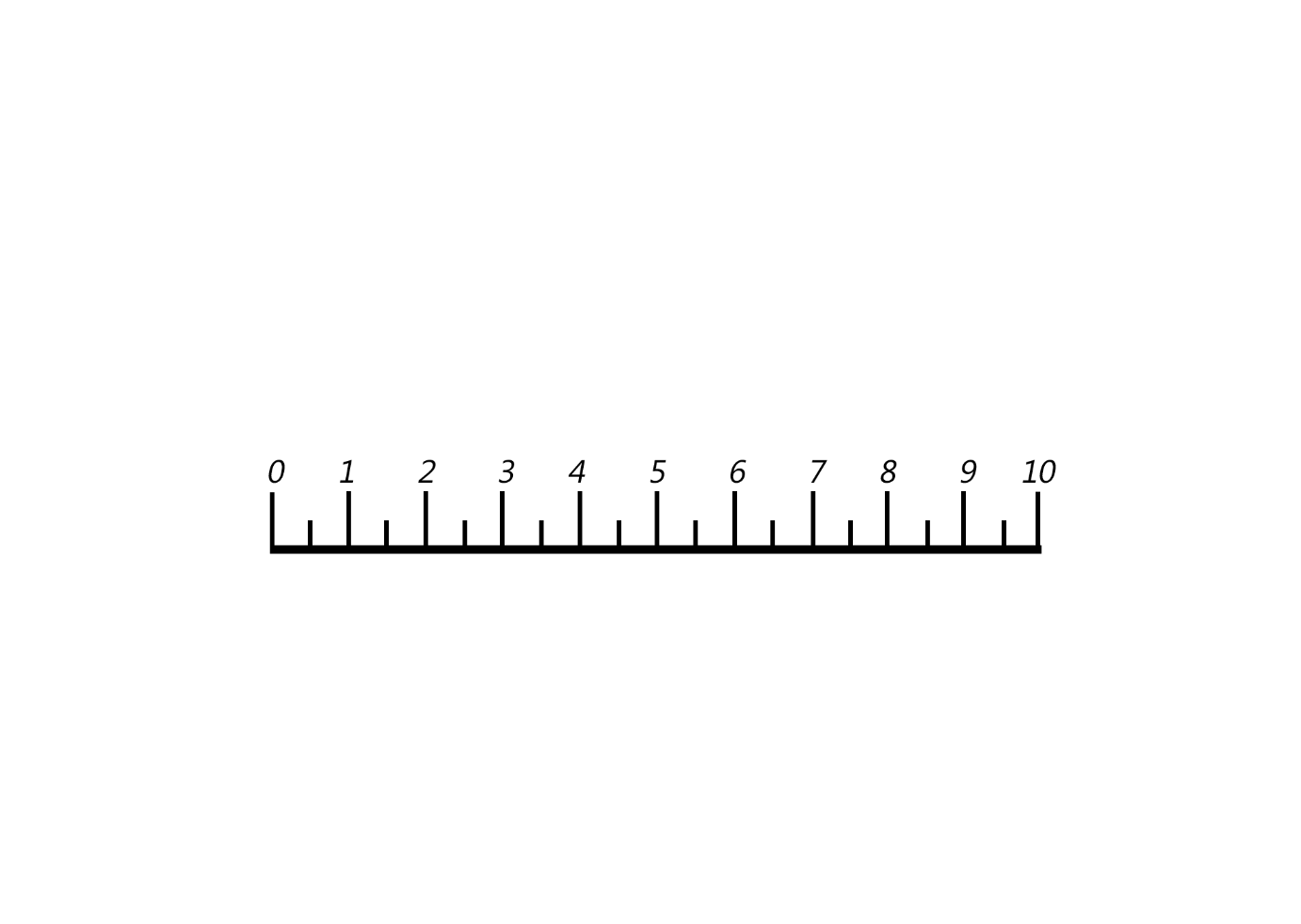


*How capable do I feel of managing this behavior?*


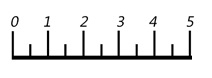


The person under your care:

1. walks around the house with no specific aim, rummaging through objects or closets, concealing personal belongings or money;
2. repeatedly performs specific gestures;
3. keeps putting on and taking off clothes;

*Extent of behavior*


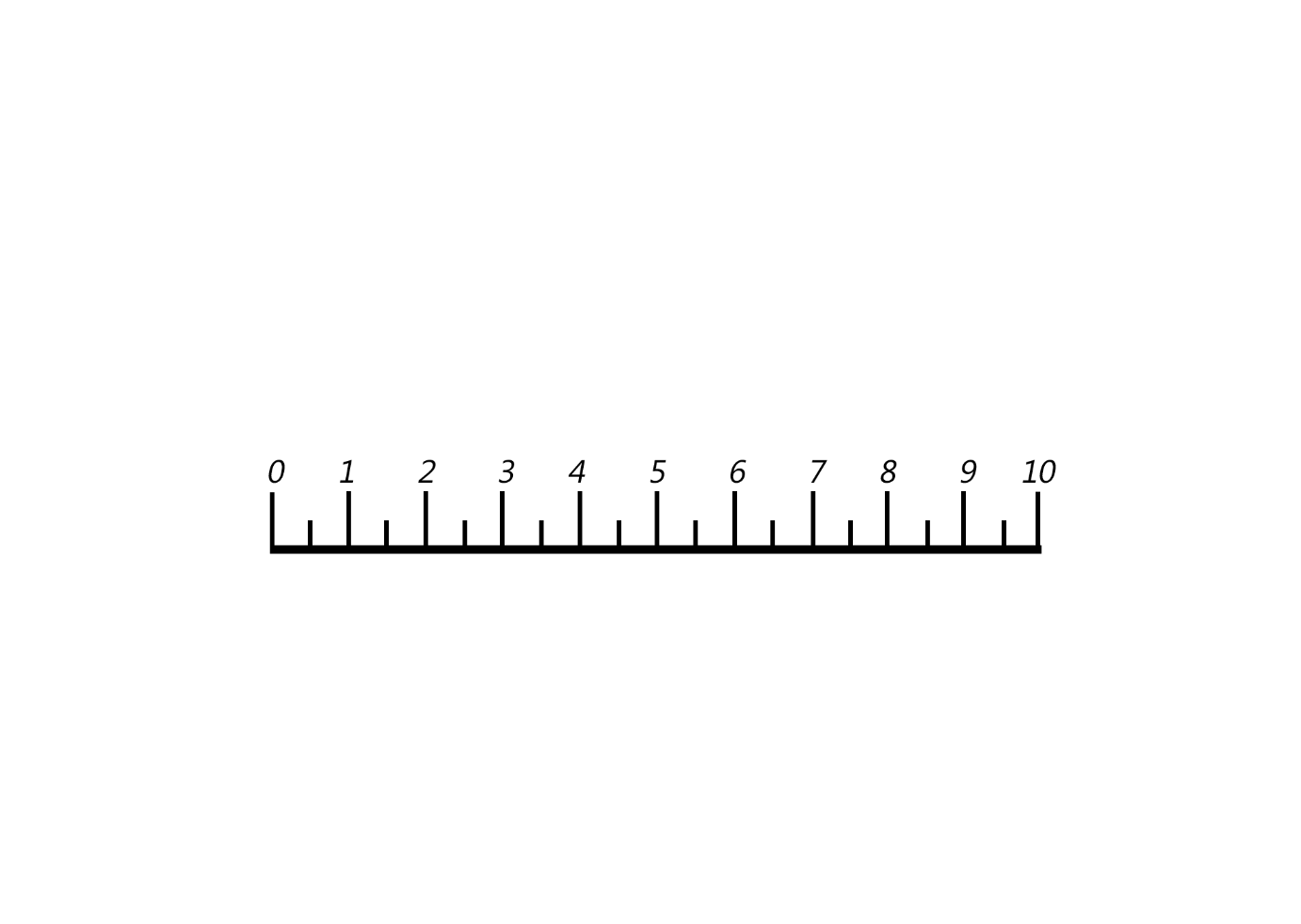


*How capable do I feel of managing this behavior?*


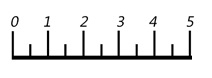


The person under your care:

1. displays verbal aggression and insults others;
2. raises his/her voice or uses an aggressive tone when speaking;

*Extent of behavior*


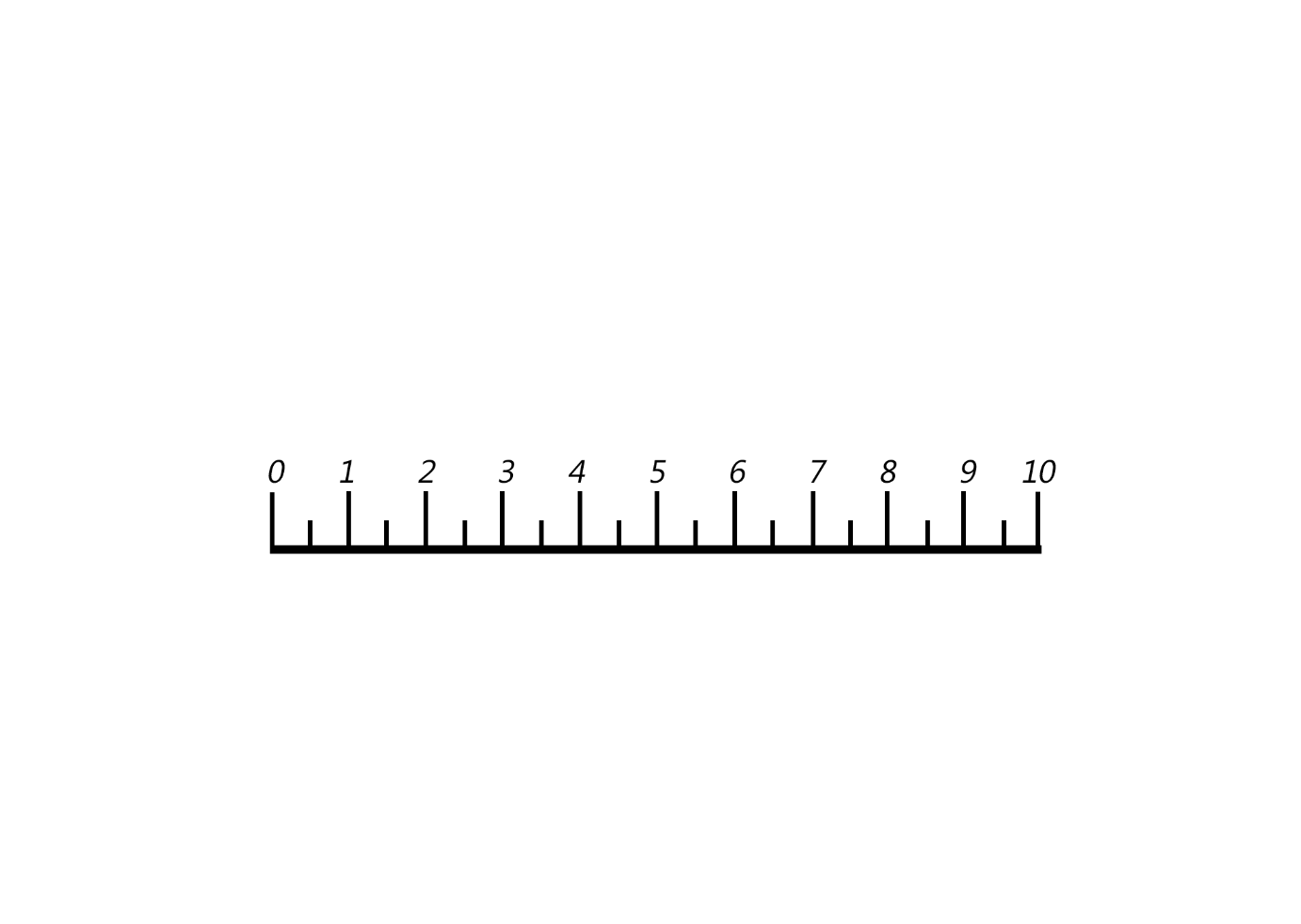


*How capable do I feel of managing this behavior?*


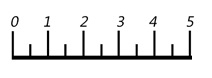


The person under your care:

1. displays physical aggression towards others;
2. destroys objects, damages them, or tears them apart;
3. becomes aggressive or resists certain activities, such as taking the shower or dressing;

*Extent of behavior*


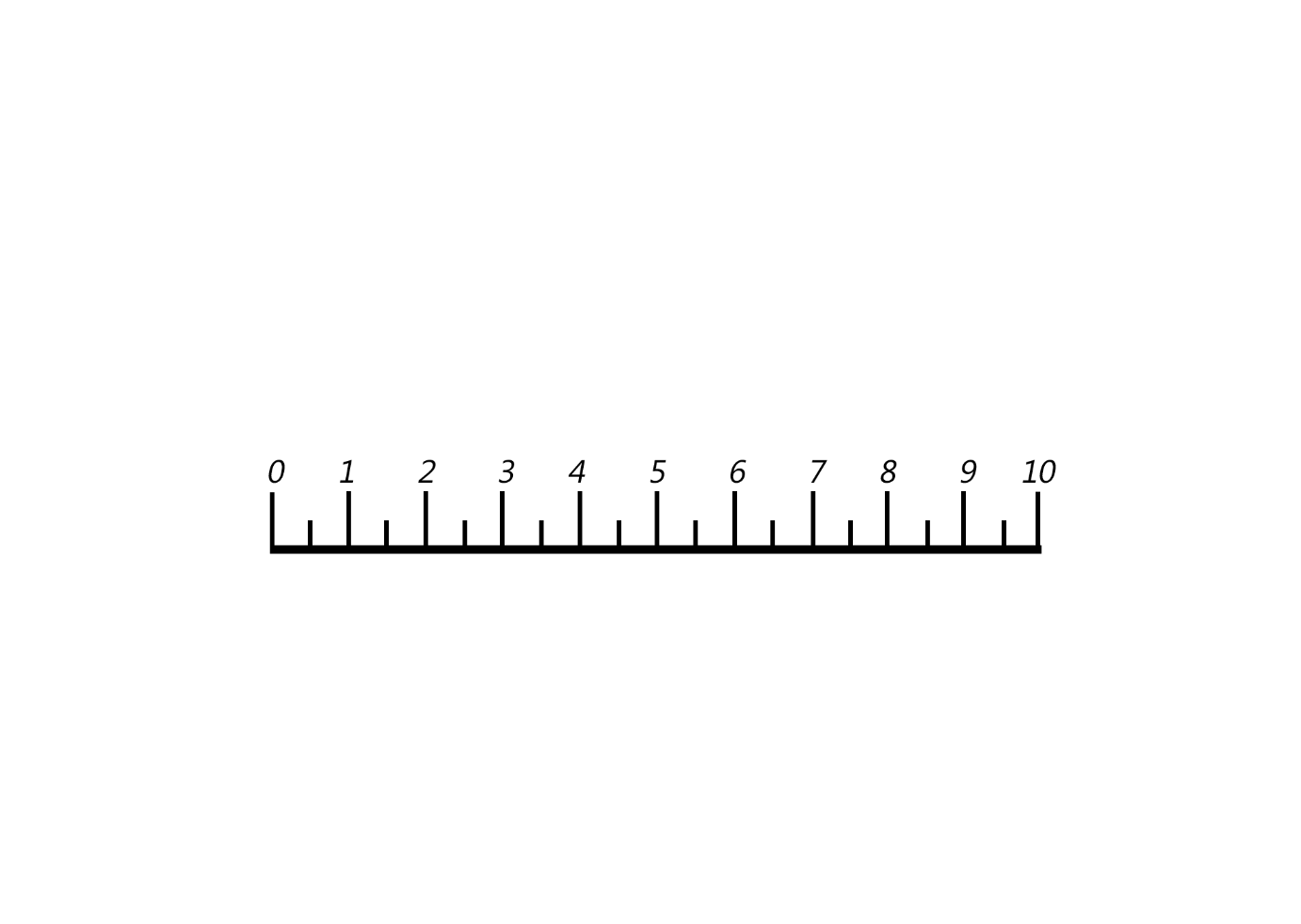


*How capable do I feel of managing this behavior?*


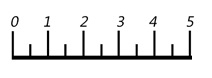


The person under your care:

1. is easily irritable;
2. does not accept any remark;
3. has a highly variable mood swings;
4. exhibits sudden or unjustified outbursts of anger.

*Extent of behavior*


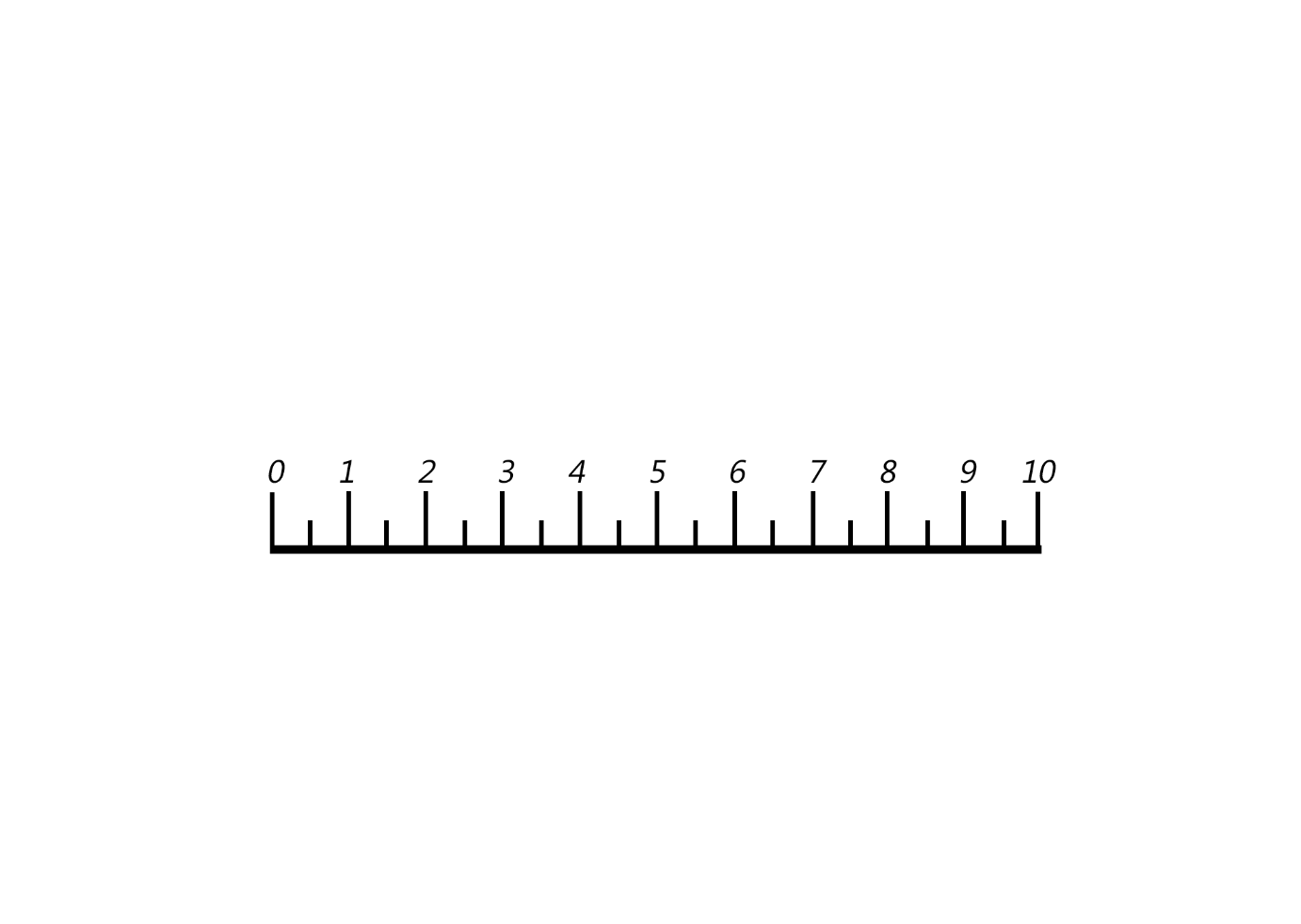


*How capable do I feel of managing this behavior?*


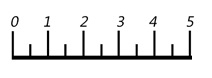


The person under your care:

1. exhibits delusional beliefs, such as thinking that someone is stealing from him/her or hiding his/her personal belongings;
2. is convinced that his/her spouse is cheating on him/her or that family members want to abandon him/her;
3. believes that someone wants to harm him/her or poison him/her;
4. believes that the house is not his/her;
5. interacts with television characters, convinced they are actual individuals or with her own reflection in the mirror;

*Extent of behavior*


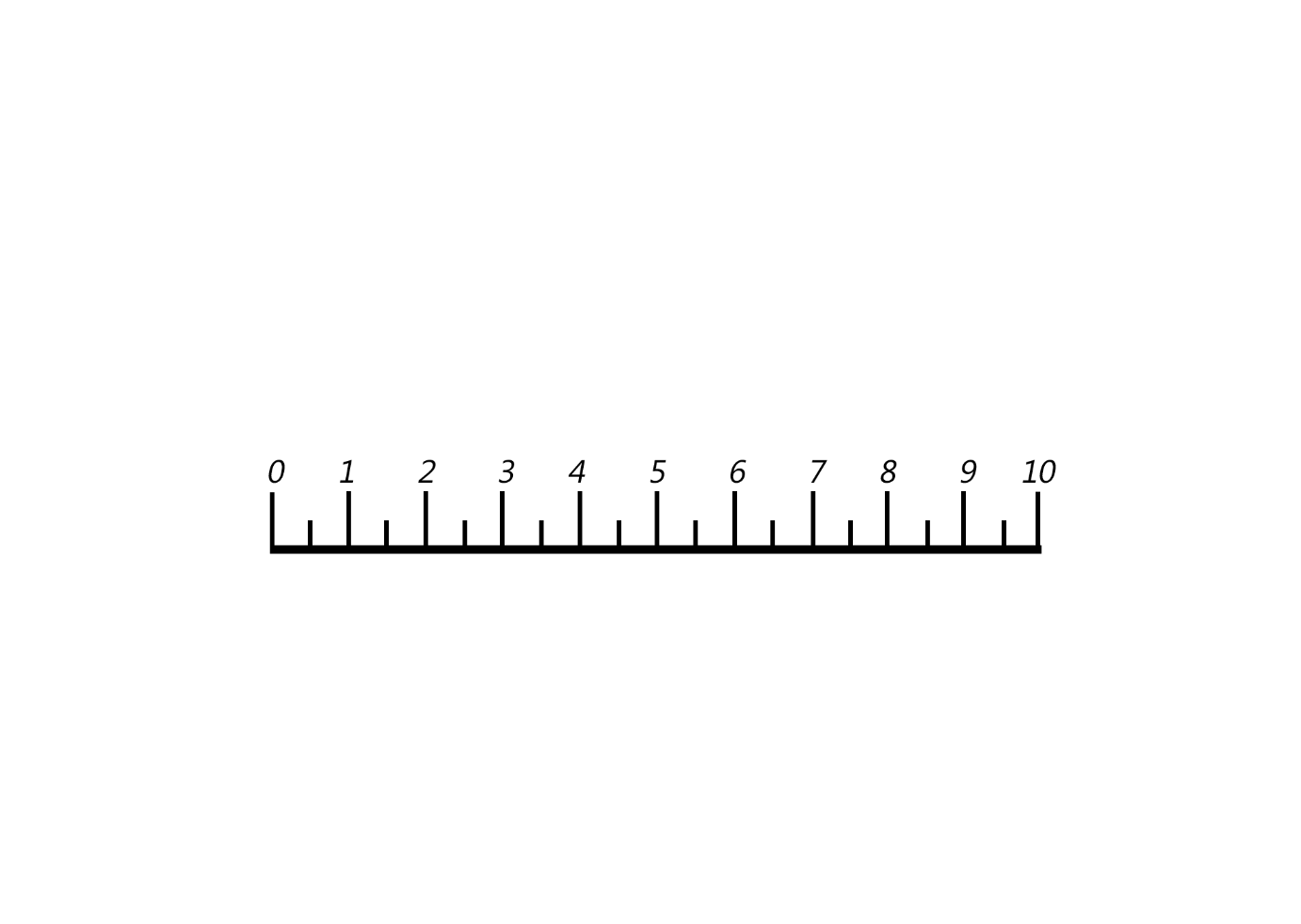


*How capable do I feel of managing this behavior?*


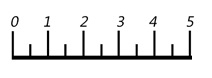


The person under your care:

1. exhibits hallucinations, wherein she/she perceives individuals, animals, or objects that do not exist or behaves as though he/she sees them;
2. experiences auditory hallucinations, perceiving noises, sounds, or music that are non-existent;
3. perceives non-real odors;
4. experiences itching without a logical explanation;

*Extent of behavior*


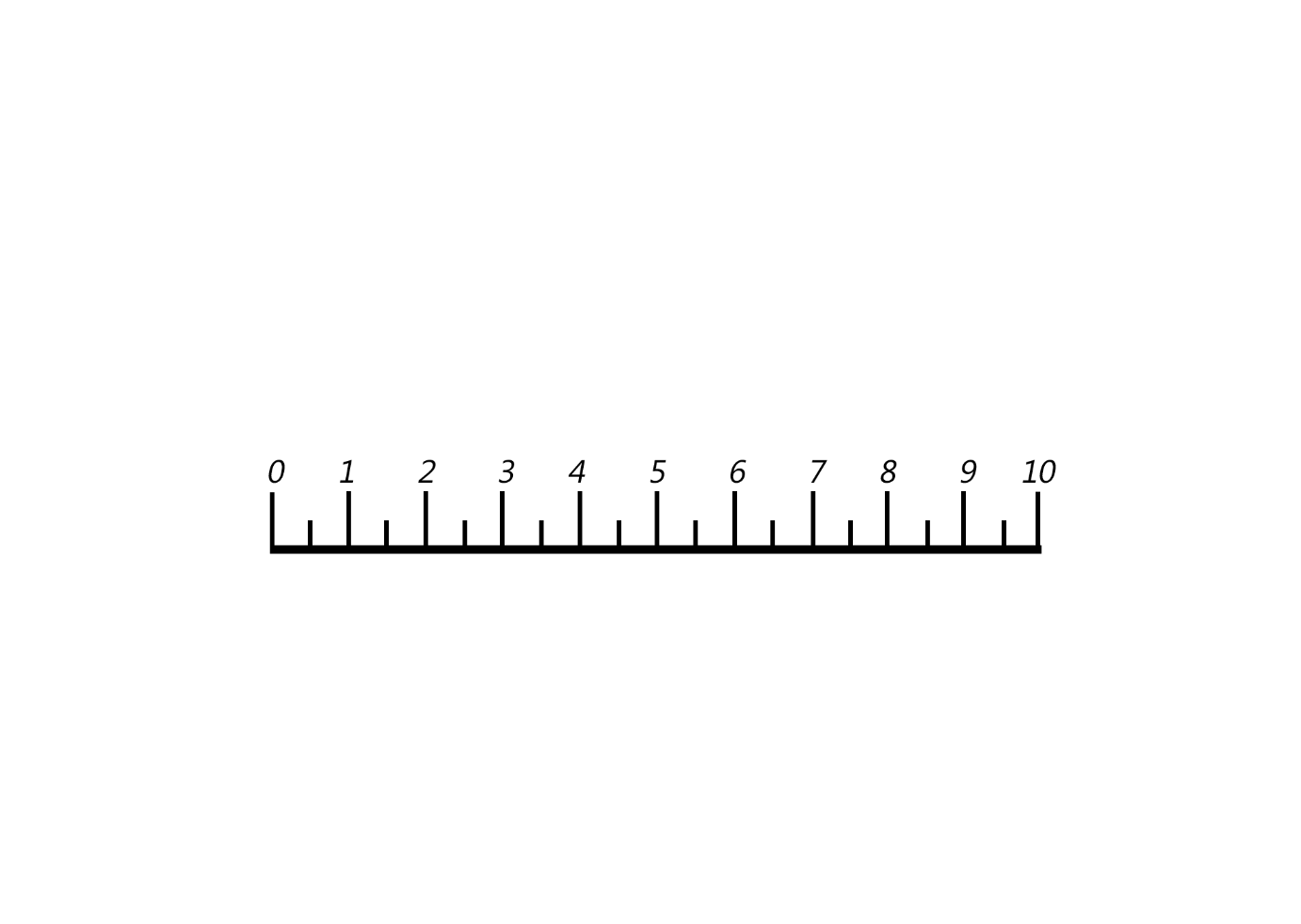


*How capable do I feel of managing this behavior?*


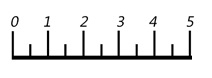


The person under your care:

1. displays excessive or inappropriate cheerfulness in response to life events;
2. engages jokes and laughs without reason;
3. finds things funny or ridiculous that are not so,

*Extent of behavior*


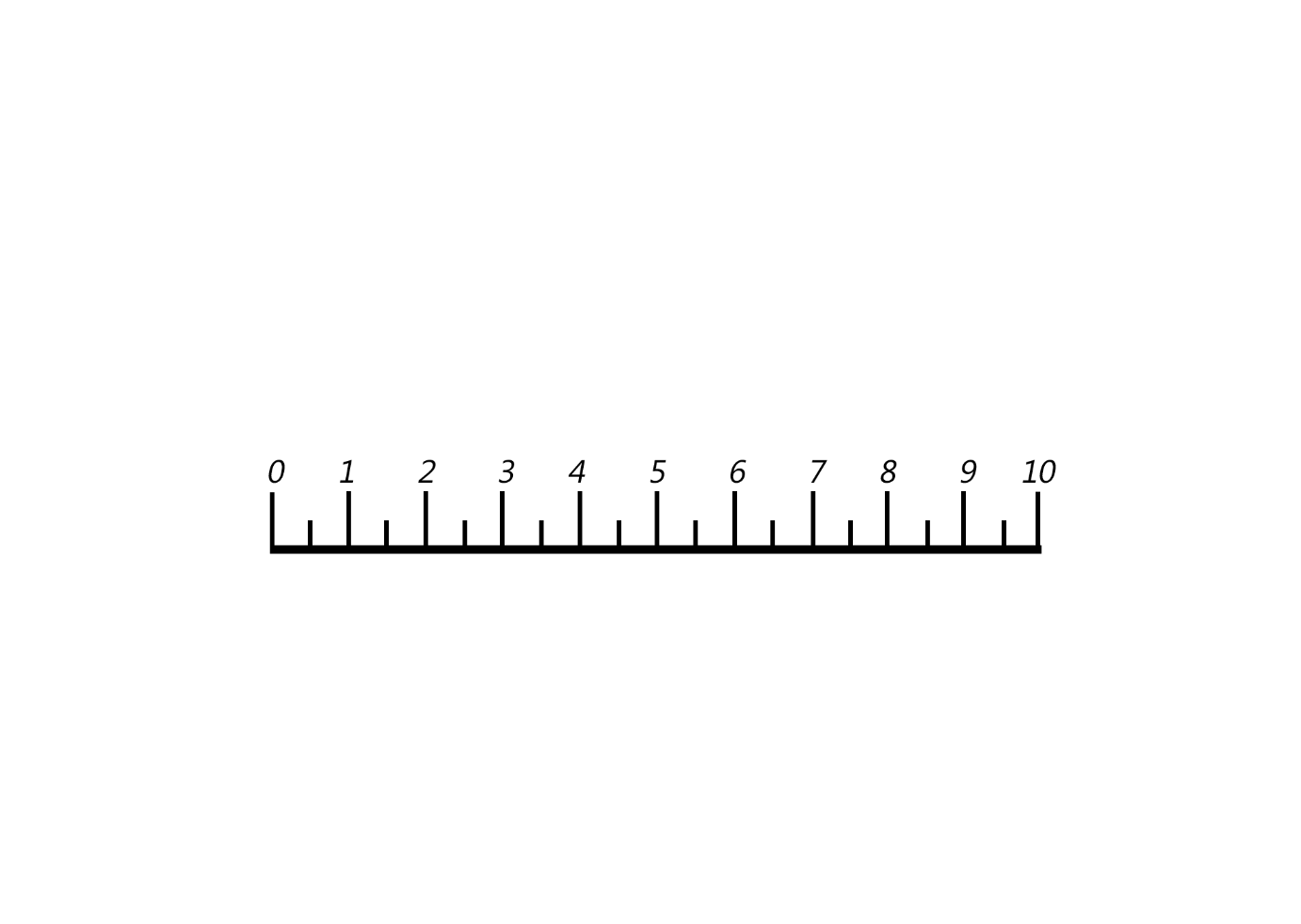


*How capable do I feel of managing this behavior?*


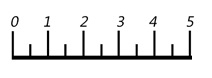


The person under your care:

1. behaves impulsively or uninhibitedly, does embarrassing things;
2. disregards social conventions;
3. speaks to strangers in an excessively familiar manner;
4. makes sexual advances, undresses in public;
5. demands sexual favors from the partner;
6. incessantly talks without listening to responses;

*Extent of behavior*


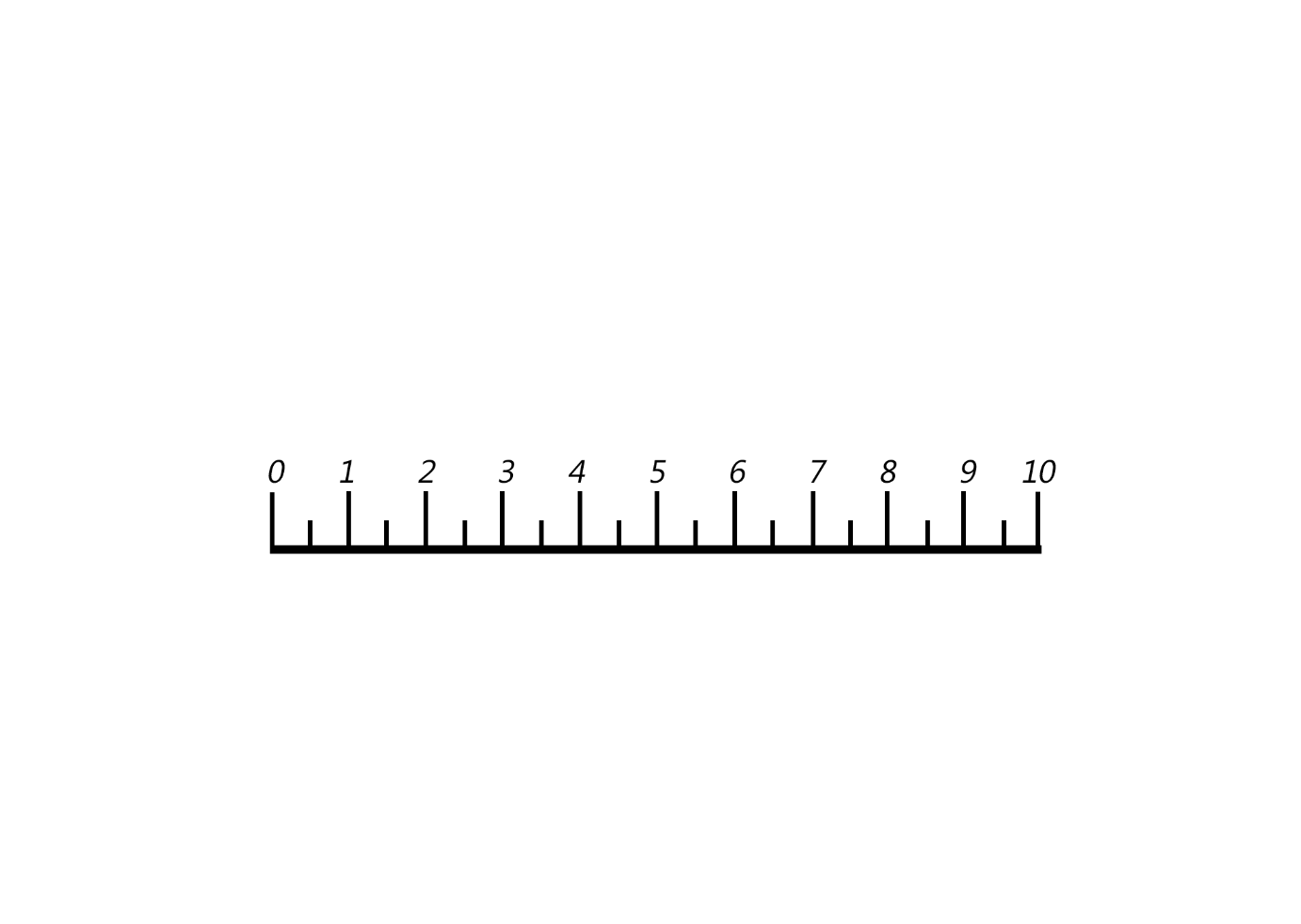


*How capable do I feel of managing this behavior?*


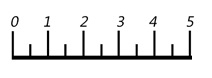


The person under your care

1. struggles with initiating sleep;
2. awakens during the night and fails to resume sleep;
3. wanders around the house at night, searching for food in the refrigerator;
4. wakes up very early in the morning;
5. insist on leaving the house at incongruous hours;
6. is sleepy during the day and takes multiple daytime naps;
7. talks, shouts, becomes restless, or exhibits punching or kicking behaviors during sleep.

*Extent of behavior*


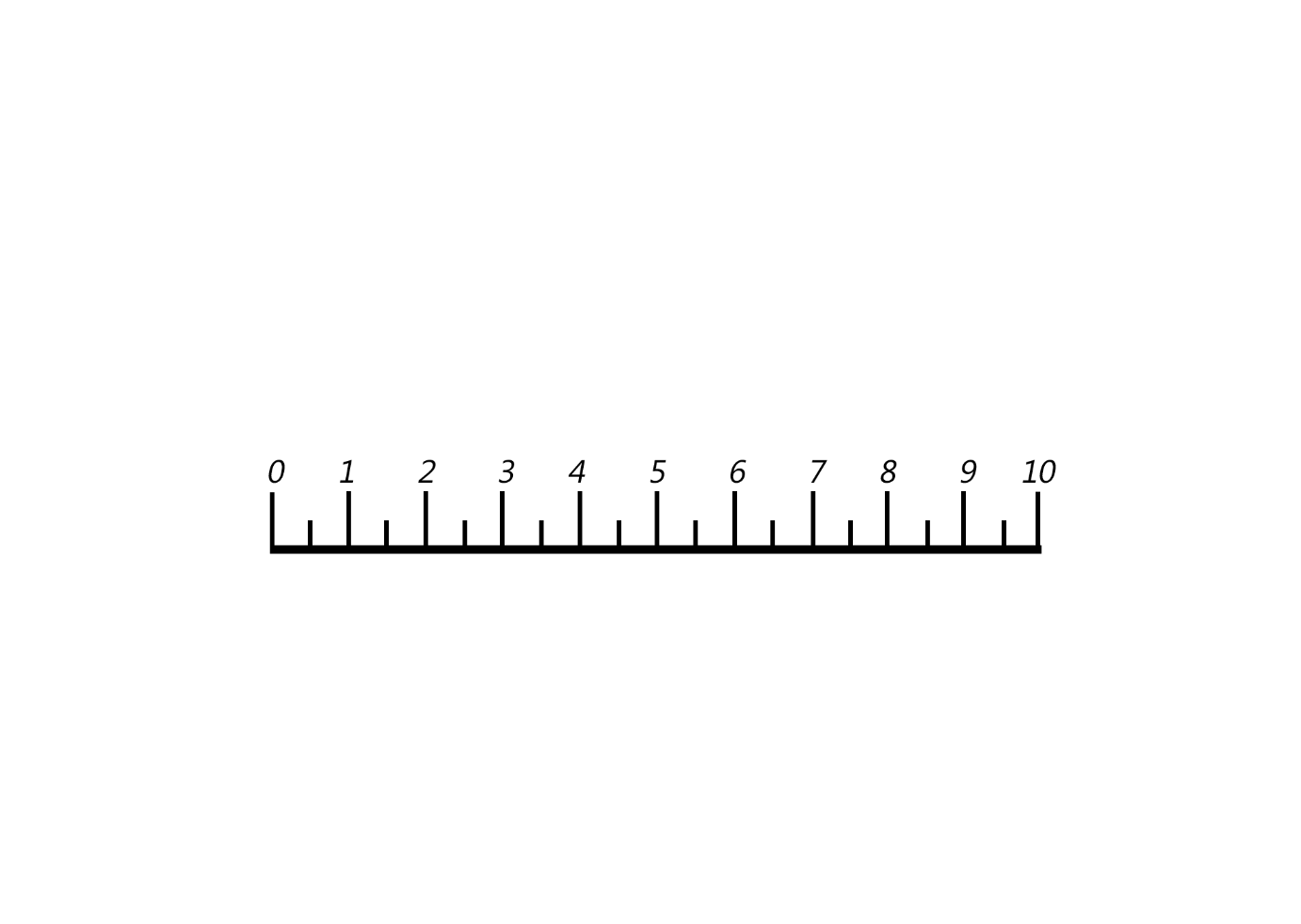


*How capable do I feel of managing this behavior?*


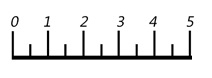


The person under your care keeps repeating the same questions dozens of times a day.

*Extent of behavior*


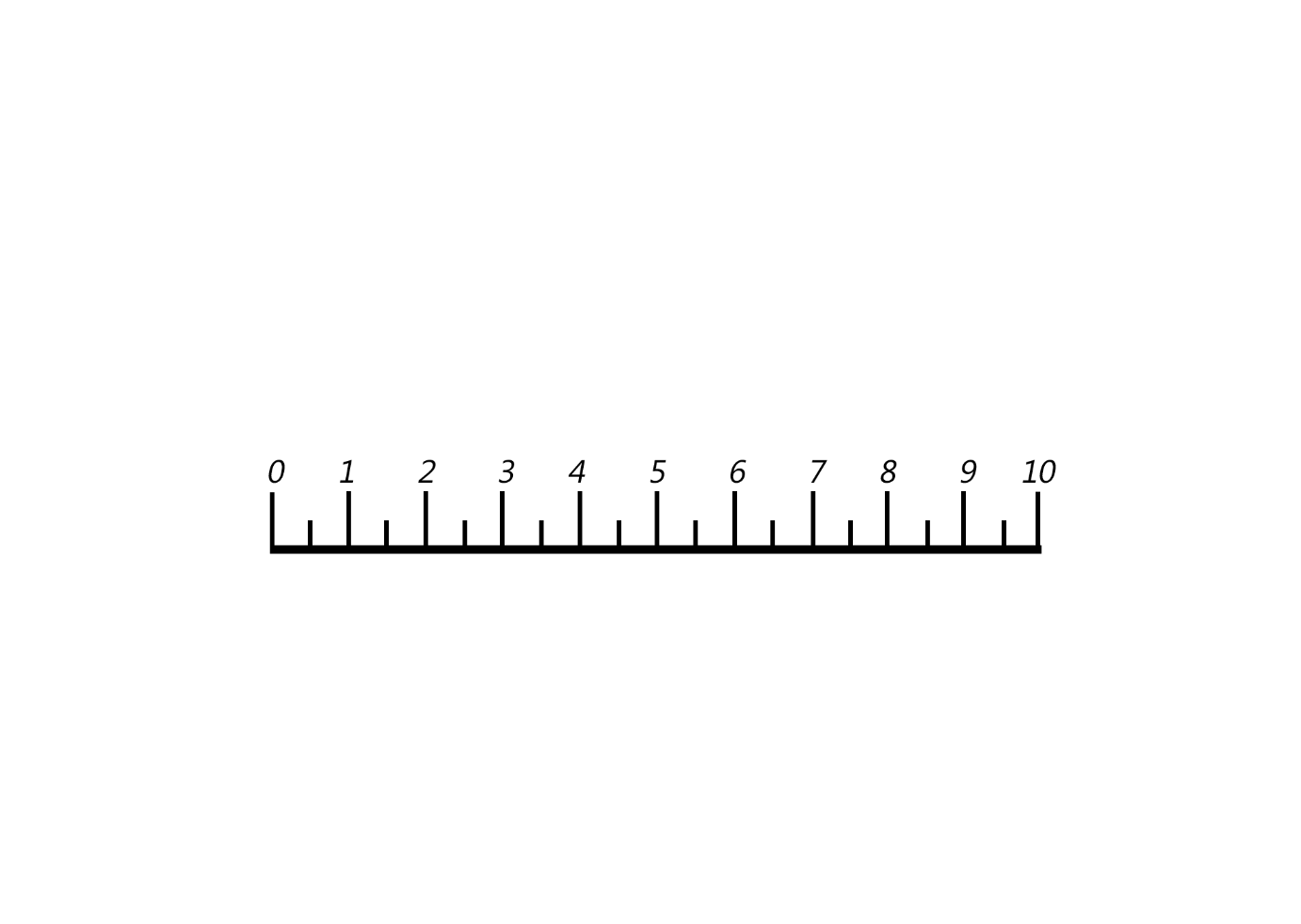


*How capable do I feel of managing this behavior?*


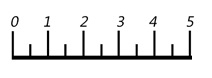


The person under your care:

1. must be stimulated to eat, otherwise s/he would feed insufficiently;
2. would eat all the time;
3. always craves sweets and tends to gain weight;
4. has developed bizarre food preferences, eats only highly selected food;
5. refuses to drink adequately;

*Extent of behavior*


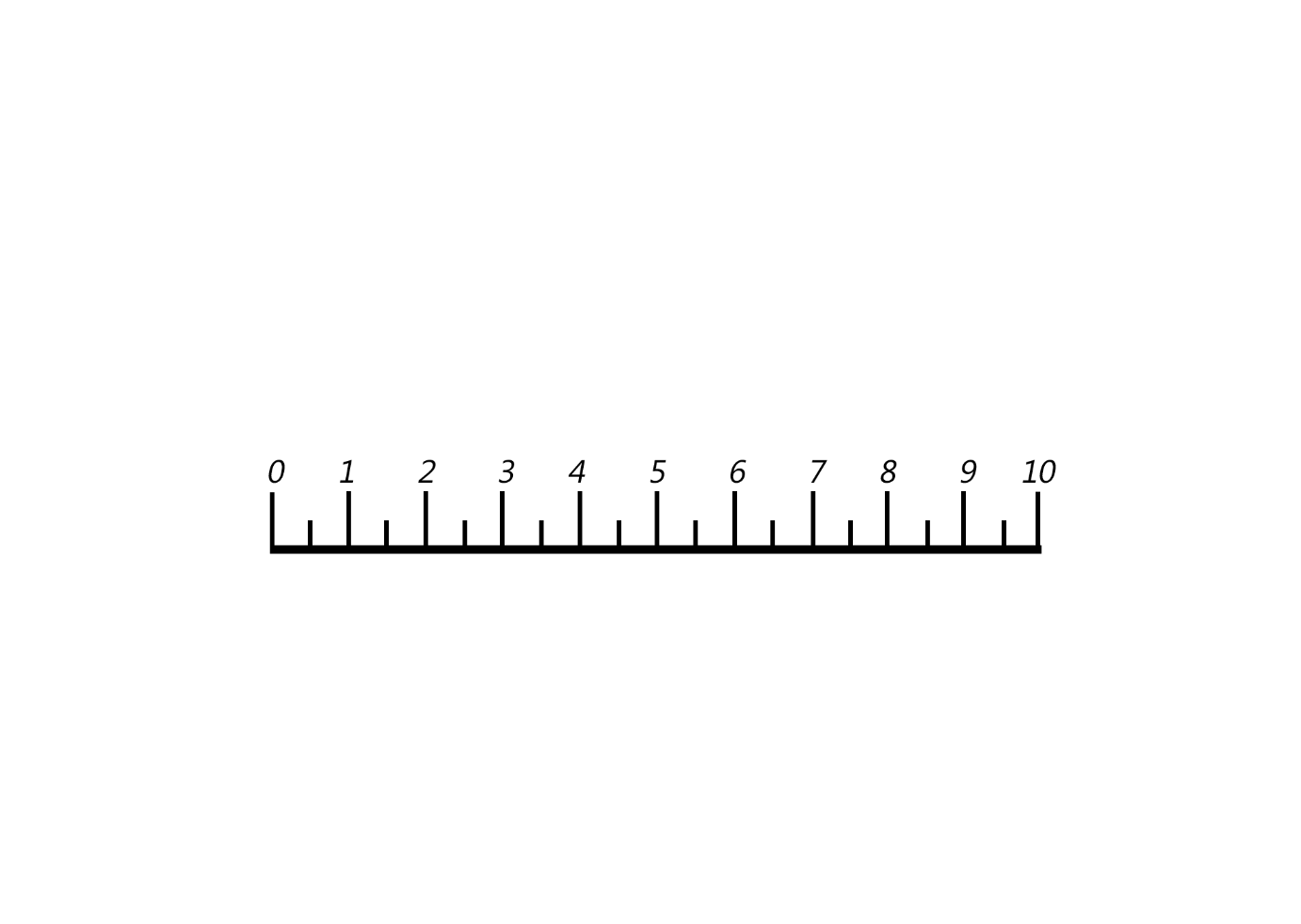


*How capable do I feel of managing this behavior?*


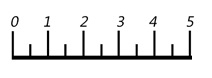


The person under your care:

1. is more agitated and confused in the afternoon and towards the evening;
2. experiences an increase in mood and behavior disturbances after dark;
3. these disturbances vary according to the season or the weather;

*Extent of behavior*


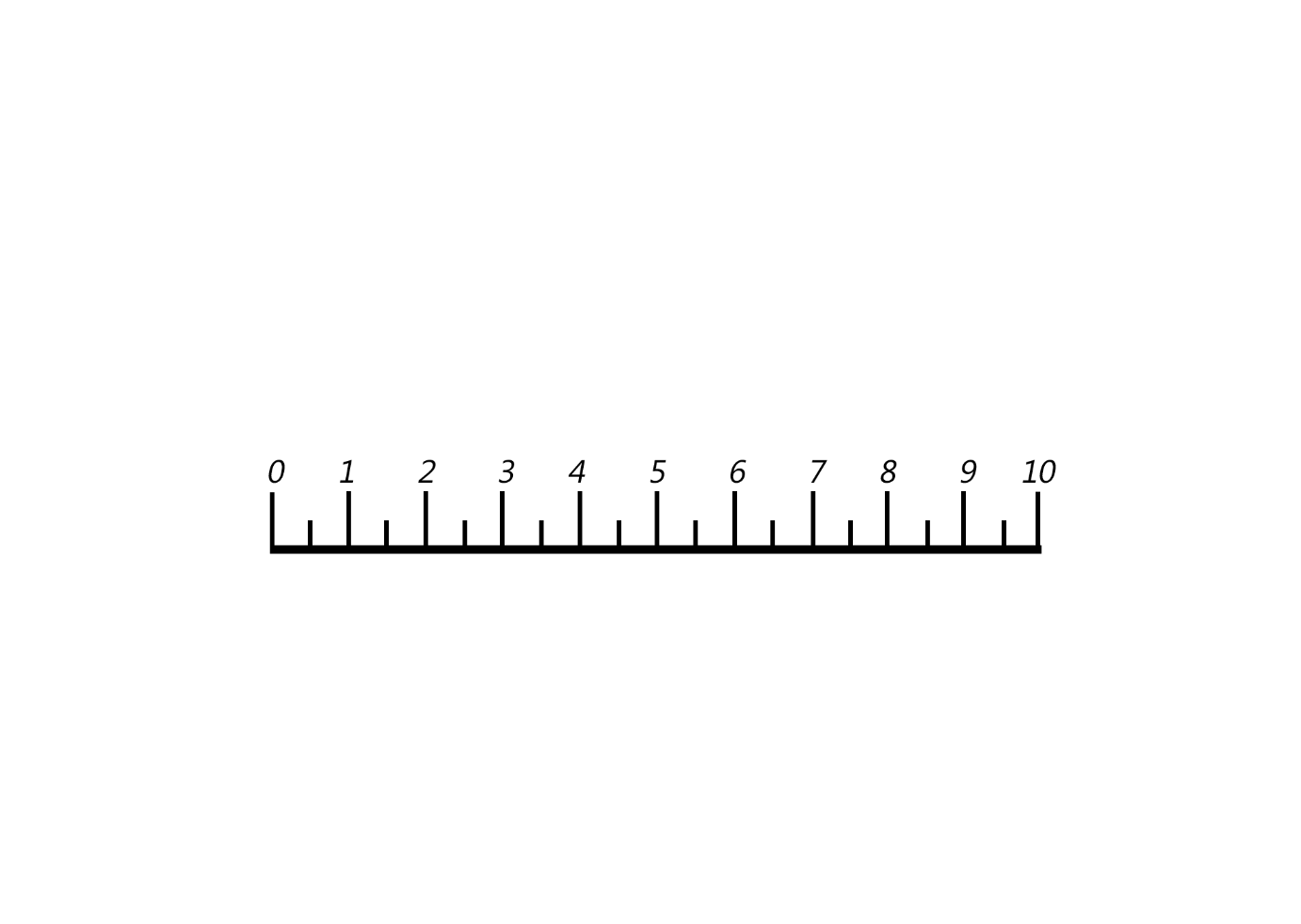


*How capable do I feel of managing this behavior?*


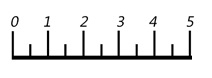


We thank you for answering the questions. Your responses will enhance the care of the person under your care.

Please indicate your age____

Education in years___________
Gender  M  F  Unspecified

Relationship with the person who assists:

- Spouse/partner
- Son/Daughter
- Brother/Sister
- Other family member
- Friend
- Professional caregiver

Are you the primary caregiver? Yes No

Are there other caregivers? Yes No

If yes, please indicate if the other caregivers are family members or professional caregivers

Do you live with the person under your care? Yes No

Where does the person you take care of live?

home

home but attends a day center

in a homecare

in an Alzheimer’s village
